# Chemical Evolution of Amphiphilic Xenopeptides for Potentiated Cas9 Ribonucleoprotein Delivery

**DOI:** 10.1021/jacs.3c01902

**Published:** 2023-07-03

**Authors:** Yi Lin, Xianjin Luo, Tobias Burghardt, Sarah Dorrer, Miriam Höhn, Ernst Wagner, Ulrich Lächelt

**Affiliations:** † Pharmaceutical Biotechnology, Center for Nanoscience, LMU Munich, Butenandtstr. 5-13, 81377 Munich, Germany; ‡ Department of Pharmaceutical Sciences, University of Vienna, UZA II, Josef-Holaubek-Platz 2, 1090 Vienna, Austria

## Abstract

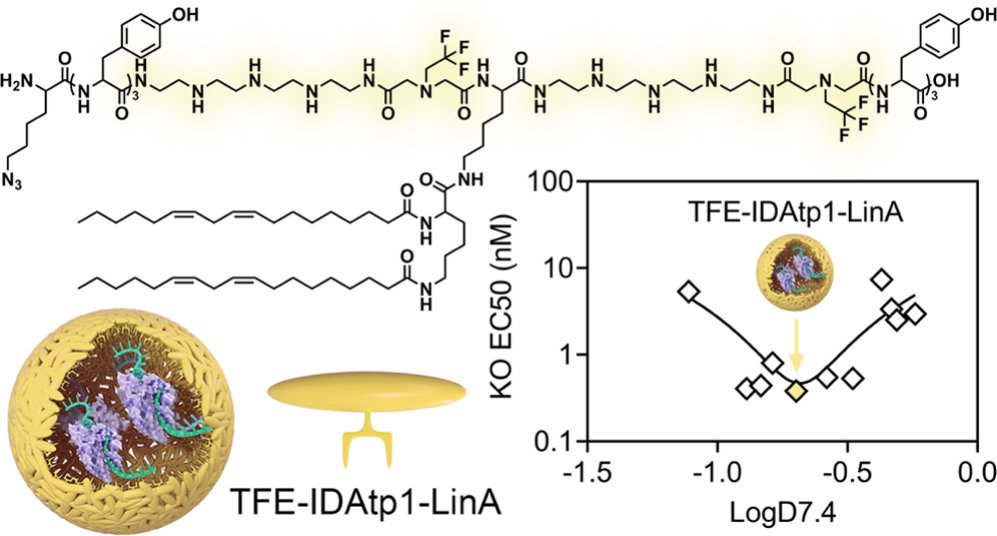

The introduction of the CRISPR/Cas9 system in the form of Cas9/sgRNA ribonucleoproteins (RNP) is an efficient, straightforward strategy for genome editing, and potent RNP carriers are in high demand. Here, we report a series of artificial peptides based on novel ionizable amino acids that are able to deliver Cas9 RNP into cells very efficiently. Systematic variation of hydrophobic properties revealed a relationship between the xenopeptide logD_7.4_ and genome editing potency. By correlating the physicochemical properties with biological activity, individual optima were found for different xenopeptide sequence architectures. The optimized amphiphilic carriers enable ∼88% eGFP knockout at an RNP dose of only 1 nM and up to 40% homology-directed repair (HDR) in eGFP/BFP switchable reporter cells by co-delivery with an ssDNA template. Mechanistic studies demonstrated that hydrophobically balanced xenopeptides are more resistant to ionic stress as well as concentration-dependent dissociation and promote endocytosis by both clathrin- and macropinocytosis-mediated pathways. The systematic study develops a versatile and adjustable carrier platform and highlights impactful structure–activity relationships, providing a new chemical guide for the design and optimization of nonviral Cas9 RNP nanocarriers.

## Introduction

The CRISPR/Cas9 system has become a fundamental gene editing technology in modern biomedical research.^1−3^ Introduction of the system into cells by plasmid DNA (pDNA) encoding Cas9 protein and sgRNA was initially the most widely used format; however, it is currently not preferred due to its low editing efficiency, rare but potentially harmful insertional mutagenesis, and higher risk of off-target events.^4,5^ Delivery of transiently expressed Cas9 mRNA is an alternative and, with lipid nanoparticles (LNP), a delivery technology is available, which has impressively demonstrated feasibility by the great success of mRNA vaccines.^6−8^ Instead of introducing these genetic “blueprints”, direct delivery of preassembled Cas9/sgRNA ribonucleoproteins (RNP) represents an alternative strategy that has gained much attention.^9,10^ It bypasses the transcription and translation processes and is immediately functional once delivered into cells. Similar to mRNA, RNP has a short exposure time to the cellular genome and can be rapidly eliminated, which decreases the risk of off-target effects to the utmost extent.^4,6^ Encouraged by the advantages of Cas9 RNP, numerous delivery strategies were developed based on diverse materials, including polymers,^11,12^ lipids,^13,14^ peptides,^15−17^ bioderived vesicles,^18^ DNA nanostructures,^19^ and inorganic or inorganic/organic hybrid systems.^20−22^ Peptides and peptide-like materials combine great design flexibility with feasible and highly precise synthesis procedures. Peptide-mediated delivery can be achieved either by covalent conjugation to the Cas9 protein^23^ or by noncovalent ionic interaction with negatively charged Cas9 RNP.^15−17^ In the case of covalent peptide conjugates, Cas9 RNP are exposed to the environment without protection, which may result in rapid degradation of the protein or sgRNA. In addition, the production of conjugates by conjugation chemistry or protein engineering is complex^24^ and faces the risk of bioactivity alteration.^25^ Therefore, the complexation of Cas9 RNP with peptides containing cationic domains via electrostatic interaction features advantages.^15−17,26,27^ While numerous strategies for gene knockouts via nonhomologous end joining (NHEJ) are available, achieving efficient DNA alteration via homology-directed repair (HDR) still presents a critical challenge. Herein, we report the synthesis of lipid-modified xenopeptides constructed from a series of novel artificial amino acids that are extremely potent for the intracellular delivery of Cas9 RNP to achieve efficient gene knockouts and knockins. Through systematic variation of peptide sequences and fine-tuning the hydrophobicity of artificial amino acids, the physicochemical properties of xenopeptides were modulated and the relationship between the octanol–water distribution coefficient (logD_7.4_) and green fluorescent protein (eGFP) reporter gene knockout as well as homology-directed repair driven conversion into blue fluorescent protein (BFP) was investigated.

## Results and Discussion

**Figure 1:**
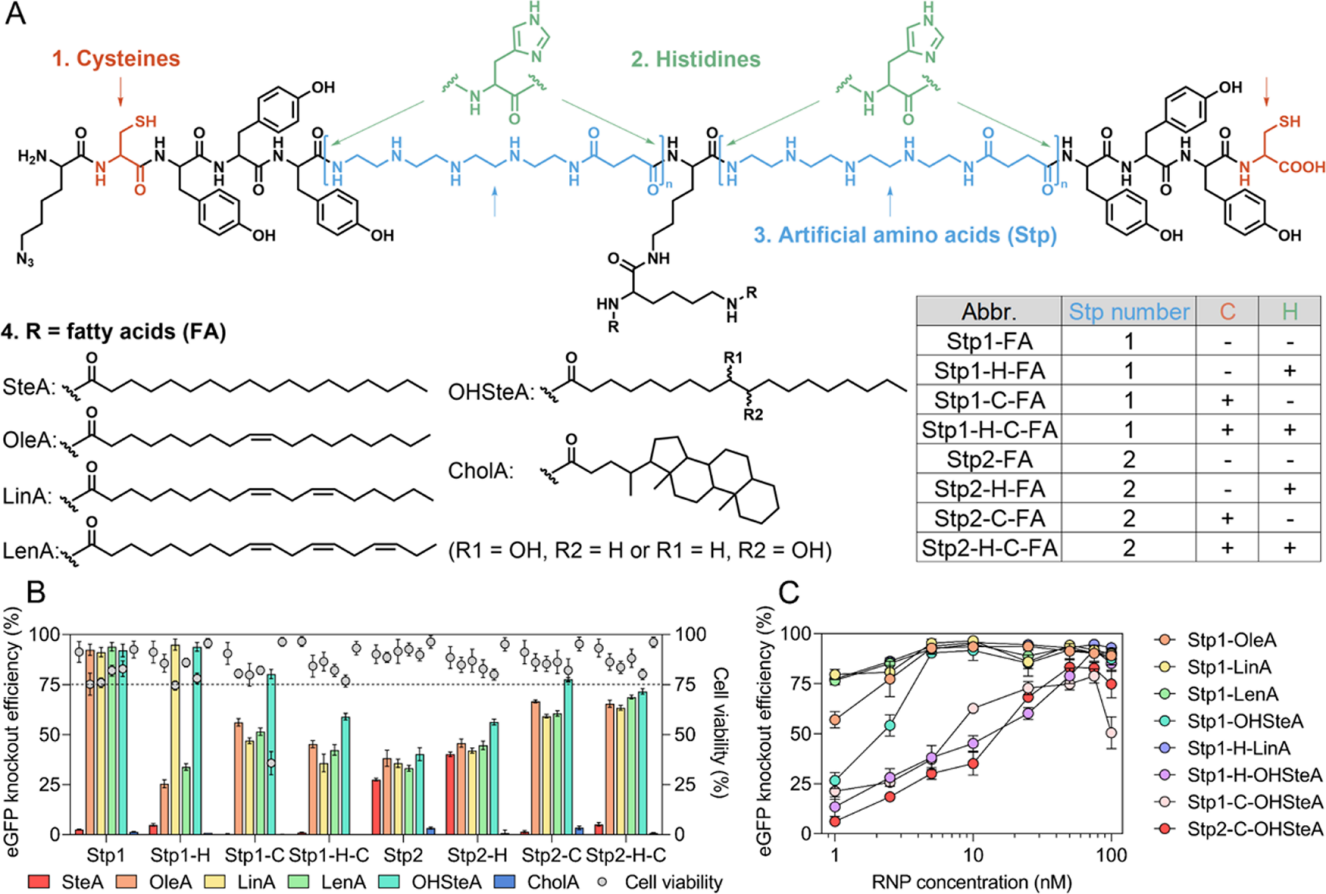
(A) Chemical structure of amphiphilic xenopeptides for Cas9 RNP delivery. “Stp number” indicates the number of ionizable Stp building units at each side of the lysine. (B) eGFP knockout efficiency and cell viability of HeLa eGFP/tub cells after 48 h treatment with Cas9 RNP nanocarriers at 75 nM RNP dose. Fatty acid residues are indicated by color code. (C) eGFP knockout efficiency of HeLa eGFP/tub cells after 48 h treatment with Cas9 RNP nanocarriers at a series of RNP concentrations ranging from 1 nM to 100 nM. Data are presented as mean ± SD (*n* = 3).

We have previously identified an artificial ionizable lipopeptide, **Stp2-C-OHSteA** (Figure 1A) generated by solid-phase peptide synthesis (SPPS) with the artificial amino acid Stp as an efficient RNP delivery carrier that is suitable for therapeutic genome editing applications.^16,17^

The artificial amino acid, succinoyl-tetraethylenepentamine (Stp), serves as an ionizable aminoethylene unit^28,29^ to complex negatively charged Cas9 RNP and to facilitate cellular delivery.^30,31^ The hydrophobic tyrosine (Y) tripeptide motif enhances the nanoparticle stability via hydrophobic interactions and improves transfection efficiency.^32^ Terminal cysteines (C) form bioreversible disulfide cross-links providing additional complex stabilization.^33^ Histidine, which was not contained in the previous lead structure, could be another potentially beneficial amino acid element due to protonation of the imidazole function at endosomal pH.^34,35^ Beyond that, fatty acid modification has been identified as an essential element that critically impacts delivery efficiency. In this work, the lead structure **Stp2-C-OHSteA** has been varied systematically in a chemical evolution process to elucidate structure–activity relationships and evolve the next generation of RNP carriers. In a first optimization screening, structural variations including the (1) presence of cysteine (C), (2) presence of histidine (H), (3) number of Stp units, and (4) type of fatty acid were introduced into the peptide sequence (Figure 1A). The Cas9 RNP nanocarriers were prepared by the straightforward complexation of amphiphilic xenopeptides with Cas9 RNP at an N/P ratio of 24, which resulted in nanoparticles with hydrodynamic sizes between 150 and 500 nm and positive ζ potentials between 10 and 19 mV, as determined by dynamic and electrophoretic light scattering (DLS, Table S2 and Figure S55).

The gene knockout efficiency and cytotoxicity of the nanocarriers were investigated in HeLa eGFP/tub cells after treatment with 75 nM RNP for 48 h (Figure 1B). The systematic evaluation demonstrated the effect of each variation. First, cysteine showed benefits in xenopeptides containing two Stp units at each side beyond the central lysine (Stp2 structures) but rather decreased the transfection efficiency in Stp1-based sequences carrying a single Stp at each side of the central lysine. Second, histidine, which has been previously shown to facilitate the endosomal escape of pDNA polyplexes,^34,35^ did not induce notable improvement in the case of RNP nanocarriers. Third, the Stp1 structures in general demonstrated a better knockout performance than the Stp2 structures. Lastly, in all cases, unsaturated (OleA, LinA, LenA) or hydroxyl-modified (OHSteA) fatty acids were essential to mediate efficient gene editing; saturated stearic acid (SteA) as well as the steroid cholanic acid (CholA) almost completely prevented knockout efficiency. Seven xenopeptides exhibited better or comparable knockout efficiency than the initial **Stp2-C-OHSteA**, and especially the Stp1 series outperformed the preceding lead structure. Over 90% of eGFP knockout was achieved with these structures at the dose of 75 nM RNP.

To differentiate the potency of the nanocarriers in more detail, a dose titration experiment was conducted in the concentration range of 1–100 nM RNP (Figure 1C). The lead structure **Stp2-C-OHSteA** showed a clear dose-dependent activity and eGFP knockout levels dropped below 40% at concentrations of 10 nM RNP or lower. The new xenopeptide analogues **Stp1-LinA**, **Stp1-LenA**, and **Stp1-H-LinA** turned out to be much more potent and induced ∼80% eGFP knockout even at the lowest RNP dose of 1 nM. Cell viability studies showed that all nanocarriers were generally well-tolerated except **Stp1-C-OHSteA** (Figure S56).

**Figure 2:**
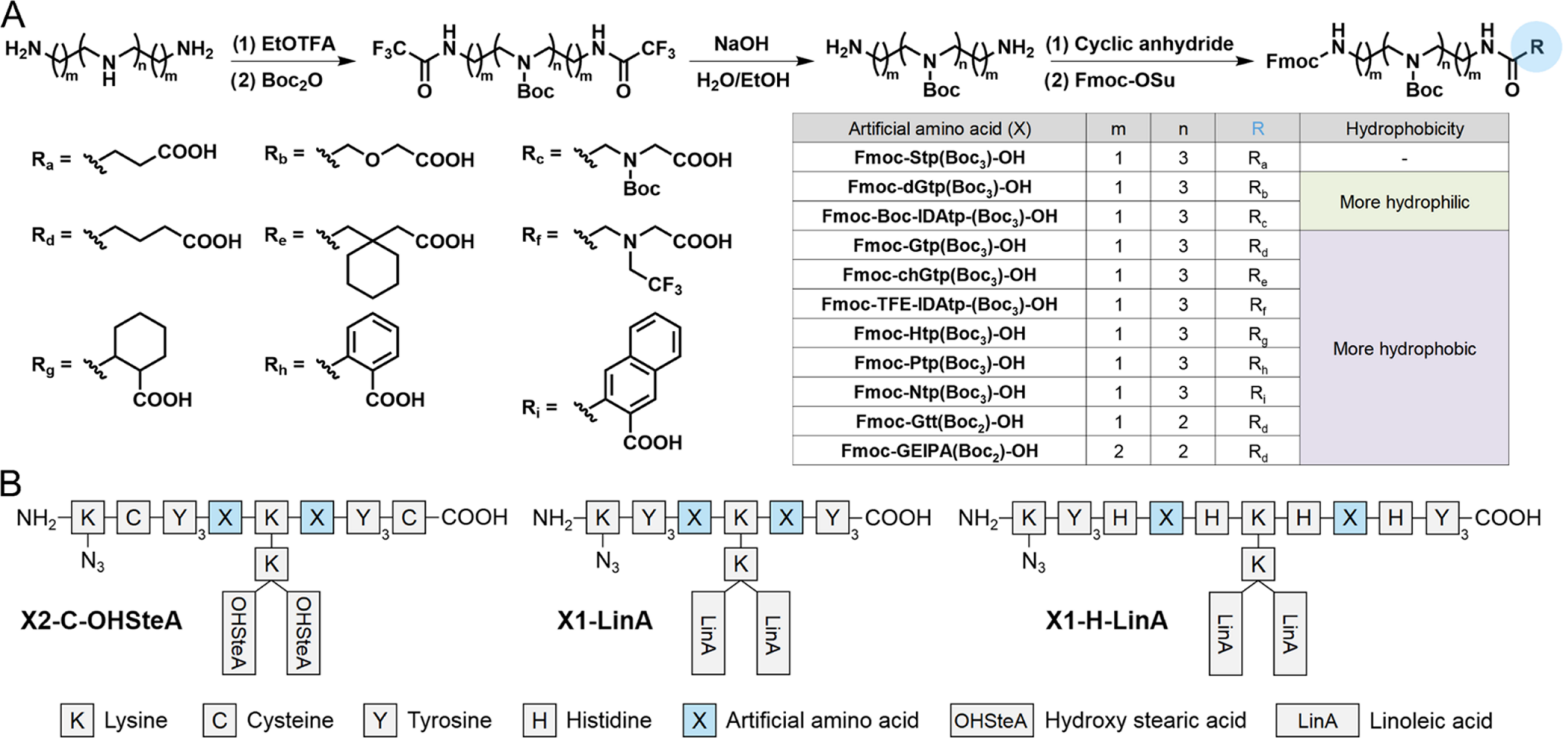
(A) General synthetic route of artificial amino acid building blocks with protective groups for use in solid-phase peptide synthesis (SPPS). (B) Amphiphilic xenopeptide architectures selected for the construction of hydrophobically balanced carriers.

Due to the generally more hydrophobic nature of the identified outperformers with lower content of ionizable artificial amino acid than the initial **Stp2-C-OHSteA**, we hypothesized that the hydrophobicity of the xenopeptides may be an important parameter to achieve efficient Cas9 RNP delivery. Consequently, the potency could be improved even further by fine-tuning the hydrophobic properties of the artificial amino acid units. To validate this hypothesis, a series of artificial amino acid building blocks with varied hydrophilic or hydrophobic structural elements and suitable protective groups for SPPS was synthesized (Figure 2A and Schemes S1 and S2).

Eight more hydrophobic Stp analogues were obtained by replacing tetraethylenepentamine (tp) by oligoamines with a lower number of ionizable nitrogens (triethylenetetramine, tt, or 3,3′-ethylenediiminodipropylamine, EIPA) and by including more hydrophobic dicarboxylic acids instead of succinic acid. Two more hydrophilic analogues were generated by replacing succinic acid with dicarboxylic acids containing an amine or ether group (Figure 2A). For a systematic evaluation of the new building blocks, the backbone sequences of the two best performers **Stp1-LinA** and **Stp1-H-LinA**, as well as the initial lead structure **Stp2-C-OHSteA**, were selected as reference architectures (Figure 2B).

**Figure 3:**
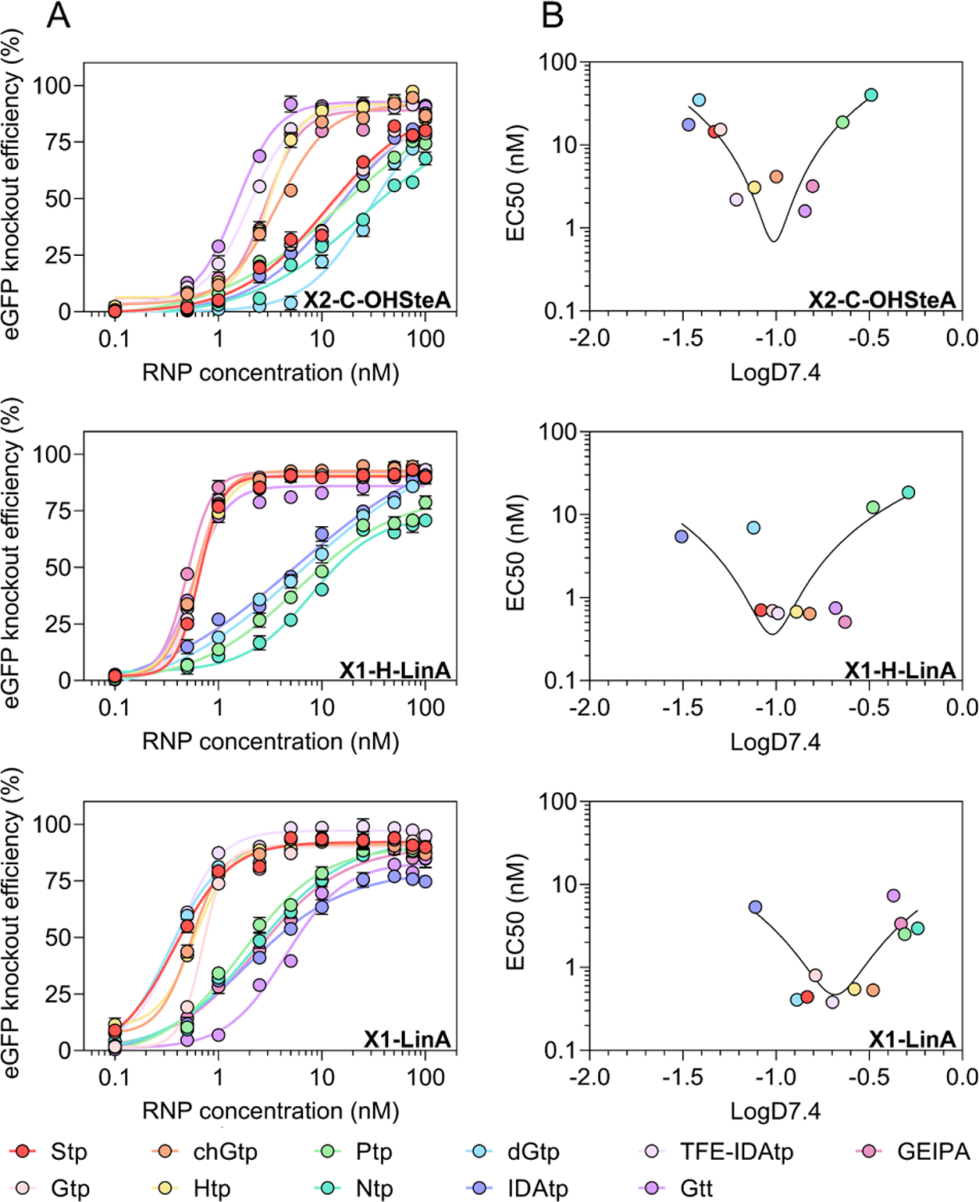
Dose titration of amphiphilic xenopeptides and logD_7.4_ correlation with eGFP knockout efficiencies. (A) eGFP knockout efficiency in HeLa eGFP/tub cells after 48 h treatment with Cas9 RNP nanocarriers at a series of RNP concentrations between 0.1 and 100 nM. Data are presented as mean ± SD (*n* = 3). (B) Plot of eGFP knockout EC50 values versus logD_7.4_ values of each xenopeptide series.

The combination of 11 building blocks with 3 architectures resulted in a total of 33 xenopeptides that were synthesized by SPPS (Tables S2 and S3). The logD_7.4_ of each xenopeptide was determined by quantifying the compound concentration in octanol and water phases by reversed-phase HPLC after mixing and phase separation (Scheme S3).^36^ The determined logD_7.4_ values (Table S3) indicated the following order of artificial amino acids with increasing hydrophobicity: IDAtp < dGtp < Stp < Gtp < TFE-IDAtp < Htp < chGtp < Gtt < GEIPA < Ptp < Ntp. Cas9 RNP nanocarriers were then formulated with the xenopeptides (N/P = 24), and the size, PDI, and ζ-potential were determined by DLS. All xenopeptides formed homogeneous nanoparticles with Cas9 RNP with a size of 140–190 nm, PDI of 0.15–0.38, and ζ-potential of 10.5–17.2 mV (Table S2). A dose titration study in the RNP concentration range between 0.1 and 100 nM was performed and the eGFP knockout EC50 of each xenopeptide was calculated (Figure 3A and Table S3).

In general, structures based on the X1-LinA architecture exhibited the best knockout efficiency, where 6 out of 11 xenopeptides achieved ∼75–80% eGFP knockout at 1 nM RNP dose and 3 out of 11 xenopeptides even induced over 50% eGFP disruption at 0.5 nM. The X1-H-LinA-based structures also demonstrated very high potency but slightly lower efficacy than X1-LinA derivatives at very low doses of RNP (0.5 nM). Notably, the performance of X2-C-OHSteA structures was greatly boosted by introducing more hydrophobic artificial amino acids (Gtt, GEIPA, Htp, chGtp, TFE-IDAtp). The best performers of each xenopeptide series were identified as **Gtt2-C-OHSteA**, **GEIPA1-H-LinA**, and **TFE-IDAtp1-LinA**. Especially, **TFE-IDAtp1-LinA** achieved up to 99% eGFP knockout at RNP concentrations down to 5 nM, ∼88% knockout at 1 nM, and still enabled ∼61% eGFP disruption at 500 pM. The high gene editing potency of **TFE-IDAtp1-LinA**, in comparison to the initial lead structure **Stp2-C-OHSteA**, was also demonstrated in two other reporter cell lines: murine colon carcinoma CT26 eGFP/luc and murine neuroblastoma N2a eGFP/luc (Figure S58). Both xenopeptides mediated higher gene knockouts than the commercial reagent Lipofectamine CRISPRMAX at 100 nM RNP and **TFE-IDAtp1-LinA** clearly showed the highest potency with ∼55% (CT26) and 13.4% (N2a) eGFP knockout at 1 nM RNP concentration. Cell viability studies in HeLa cells revealed that Htp- and chGtp-containing structures were toxic at high RNP concentrations (25–100 nM), while all other artificial amino acid-containing lipopeptides were well-tolerated (Figure S59). To correlate the obtained structure–activity relationships with hydrophobic characteristics, the logD_7.4_ was plotted against eGFP knockout EC50 values for each xenopeptide series (Figure 3B). A clear correlation between the logD_7.4_ and EC50 was found, and optimal logD_7.4_ ranges were identified for each sequence-defined lipopeptide series. The efficacy of structures with logD_7.4_ values beyond the optimal range (too hydrophilic or too hydrophobic) dramatically dropped.

**Figure 4:**
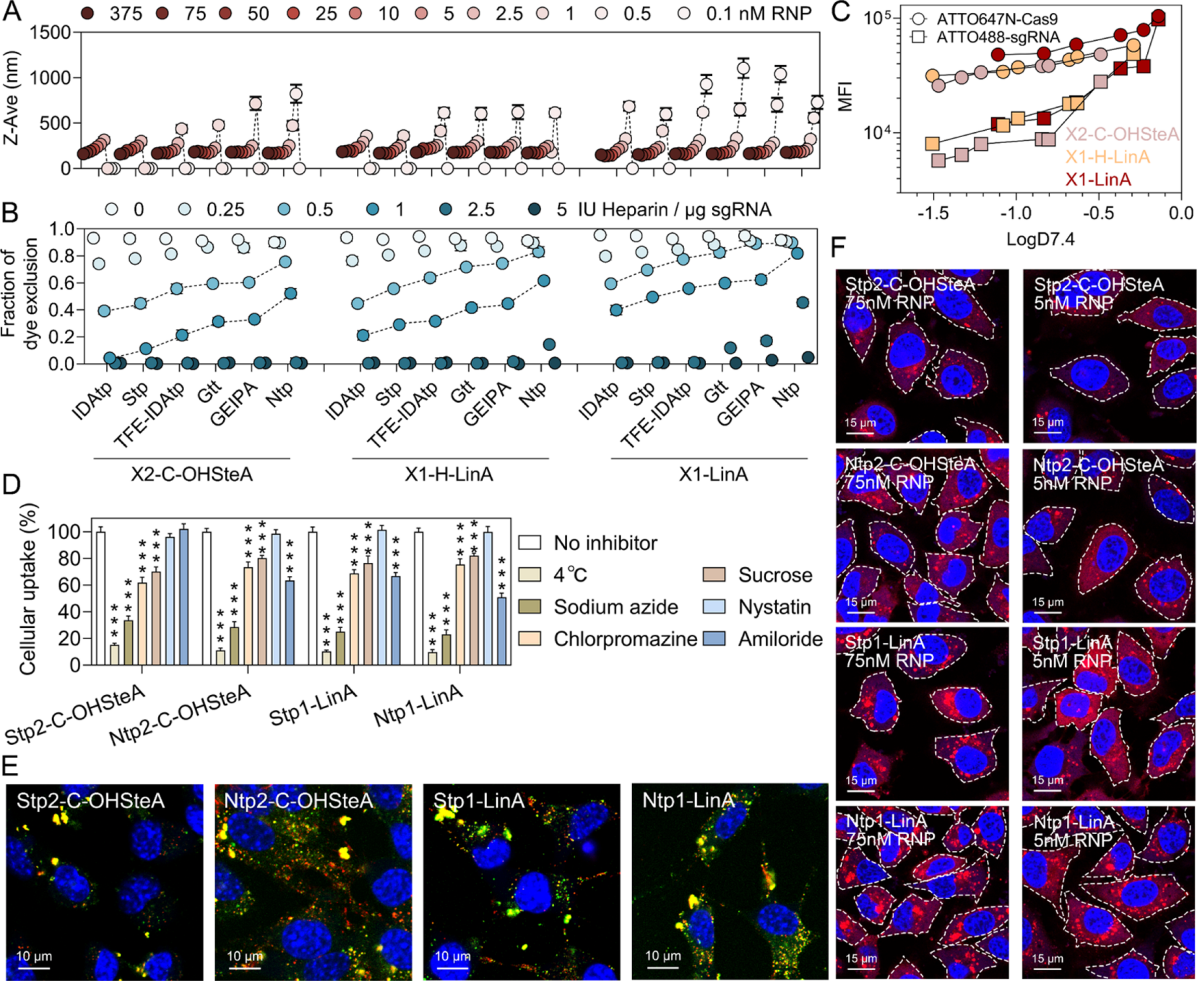
(A) Effect of dilution on the nanocarrier size determined by DLS; value = 0 indicates “not detectable”. (B) Nanocarrier stability against different concentrations of heparin (0, 0.25, 0.5, 1, 2.5, and 5 IU/μg sgRNA). Ribogreen was used for the detection of free RNA. (C) Plot of median fluorescence intensities (MFI) of ATTO647N-Cas9 protein and ATTO488-sgRNA determined by flow cytometry versus xenopeptide logD_7.4_ values of each xenopeptide series. (D) Endocytosis pathway study with different inhibitors. Sodium azide: energy-dependent endocytosis; chlorpromazine: clathrin-mediated endocytosis; sucrose: clathrin-mediated endocytosis; nystatin: caveolae-mediated endocytosis; amiloride: macropinocytosis. Data are presented as mean ± SD (*n* = 3). Statistical analysis was performed by comparing each treatment group with the corresponding “no inhibitor” group. ***p ≤ 0.001, **p ≤ 0.01, *p ≤ 0.05. (E) Confocal laser scanning microscopy (CLSM) images of HeLa WT cells 4 h after treatment with the selected Cas9 RNP nanocarriers (75 nM RNP) containing 20% of ATTO647N-Cas9 and 20% ATTO488-sgRNA. Nuclei were stained with DAPI (blue). The merged channel indicates co-localization (yellow) of ATTO647N-Cas9 (red) and ATTO488-sgRNA (green). (F) CLSM images of HeLa mRuby3/gal8 cells treated with the selected Cas9 RNP nanocarriers (75 and 5 nM RNP) for 4 h. Nuclei were stained with DAPI (blue). Red punctuate mRuby3/gal8 spots indicate endosomal membrane damage.

To explore the reason why hydrophobically balanced carriers are more potent, especially at low RNP concentrations, a group of representatives covering the spectrum from most hydrophilic to most hydrophobic was selected for investigation of the impact on critical carrier characteristics. The set of xenopeptides consisted of IDAtp-, Stp-, TFE-IDAtp-, Gtt-, GEIPA-, and Ntp-based structures. First, the integrity of the nanocarriers upon dilution was investigated (Figure 4A). The nanocarriers were prepared at a high RNP concentration of 375 nM and then sequentially diluted to concentrations ranging from 0.1 to 75 nM. The size of **IDAtp2-C-OHSteA** and **Stp2-C-OHSteA**, the two most hydrophilic X2-C-OHSteA structures, immediately increased upon dilution and was not detectable at an RNP concentration of 1 nM or lower. In contrast, the more hydrophobic analogues exhibited improved dilution stability. A similar tendency was observed with the more hydrophobic X1-H-LinA and X1-LinA architectures that were generally more resistant towards dilution. Nanoparticles generated with the most hydrophobic structure, **Ntp1-LinA**, retained a stable size between 180–220 nm at 2.5–375 nM of RNP and were still detectable at 0.1 nM. In addition, the stability of the nanocarriers against excessive amounts of ions was investigated by the detection of RNA release with the intercalating dye Ribogreen after exposure to sodium chloride (NaCl) and the polyanion heparin as ionic stress factors (Figures 4B and S60). At isotonic NaCl concentration (0.15 M), all of the nanocarriers showed robust encapsulation of Cas9 RNP. With increasing NaCl concentrations, higher Ribogreen fluorescence was detected, indicating lower dye exclusion and release of Cas9 RNP from dissociated nanocarriers (Figure S61). Notably, the more hydrophobic structures of each series exhibited better resistance against NaCl. Similar trends were found in the heparin competition assay, where hydrophobic artificial amino acids remarkably enhanced the nanocarrier stability against polyanionic stress (Figure 4B).

To examine the cellular uptake of hydrophobically balanced Cas9 RNP nanocarriers, flow cytometry experiments were performed with ATTO647N-labeled Cas9 and ATTO488-labeled sgRNA (Figures 4C and S60). The results demonstrated a general correlation of higher cellular uptake with increasing hydrophobic properties. Representative examples are the hydrophobic Ntp-based structures, where **Ntp2-C-OHSteA** and **Ntp1-LinA** achieved 4.4- and 7.3-fold enhanced sgRNA uptake compared to the Stp-based analogues. CLSM studies further confirmed the observation that **Ntp2-C-OHSteA** and **Ntp1-LinA** lead to higher Cas9 RNP uptake, indicated by the brighter intracellular fluorescence in HeLa cells (Figures 4E and S64). Furthermore, the endocytosis pathways of **Stp2-C-OHSteA**, **Ntp2-C-OHSteA**, **Stp1-LinA**, and **Ntp1-LinA** nanocarriers were probed by pretreating the cells with endocytosis inhibitors or incubating at 4 °C (Figures 4D and S63). Low temperature and sodium azide inhibited the uptake of all four nanocarriers by 85–90 and 65–77%, respectively, indicating an energy-dependent internalization mechanism. Chlorpromazine and sucrose were the only other inhibitors that significantly reduced the uptake of the more hydrophilic **Stp2-C-OHSteA** nanocarrier, suggesting a dominant role of clathrin-mediated endocytosis. Interestingly, the Cas9 RNP uptake mediated by more hydrophobic structures **Ntp2-C-OHSteA**, **Stp1-LinA**, and **Ntp1-LinA** could also be blocked by pretreatment with amiloride, which indicates a contribution of macropinocytosis in addition to the clathrin-mediated pathway. Notably, macropinosomes are reported to be more leaky than other endosomes, which may be beneficial for the translocation of nanoparticles into the cytosol.^37,38^ Following the intracellular delivery pathway stepwise, an endosomal escape reporter cell line HeLa mRuby3/gal8^39^ was used to evaluate the endosomal escape capability of the selected nanocarriers (Figures 4F and S65 and S67). In this model, the rupture of endosomal membranes results in the recruitment of a mRuby3/galectin-8 (gal8) fusion protein, which is visible by intracellular punctate red spots. After 4 h treatment, the Ntp-based structures were found to induce a higher number of endosomolytic events than their Stp counterparts, especially at a low RNP concentration (5 nM). Notably, the peptide architecture played an additional fundamental role in the endosomal escape process, since the X1-LinA sequences showed much better endosomal escape efficiency than the X2-C-OHSteA xenopeptides.

Taking the above results together, the mechanistic effects can be explained as follows: hydrophobic xenopeptides form Cas9 RNP nanocarriers, which (1) are more resistant toward dilution-mediated dissociation and remain intact at low concentrations; (2) are more resistant to ionic stress and avoid premature cargo release; (3) mediate more efficient cellular internalization that is driven by both clathrin-mediated endocytosis and macropinocytosis; and (4) possess a higher endosomal escape capacity.

The fact that optimal logD_7.4_ ranges and the requirement of an adequate balance have been observed demonstrates that “more is not always better”: in particular, too stable nanoparticles may result in insufficient cargo release in the right place at the right time.

**Figure 5:**
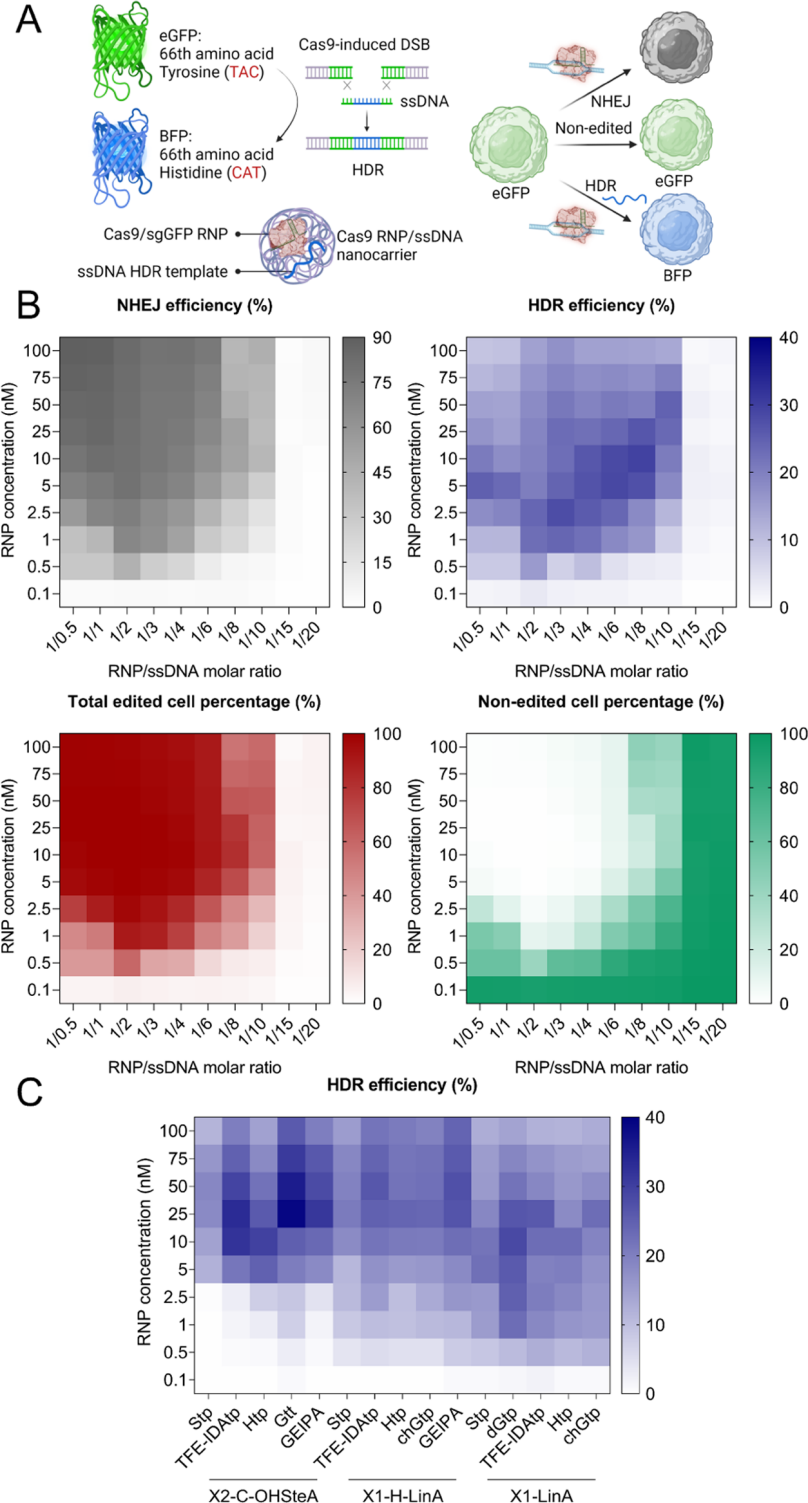
(A) Schematic illustration of eGFP to BFP conversion by co-delivery of Cas9 RNP and an ssDNA template into eGFP expressing cells. (B) Heat maps of NHEJ, HDR, total edited, and nonedited percentages in HeLa GFPd2 cells 48 h after treatment with Cas9 RNP/ssDNA nanocarriers (**TFE-IDAtp1-LinA**) at varied RNP concentrations and RNP/ssDNA ratios. (C) Heat map of HDR percentages in HeLa GFPd2 cells 48 h after treatment with different lipopeptide-based Cas9 RNP/ssDNA (fixed at 1/4) nanocarriers at varied RNP concentrations.

Having demonstrated that hydrophobically balanced xenopeptides are potent nanocarriers for delivering Cas9 RNP to mediate gene knockouts via nonhomologous end joining (NHEJ), we sought to extend the scope of application: the top five lipopeptides of each series were selected for the co-delivery of Cas9 RNP and the ssDNA template to mediate gene knockins via homology-directed repair (HDR). A HeLa cell line expressing destabilized eGFP (HeLa GFPd2)^40^ was used in this study. The 66th amino acid in the eGFP sequence, tyrosine (code: TAC), can be replaced by histidine (code: CAT) via HDR-mediated DNA repair, which results in the conversion of eGFP into BFP.^41,42^ Three cell populations can be expected after such treatments: (1) eGFP positive cells representing nonedited cells; (2) eGFP and BFP negative cells representing cells with NHEJ-mediated gene knockout; and (3) BFP positive and eGFP negative cells representing HDR-mediated gene-corrected cells (Figure 5A).

In the first step, **TFE-IDAtp1-LinA**, which performed best in gene knockout experiments, was used for finding the most suitable RNP/ssDNA ratios. The nanocarriers for HDR were prepared by complexing **TFE-IDAtp1-LinA** with different RNP/ssDNA ratios ranging from 1/0.5 to 1/20 at an N/P ratio of 12. Dynamic and electrophoretic light scattering showed, that **TFE-IDAtp1-LinA** formed nanoparticles with Cas9 RNP/ssDNA with hydrodynamic sizes between 169 and 111 nm and ζ-potentials between +10.9 and +22.5 mV (Figure S68). Within the range of investigated molar compositions, particle sizes decreased and ζ-potentials increased with increasing ssDNA content. Notably, the characteristics of Cas9 RNP and Cas9 RNP/ssDNA nanocarriers were unaffected by the prolonged complexation time of 40 vs 15 min (Figure S69).

HeLa GFPd2 cells were treated with the **TFE-IDAtp1-LinA** Cas9 RNP/ssDNA nanocarriers for 48 h, followed by cell population analysis via flow cytometry (Figure S70). As shown in Figures 5B and S71, the best knockout and total editing efficiencies were achieved at high RNP concentrations (10–100 nM) and high RNP/ssDNA ratios (1/0.5–1/3), while the highest HDR levels were induced at moderate to low RNP concentrations (10–1 nM) and moderate RNP/ssDNA ratios (1/3–1/8). At RNP/ssDNA ratios below 1/10, almost all editing events were blocked, presumably due to severe cytotoxicity (Figure S72). The highest HDR efficiency of ∼28% was achieved at a dose of 10 nM RNP and 1/8 RNP/ssDNA ratio. Notably, a general trend of increasing percentage of HDR events in relation to total editing was observed with decreasing RNP concentration, which is explainable by the competition between NHEJ and HDR repair pathways. Next, the HDR induction efficiency of the other selected xenopeptides was investigated at an RNP/ssDNA ratio of 1/4 to circumvent cytotoxicity (Figures 5C and S74–S77). Surprisingly, the X2-C-OHSteA series, which was the least efficient in gene knockout experiments, achieved the highest maximum HDR levels. Especially the hydrophobically balanced **Gtt2-C-OHSteA** enabled HDR induction up to 40% at a 25 nM RNP dose. Nevertheless, X1-LinA xenopeptides still showed the highest potency and best HDR efficiency at low concentrations. For instance, **TFE-IDAtp1-LinA** nanocarriers induced ∼23% and ∼11% HDR in HeLa GFPd2 cells treated with 1 or 0.5 nM RNP doses, respectively. The results demonstrate that the induction of HDR by the co-delivery of DNA templates adds an additional level of complexity, which requires the fine-tuning of the applied formulation components depending on the intended outcomes. Since gene knockouts via NHEJ and knockins via HDR require different biomolecular components (RNP or RNP/ssDNA), the most suitable carriers can vary for the different applications. Furthermore, the intended aims of achieving maximal gene editing levels within a certain concentration range or achieving the highest potency at low concentrations are different parameters, which have to be considered for selecting the most suitable delivery system.

## Conclusions

In summary, we have designed and synthesized a series of artificial amino acids and derived xenopeptides for the intracellular delivery of Cas9 RNP. The systematic biological evaluation revealed that the hydrophobic characteristics play a decisive role in potent gene editing, especially at low concentrations. The fluorinated amphiphilic xenopeptide **TFE-IDAtp1-LinA** was found to be a particularly potent nanocarrier that can achieve 88% NHEJ gene knockout and 23% HDR knockin at just 1 nM RNP concentration. The new artificial amino acids and xenopeptide architectures provide a versatile platform for the creation of highly potent and tunable cellular delivery agents. Furthermore, the identified relationships between logD_7.4_ values, carrier characteristics, and impact on cellular delivery are suggested to serve as a guide for the future design of Cas9 RNP nanocarriers.

## Supporting Information Available

Materials and experimental procedures; sgRNAs and ssDNA; synthetic routes of artificial amino acid building blocks; ^1^H NMR spectra; ESI-MS spectra of artificial amino acid building blocks; MALDI-TOF MS spectra of xenopeptides; gene editing efficiency and cell viability data; architecture, ID number, abbreviation, and logD_7.4_ of all xenopeptides and EC50 of Cas9 RNP nanocarriers; flow cytometry histograms of Cas9 protein and sgRNA uptake; nanocarrier stability; and confocal laser scanning microscopy images (PDF)
