# Supplementary material for: Chemical Evolution of Amphiphilic Xenopeptides for Potentiated Cas9 Ribonucleoprotein Delivery
Source: J Am Chem Soc. 2023 Jul 3;145(28):15171–9. doi: 10.1021/jacs.3c01902 (PMC10360056; doi:10.1021/jacs.3c01902)
Supplement: Supplementary file 1 — ja3c01902_si_001.pdf [file ja3c01902_si_001.pdf]

Supporting Information

Chemical Evolution of Amphiphilic Xenopeptides for Potentiated Cas9 Ribonucleoprotein Delivery

Yi Lin,[†] Xianjin Luo,[†] Tobias Burghardt,[†] Sarah Dorrer,[†] Miriam Höhn,[†] Ernst Wagner,[*,†] and Ulrich Lächelt[*,†,‡]

[†]Pharmaceutical Biotechnology, Center for Nanoscience, LMU Munich, Butenandtstrasse 5-13, 81377 Munich, Germany

[‡]Department of Pharmaceutical Sciences, University of Vienna, Josef-Holaubek-Platz 2, 1090 Vienna, Austria

[*]Corresponding Authors. Emails: ernst.wagner@cup.uni-muenchen.de; ulrich.laechelt@univie.ac.at

Table of Contents

Materials 2
Methods 3
Experimental 5
Supporting Figures and Tables 12
References 61

Materials

Tetraethylenepentamine (TEPA), triethylenetetramine (TETA), 3,3'-ethylenediiminodipropylamine (EIPA), 2-chlorotrityl chloride resin (200-400 mesh, 1 % DVB crosslinking), 1-hydroxybenzotriazole (HOBT), di-tert-butyl dicarbonate (Boc_2O), stearic acid (SteA), oleic acid (OleA), linoleic acid (LinA), linolenic acid (LenA), 5β-cholanic acid (CholA), chloroform-*d*, deuterium oxide and Triton X-100 were purchased from Sigma-Aldrich (Munich, Germany). Succinic anhydride, glutaric anhydride, diglycolic anhydride, phthalic anhydride, 1,1-cyclohexanediacetic anhydride, hexahydrophthalic anhydride, and naphthalic anhydride were purchased from Sigma-Aldrich (Munich, Germany). (Benzotriazol-1-yloxy)tripyrrolidinophosphonium hexafluorophosphate (Pybop®) was purchased from Multisyntech (Witten, Germany). Lipofectamine CRISPRMAX was purchased from Invitrogen (Carlsbad, USA). All fmoc-α-amino acids were obtained from Iris Biotech (Marktredwitz, Germany). Ethyl cyano(hydroxyimino)acetate (Oxyma) and N, N'-diisopropylcarbodiimide (DIC) were purchased from Iris Biotech (Marktredwitz, Germany). Kaiser test solution was prepared using 80 % (w/v) phenol in ethanol, 5 % (w/v) ninhydrine in ethanol, and 20 µM potassium cyanide in pyridine. All chemical reagents and solvents were used as received without further purification. Cas9 protein was produced in house. Single guide RNA (sgRNA, 2' O-methyl modification on the first 3 and last 3 RNA bases) and single stranded DNA (ssDNA) were purchased from Integrated DNA Technologies (IDT, USA), the sequences of sgRNA and ssDNA are shown in Table S1. Dulbecco's Modified Eagle's Medium (DMEM), penicillin/streptomycin and fetal bovine serum (FBS) were purchased from Life Technologies (Carlsbad, USA). 3-(4,5-Dimethylthiazol-2-yl)-2,5-diphenyltetrazolium bromide (MTT), 4′,6-diamidino-2-phenylindole (DAPI), and paraformaldehyde (PFA) were obtained from Sigma-Aldrich (Munich, Germany). HEPES buffered glucose solution (HBG, pH 7.4) was prepared using 20 mM HEPES and 5 % (w/v) glucose. Deionized water was purified in-house using a Millipore system (Simplicity Plus, Millipore Corp.) and was used for all experiments.

Table S1. sgRNAs and ssDNA used in this study.

sgRNA/ssDNA	Sequences (5' → 3')
sgGFP1[a]	mG*mA*mC*rCrArGrGrArUrGrGrGrCrArCrCrArCrCrCrGrUrUrUrArGrArGrCrU-rArGrArArArUrArGrCrArArGrUrUrArArArArUrArArGrGrCrUrArGrUrCrCrGrUrUrArUrCrArArCrUrUrGrArArArArArGrUrGrGrCrArCrCrGrArGrUrCrGrGrUrGrCmU*mU*mU*rU
sgGFP2[b]	mG*mC*mU*rGrArArGrCrArCrUrGrCrArCrGrCrCrGrUrGrUrUrUrUrArGrArGrCrU-rArGrArArArUrArGrCrArArGrUrUrArArArArUrArArGrGrCrUrArGrUrCrCrGrUrUrArUrCrArArCrUrUrGrArArArArArGrUrGrGrCrArCrCrGrArGrUrCrGrGrUrGrCmU*mU*mU*rU
sgCtrl	mG*mG*mG*rUrArArCrCrGrUrGrCrGrGrUrCrGrUrArCrGrUrUrUrUrArGrArGrCrU-rArGrArArArUrArGrCrArArGrUrUrArArArArUrArArGrGrCrUrArGrUrCrCrGrUrUrArUrCrArArCrUrUrGrArArArArArGrUrGrGrCrArCrCrGrArGrUrCrGrGrUrGrCmU*mU*mU*rU
ssDNA	G*C*CACCTACGGCAAGCTGACCCTGAAGTTCATCTGCACCACCGGCAAGCTGCCCGTGCCCTGGCCCACCCTCGTGACCACCCTGAGCCACGGCGTGCAGTGCTTCAGCCGCTACCCCGACCACAT*G*A

[a] sgGFP1 was used in all eGFP knockout experiments. [b] sgGFP2 was used in all homology-directed repair (HDR) mediated eGFP to BFP conversion experiments.

Methods

^{1}H Nuclear magnetic resonance (^{1}H NMR) spectroscopy

^{1}H NMR spectra were recorded on an Advance III HD Bruker BioSpin (400 MHz) in chloroform-*d* ($CDCl_3$), deuterium oxide (D_2O) or methanol-*d*4 (CD_3OD) without TMS. Chemical shifts were calibrated to the residual solvent signal and were reported in parts per million (ppm). The spectra were processed in MestReNova software (MestReLab Research, SL). Integration was performed manually.

Electrospray ionization mass spectrometry (ESI-MS)

ESI mass spectra were recorded with a Thermo Scientific LTQ FT Ultra fourier transform ion cyclotron and an IonMax source in chloroform or methanol.

Matrix-assisted laser desorption/ionization mass spectrometry (MALDI-MS)

MALDI mass spectra of xenopeptides were recorded on an Autoflex II mass spectrometer (Bruker Daltonics, Bremen, Germany) in the positive ion mode. 10 mg/mL Super-DHB in H_2O/ACN/TFA (69.93:30:0.07) was used as matrix solution. 1 µL of the matrix solution was spotted on an MTP AnchorChip (Bruker Daltonics, Bremen, Germany). After drying of the matrix, 1 µL of xenopeptide solution (1 mg/mL in water) was added to the dried matrix.

Size-exclusion chromatography (SEC)

Purification of xenopeptides was carried out by SEC using a Äkta purifier system (GE Healthcare Bio-Sciences AB, Uppsala, Sweden) equipped with a P-900 solvent pump module, a UV-900 UV/VIS multi-wavelength detector, a pH/C-900 conductivity module and a Frac-950 automated fraction collector. Sephadex G-10 (MWCO 700 Da) was used as the gel filtration resin and 10 mM HCl solution/acetonitrile (v/v, 7/3) as the mobile phase.

Analytical reverse phase high-performance liquid chromatography (RP-HPLC)

The octanol/water distribution of xenopeptides was analyzed by RP-HPLC on a VWR Hitachi Chromaster HPLC system equipped with a 5160 pump module, a 5260 auto sampler, a 5310 column oven, and a 5430 diode array detector. 50 µL of xenopeptides in octanol or water were analyzed using a YMC C18 column (HS-302, HS12S05-1546WT, 5 µm, 4.6 x 150 mm, 12 nm, YMC Europe GmbH, Dinslaken, Germany) and a water/acetonitrile gradient (95:5 - 0:100) containing 0.1 % TFA. The extinction at 280 nm was monitored.

Dynamic light scattering (DLS) and zeta potential analysis

The hydrodynamic particle size and zeta potential of nanoparticles were measured in folded capillary cells (DTS 1070) using a Zetasizer Nano ZS (Malvern Instruments, UK). The scattering angle of 173° was fixed, the refractive index (RI) of the solvent was 1.330, the viscosity was 0.8872 mPa·s, and the temperature was set to 25 °C. All samples were measured 3 times with 12-15 sub-runs.

Ribogreen assay

sgRNA amount in naked Cas9/sgRNA ribonucleoproteins (Cas9 RNPs) or Cas9 RNP nanocarriers were determined using a Quant-iT^{TM} RiboGreen$^{®}$ RNA assay kit (Invitrogen, CA, USA). 50 µL of naked Cas9 RNPs or Cas9 RNP nanocarriers containing 250 ng Cas9 protein and 50 ng sgRNA were mixed with 50 µL of TE buffer and were added to the 96-well plate. The plate was incubated for 10 min. Afterwards, 100 µL of Ribogreen solution (diluted 1:100 in TE buffer) was added to each well. The plate was incubated for another 5 min. Ribogreen fluorescence intensity was detected using a microplate reader (Spectrafluor Plus, Tecan, Männedorf, Switzerland) with excitation and emission wavelength of 485 and 528 nm, respectively.

Cell culture

HeLa WT, HeLa eGFP/tub, HeLa GFPd2 (destabilized eGFP), CT26 eGFP/luc and N2a eGFP/luc cells were grown in DMEM medium supplemented with 10 % FBS, 100 U/mL penicillin, and 100 µg/mL streptomycin. The cells were cultured in ventilated flasks in the cell incubator at 37 °C and 5 % CO_2 in a humidified atmosphere. The cells were passaged at a confluency of approximately 80 %.

Flow cytometry

Cells were harvested and resuspended in 600 µL of FACS buffer (PBS buffer containing 10 % FBS). The samples were analyzed by flow cytometry on a CytoFLEX S flow cytometer (Beckman Coulter, CA, USA). For eGFP knockout and cellular uptake experiments, 0.6 µL of 1 µg/µL DAPI was added to each sample for differentiating live and dead cells before the measurement. The DAPI signal and BFP fluorescence were detected with 405 nm excitation and 450 nm emission. The eGFP and ATTO488 fluorescence was assayed with 488 nm excitation and 530 nm emission. The ATTO647N fluorescence was assayed with 640 nm excitation and 670 nm emission. Ten thousand of isolated live cells were counted and evaluated. The data were analyzed using FlowJo 7.6.5 by FlowJo, LLC (Becton, Dickinson and Company, USA).

MTT assay

Cell viability was determined using an MTT assay. After different treatments, 10 µL of MTT (5 mg/mL) were added to each well. Cells were incubated for 2 h. Afterwards, medium was removed and the plate was frozen at - 80 °C for 4 h. 100 µL of DMSO were added to each well. The plate was incubated for another 30 min at 37 °C under constant shaking. The absorbance was measured at 590 nm with background correction at 630 nm using a microplate reader (Tecan Spark 10M, Tecan, Männedorf, Switzerland). The relative cell viability (%) was calculated relative to control wells treated with HBG buffer as ([A] test/[A] control) × 100 %.

Confocal laser scanning microscopy (CLSM)

CLSM images were recorded on a Leica-TCS-SP8 confocal laser scanning microscope equipped with a HC PL APO 63× 1.4 objective (Germany). DAPI emission was recorded at 450 nm, mRuby3 at 590 nm, ATTO647N-Cas9 at 670 nm, and ATTO488-sgRNA at 520 nm. All images were processed using the LAS X software from Leica.

Experimental

Synthesis of *N*-(*tert*-Butoxycarbonyl)iminodiacetic acid (Boc-IDA)

N-(*tert*-Butoxycarbonyl)iminodiacetic acid (Boc-IDA) was synthesized as previously reported with slight variations.[1] In brief, 13.3 g of iminodiacetic acid (0.1 mol, 1 eq) and 200 mL 1,4-dioxane were added into a 1 L round-bottom flask. 200 mL NaOH solution (1 M) were added and the mixture was stirred until a clear solution formed. 24.0 g of di-*tert*-butyl dicarbonate (0.11 mol, 1.1 eq) dissolved in 50 mL 1,4-dioxane were added and the mixture was stirred at RT for 72 h. Afterwards, the mixture was concentrated to approximately 200 mL under reduced pressure, washed twice with 150 mL diethyl ether. 100 mL 10 % HCl were then added to acidify the mixture. The product was extracted with ethyl acetate (3 x 150 mL). The organic phases were collected and dried over anhydrous sodium sulfate. The ethyl acetate was filtrated and evaporated to obtain 21.8 g of Boc-IDA (0.094 mol, 94 %) as white crystals.

***N*-(*tert*-Butoxycarbonyl)iminodiacetic acid (Boc-IDA).** Yield: 94 %. ^{1}H NMR (400 MHz, CD_3OD) δ (ppm) = 4.10 - 3.95 (m, 4H, C**H_2**), 1.49 - 1.36 (s, 9H, C**H_3**-*tert*-but). ESI-MS: calc. for 233.1; $[M-H]^-$ found 232.1.

Synthesis of *N*-(*tert*-Butoxycarbonyl)iminodiacetic acid anhydride (Boc-IDA anhydride)

5.0 g of Boc-IDA (21.5 mmol, 1 eq) and 250 mL DCM were added into a 500 mL round-bottom flask. 4.45 g of dicyclohexylcarbodiimide (21.5 mmol, 1 eq) dissolved in 50 mL DCM were then added and the mixture was stirred at RT overnight. Afterwards, the mixture was concentrated to approximately 100 mL under reduced pressure. The insoluble dicyclohexyl urea was removed by filtration. The obtained solution was evaporated to yield 4.3 g of Boc-IDA anhydride (19.9 mmol, 93 %) as a solid. The synthesis procedures of Boc-IDA anhydride are shown in Scheme S1A.

Synthesis of *N*-(Trifluoroethyl)iminodiacetic acid (TFE-IDA)

TFE-IDA was synthesized analog to the synthesis of methyliminodiacetic acid reported by Berchet with modification.[2] 20.0 g of chloroacetic acid (211.6 mmol, 2 eq) were put into a 500 mL round-bottom flask. 20 mL H_2O were added and the flask was cooled in an ice bath. 93 mL cold NaOH (21.1 g, 529 mmol, 5 eq) solution were added slowly under stirring. Afterwards, the cooling bath was removed and 14.3 g of trifluoroethylamine hydrochloride (105.8 mmol, 1 eq) dissolved in 65 mL H_2O were added dropwise. After complete addition, the mixture was stirred overnight. The next day 45 g of $BaCl_2$ dihydrate in 100 mL boiling H_2O were added and the mixture was heated for 1.5 hours. The solid was filtered off and dried to yield 20.5 g of TFE-IDA barium salt (58.6 mmol). The TFE-IDA barium salt was put into a 500 mL round-bottom flask. 50 mL H_2O were added and heated to boiling. 23.4 mL H_2SO_4 solution (2.5 M, 58.6 mmol) were added slowly under constant heating to boiling. After cooling to RT, the mixture was centrifuged to remove the solid $BaSO_4$. The supernatant was freeze-dried to yield 16.1 g of TFE-IDA (55.7 mmol, 53 %) as colorless crystals.

***N*-(Trifluoroethyl)iminodiacetic acid (TFE-IDA).** Yield: 53 %. ^{1}H NMR (400 MHz, D_2O) δ (ppm) = 3.70 - 3.56 (m, 4H, C**H_2**), 3.52 - 3.29 (q, 2H, C**H_2**CF_3). ESI-MS: calc. for 215.0; $[M-H]^-$ found 214.0.

Synthesis of *N*-(Trifluoroethyl)iminodiacetic acid anhydride (TFE-IDA anhydride)

5.0 g TFE-IDA (23.2 mmol) and 140 mL DCM were put into a 250 mL round-bottom flask. 4.8 g dicyclohexylcarbodiimide (23.2 mmol, 1 eq) dissolved in 30 mL DCM were added into the flask. The mixture was stirred at RT overnight. The next day the mixture was concentrated to approximately 80 mL under reduced pressure. The insoluble dicyclohexyl urea was removed by filtration. The obtained solution was evaporated to yield 4.2 g TFE-IDA anhydride (21.3 mmol, 92 %) as a solid. The synthesis procedures of TFE-IDA anhydride are shown in Scheme S1B.

Synthesis of artificial amino acid building blocks

The synthesis procedures of artificial amino acid building blocks are shown in Scheme S2. Fmoc-Stp(Boc_3)-OH, Fmoc-Gtp(Boc_3)-OH, and Fmoc-Gtt(Boc_2)-OH were synthesized as previously reported.[3] Fmoc-Boc-IDAtp(Boc_3)-OH, Fmoc-dGtp(Boc_3)-OH, Fmoc-Htp(Boc_3)-OH, Fmoc-chGtp(Boc_3)-OH, Fmoc-TFE-IDAtp(Boc_3)-OH, Fmoc-GEIPA(Boc_2)-OH, Fmoc-Ptp(Boc_3)-OH, and Fmoc-Ntp(Boc_3)-OH were synthesized according to the protocol described below. In brief, the two primary amines of TEPA or EIPA (1 eq) were selectively protected with ethyl trifluoroacetate (EtOTFA, 2.5 eq). Subsequently the remaining secondary amines were Boc-protected by reaction with di-tert-butyl dicarbonate (Boc_2O, 4 eq) in a one-pot reaction. Work-up and recrystallization gave the compounds bis-tfa-TEPA(Boc_3) or bis-tfa-EIPA(Boc_2), respectively. The primary amines were deprotected by alkaline hydrolysis with aqueous NaOH containing 45 % ethanol to obtain the compounds TEPA(Boc_3) or EIPA(Boc_2). In the final step, the two primary amines were asymmetrically substituted by reaction with a cyclic anhydride (1.25 eq, phthalic anhydride for Fmoc-Ptp(Boc3)-OH; 3-oxaspiro[5,5]undecan-2,4-dion for Fmoc-chGtp(Boc_3)-OH; 1,2-cyclohexanedicarboxylic anhydride for Fmoc-Htp(Boc_3)-OH; 2,3-napthalic anhydride for Fmoc-Ntp(Boc_3)-OH; diglycolic anhydride for Fmoc-dGtp(Boc_3)-OH; glutaric anhydride for Fmoc-GEIPA(Boc_3)-OH; Boc-IDA anhydride for Fmoc-Boc-IDAtp(Boc_3)-OH; TFE-IDA anhydride for Fmoc-TFE-IDAtp(Boc_3)-OH) and N-(fluorenyl-9-methoxycarbonyloxy)-succinimide (Fmoc-OSu, 1.5 eq). Purification of the products was carried out by dry column vacuum chromatography (DCVC).

Bis-tfa-TEPA(Boc_3). Yield: 91 %. ^{1}H NMR (400 MHz, $CDCl_3$) δ (ppm) = 3.58 - 3.24 (m, 16H, C**H₂**-Tepa), 1.48 (s, 27H, C**H₃**-*tert*-but). ESI-MS: calc. for 681.3; $[M-H]^-$ found 680.3.

Bis-tfa-TETA(Boc_2). Yield: 84 %. ^{1}H NMR (400 MHz, $CDCl_3$) δ (ppm) = 3.70 - 3.26 (m, 12H, C$\mathbf{H_2}$-Teta), 1.47 (d, 18H, C$\mathbf{H_3}$-*tert*-but). ESI-MS: calc. for 538.2; $[M-H]^-$ found 537.2.

Bis-tfa-EIPA(Boc_2). Yield: 72 %. ^{1}H NMR (400 MHz, $CDCl_3$) δ (ppm) = 3.43 - 3.22 (m, 12H, C$\mathbf{H_2}$CH_2C$\mathbf{H_2}$-EIPA, C$\mathbf{H_2}$C$\mathbf{H_2}$-GEIPA), 1.76 (m, 4H, CH_2C$\mathbf{H_2}$CH_2-EIPA), 1.49 (s, 18H, C$\mathbf{H_3}$-*tert*-but). ESI-MS: calc. for 566.2; $[M-H]^-$ found 565.2.

Fmoc-Stp(Boc_3)-OH. Yield: 77 %. ^{1}H NMR (400 MHz, $CDCl_3$) δ (ppm) = 7.78 (d, 2H, Ar**H**-Fmoc), 7.61 (d, 2H, Ar**H**-Fmoc), 7.41 (t, 2H, Ar**H**-Fmoc), 7.31 (t, 2H, Ar**H**-Fmoc), 4.40 (m, 2H, C$\mathbf{H_2}$-Fmoc), 4.21 (m, 1H, C**H**-Fmoc), 3.47 - 3.20 (m, 16H, C$\mathbf{H_2}$-Tepa), 2.67 (t, 2H, C$\mathbf{H_2}$CONH-Suc), 2.48 (m, 2H, C$\mathbf{H_2}$COOH-Suc), 1.46 (s, 27H, C$\mathbf{H_3}$-*tert*-but). ESI-MS: calc. for 811.4; $[M-H]^-$ found 810.4.

Fmoc-Boc-IDAtp(Boc_3)-OH. Yield: 29 %. ^{1}H NMR (400 MHz, $CDCl_3$) δ (ppm) = 7.77 (d, 2H, Ar**H**-Fmoc), 7.59 (d, 2H, Ar**H**-Fmoc), 7.40 (t, 2H, Ar**H**-Fmoc), 7.31 (t, 2H, Ar**H**-Fmoc), 4.40 (m, 2H, C$\mathbf{H_2}$-Fmoc), 4.20 (m, 1H, C**H**-Fmoc), 3.91 (s, 4H, C$\mathbf{H_2}$-IDA) 3.48 - 3.19 (m, 16H, C$\mathbf{H_2}$-Tepa), 1.43 (s, 36H, C$\mathbf{H_3}$-*tert*-but). ESI-MS: calc. for 926.5; $[M-H]^-$ found 925.5.

Fmoc-dGtp(Boc$_3$)-OH. Yield: 73 %. ^{1}H NMR (400 MHz, CDCl$_3$) δ (ppm) = 7.77 (d, 2H, Ar**H**-Fmoc), 7.60 (d, 2H, Ar**H**-Fmoc), 7.42 (t, 2H, Ar**H**-Fmoc), 7.32 (t, 2H, Ar**H**-Fmoc), 4.41 (m, 2H, C**H$_2$**-Fmoc), 4.23 (m, 1H, C**H**-Fmoc), 4.17 (d, 2H, C**H$_2$**CONH-diglycolic acid), 4.10 (d, 2H, C**H$_2$**COOH- diglycolic acid), 1.45 (s, 27H, C**H$_3$**-*tert*-but). ESI-MS: calc. for 827.4; $[M-H]^-$ found 826.4.

Fmoc-Gtp(Boc$_3$)-OH. Yield: 68 %. ^{1}H NMR (400 MHz, CDCl$_3$) δ (ppm) = 7.79 (d, 2H, Ar**H**-Fmoc), 7.60 (d, 2H, Ar**H**-Fmoc), 7.41 (t, 2H, Ar**H**-Fmoc), 7.32 (t, 2H, Ar**H**-Fmoc), 4.41 (m, 2H, C**H$_2$**-Fmoc), 4.22 (m, 1H, C**H**-Fmoc), 3.48 - 3.24 (m, 16H, C**H$_2$**-Tepa), 2.42 (t, 2H, C**H$_2$**CONH-Glu), 2.29 (t, 2H, C**H$_2$**COOH-Glu), 1.97 (m, 2H, CH$_2$C**H$_2$**CH$_2$COOH-Glu), 1.47 (s, 27H, C**H$_3$**-*tert*-but). ESI-MS: calc. for 825.4; $[M-H]^-$ found 824.4.

Fmoc-Htp(Boc$_3$)-OH. Yield: 55 %. ^{1}H NMR (400 MHz, CDCl$_3$) δ (ppm) = 7.69 (d, 2H, Ar**H**-Fmoc), 7.51 (d, 2H, Ar**H**-Fmoc), 7.32 (t, 2H, Ar**H**-Fmoc), 7.23 (t, 2H, Ar**H**-Fmoc), 4.30 (m, 2H, C**H$_2$**-Fmoc), 4.12 (m, 1H, C**H**-Fmoc), 3.48 - 3.08 (m, 16H, C**H$_2$**-Tepa), 2.50 (d, 1H, C**H**CONH-Hexahydrophthalic acid), 2.16 (s, 1H, C**H**COOH-Hexahydrophthalic acid), 1.89 – 1.24 (m, 35H, C**H$_2$**-Hexahydrophthalic acid, C**H$_3$**-*tert*-but). ESI-MS: calc. for 865.5; $[M-H]^-$ found 864.5.

Fmoc-chGtp(Boc$_3$)-OH. Yield: 63 %. ^{1}H NMR (400 MHz, CDCl$_3$) δ (ppm) = 7.69 (d, 2H, Ar**H**-Fmoc), 7.52 (d, 2H, Ar**H**-Fmoc), 7.33 (t, 2H, Ar**H**-Fmoc), 7.24 (t, 2H, Ar**H**-Fmoc), 4.31 (m, 2H, C**H$_2$**-Fmoc), 4.14 (m, 1H, C**H**-Fmoc), 3.47 - 3.10 (m, 16H, C**H$_2$**-Tepa), 2.46 (s, 2H, C**H$_2$**CONH-Cyclohexanediacetic acid), 2.26 (m, 2H, C**H$_2$**COOH-Cyclohexanediacetic acid), 1.49 - 1.21 (m, 37H, C**H$_2$**- Cyclohexanediacetic acid, C**H$_3$**-*tert*-but). ESI-MS: calc. for 893.5; $[M-H]^-$ found 892.5.

Fmoc-TFE-IDAtp(Boc$_3$)-OH. Yield: 28 %. ^{1}H NMR (400 MHz, CDCl$_3$) δ (ppm) = 8.12 - 7.24 (m, 8H, Ar**H**-Fmoc), 4.36 (m, 2H, C**H$_2$**-Fmoc), 4.15 (t, 1H, C**H**-Fmoc), 3.71 - 3.02 (m, 22H, C**H$_2$**-Tepa, C**H$_2$**-TFE-IDA), 1.52 - 1.27 (m, 27H, C**H$_3$**-*tert*-but). ESI-MS: calc. for 908.4; $[M-H]^-$ found 907.4.

Fmoc-Gtt(Boc$_2$)-OH. Yield: 66 %. ^{1}H NMR (400 MHz, CDCl$_3$) δ (ppm) = 7.79 (d, 2H, Ar**H**-Fmoc), 7.61 (d, 2H, Ar**H**-Fmoc), 7.42 (t, 2H, Ar**H**-Fmoc), 7.32 (t, 2H, Ar**H**-Fmoc), 4.42 (m, 2H, C**H$_2$**-Fmoc), 4.22 (m, 1H, C**H**-Fmoc), 3.52 - 3.21 (m, 12H, C**H$_2$**-Teta), 2.41 (t, 2H, C**H$_2$**CONH-Glu), 2.28 (t, 2H, C**H$_2$**COOH-Glu), 1.98 (m, 2H, CH$_2$C**H$_2$**CH$_2$COOH-Glu), 1.45 (s, 18H, C**H$_3$**-*tert*-but). ESI-MS: calc. for 682.4; $[M-H]^-$ found 681.4.

Fmoc-GEIPA(Boc$_2$)-OH. Yield: 72 %. ^{1}H NMR (400 MHz, CDCl$_3$) δ (ppm) = 7.78 (d, 2H, Ar**H**-Fmoc), 7.62 (d, 2H, Ar**H**-Fmoc), 7.41 (t, 2H, Ar**H**-Fmoc), 7.32 (t, 2H, Ar**H**-Fmoc), 4.42 (m, 2H, C**H$_2$**-Fmoc), 4.23 (m, 1H, C**H**-Fmoc), 3.43 - 3.03 (m, 12H, C**H$_2$**CH$_2$C**H$_2$**-GEIPA, C**H$_2$**C**H$_2$**-GEIPA), 2.44 (t, 2H, C**H$_2$**CONH-Glu), 2.30 (t, 2H, C**H$_2$**COOH-Glu), 1.99 (m, 2H, CH$_2$C**H$_2$**CH$_2$COOH-Glu), 1.71 (m, 4H, CH$_2$C**H$_2$**CH$_2$-GEIPA), 1.47 (s, 18H, C**H$_3$**-*tert*-but). ESI-MS: calc. for 710.4; $[M-H]^-$ found 709.4.

Fmoc-Ptp(Boc$_3$)-OH. Yield: 45 %. ^{1}H NMR (400 MHz, CDCl$_3$) δ (ppm) = 7.81 - 7.25 (m, 12H, Ar**H**-Fmoc, Ar**H**-Phthalic acid), 4.41 (m, 2H, C**H$_2$**-Fmoc), 4.22 (m, 1H, C**H**-Fmoc), 3.69 - 3.17 (m, 16H, C**H$_2$**-Tepa), 1.44 (s, 27H, C**H$_3$**-*tert*-but). ESI-MS: calc. for 859.4; $[M-H]^-$ found 858.4.

Fmoc-Ntp(Boc$_3$)-OH. Yield: 52 %. ^{1}H NMR (400 MHz, CDCl$_3$) δ (ppm) = 7.98 - 7.17 (m, 14H, Ar**H**-Fmoc, Ar**H**-Naphthalenedicarboxylic acid), 4.29 (m, 2H, C**H$_2$**-Fmoc), 4.12 (m, 1H, C**H**-Fmoc), 3.62 - 3.12 (m, 16H, C**H$_2$**-Tepa), 1.34 (s, 27H, C**H$_3$**-*tert*-but). ESI-MS: calc. for 909.4; $[M-H]^-$ found 908.4.

Synthesis of hydroxystearic acid (OHSteA)

4 g oleic acid (14.16 mmol) were added into a 250 mL round-bottom flask. 60 mL TFA/DCM solution (v/v, 2/1) were added and the mixture was stirred at RT for 72 h. Subsequently, the solvent was removed by a nitrogen flow to yield 5.45 g of hydroxystearic acid TFA ester (13.74 mmol, 97 %). In the next step, 5.45 g hydroxystearic acid TFA ester (13.74 mmol) was dissolved in 60 mL NaOH methanol/water solution (v/v, 4/1, 1 M) and the mixture was stirred overnight. The next day 1 M HCl solution was added slowly to the mixture to adjust pH to 1 - 2. The mixture was centrifuged. The supernatant was evaporated to obtain light brown powder. The products were dissolved in H_2O and extracted 3 times with DCM. The organic phases were combined and evaporated to yield 3.57 g of hydroxystearic acid (11.9 mmol, 84 %) as white powder.

Hydroxystearic acid (OHSteA). Yield: 84 %. ^{1}H NMR (400 MHz, $CDCl_3$) δ (ppm) = 3.57 - 3.48 (q, 1H, C**H**-OH), 2.35 - 2.21 (t, 2H, C$\mathbf{H_2}$COOH), 1.63 - 1.49 (q, 2H, C$\mathbf{H_2}$$CH_2$COOH), 1.44 - 1.11 (m, 26H, -C$\mathbf{H_2}$-), 0.87 - 0.72 (t, 3H, C$\mathbf{H_3}$). ESI-MS: calc. for 300.3; $[M-H]^-$ found 299.3.

Solid-phase synthesis of xenopeptides

All xenopeptides were synthesized manually on 2-chlorotrityl chloride resin using standard Fmoc-based solid-phase peptide synthesis.[4] For standard Fmoc-α-amino acids, Fmoc-Stp(Boc_3)-OH, Fmoc-Boc-IDAtp(Boc_3)-OH, Fmoc-dGtp(Boc_3)-OH, Fmoc-Gtp(Boc_3)-OH, Fmoc-TFE-IDAtp(Boc_3)-OH, Fmoc-Gtt(Boc_2)-OH, or Fmoc-GEIPA(Boc_2)-OH, the coupling step was performed using 4 eq Fmoc-amino acid, 4 eq HOBt, 4 eq PyBOP, and 8 eq DIPEA in DCM/DMF (1/1, 5 mL g^{-1} resin) for 75 min. For Fmoc-Htp(Boc_3)-OH or Fmoc-chGtp(Boc_3)-OH, the coupling step was performed using 4 eq Fmoc-amino acid, 4 eq HOBt, 4 eq PyBOP, and 8 eq DIPEA in DCM/DMF (1/1, 5 mL g^{-1} resin) containing 1 % Triton X-100 overnight. For Fmoc-Ptp(Boc_3)-OH or Fmoc-Ntp(Boc_3)-OH, the coupling step was performed using 4 eq Fmoc-amino acid, 4 eq Oxyma, and 4 eq DIC in NMP (5 mL g^{-1} resin) overnight. Fmoc deprotection step was performed by 3 times incubation with 20 % piperidine in DMF (5 mL g^{-1} resin) containing 1 % Triton X-100 for 15 min. The resin was washed 3 times with DMF and 3 times with DCM after each coupling and deprotection step. A Kaiser test was then carried out. Briefly, 10-15 beads of the resin were transferred into a test tube. One to two drops each of the following solutions were added: 80 % (w/v) phenol in ethanol, 5 % (w/v) ninhydrin in ethanol and 20 µM KCN in pyridine. The mixture was incubated at 99°C for 5 min under steady shaking. The presence of free amines was indicated by a deep blue color. After the last deprotection of Fmoc-Lys(N_3)-OH, the N-terminal amino group was protected with 10 eq Boc_2O and 10 eq DIPEA in DCM/DMF (1/1, 5 mL g^{-1} resin). Dde deprotection of Fmoc-Lys(Dde)-OH was accomplished by 15 times incubation with 2 % hydrazine in DMF for 2 min. Subsequently, the resin was washed 5 times with DMF, 5 times with 10 % DIPEA in DMF, and 3 times with DCM (5 mL g^{-1} resin each). Afterwards, Fmoc-Lys(Fmoc)-OH was introduced followed by the coupling of different fatty acids. The resin was washed 3 times with DMF and DCM and dried in vacuo. The xenopeptides were cleaved off the resin by incubation with pre-cooled cleavage cocktail TFA/EDT/H_2O/TIS (94/2.5/2.5/1, 10 mL g^{-1} resin) for 30 min. The cleavage solution was then immediately precipitated in pre-cooled MTBE/*n*-hexane (1/1, 40 mL). Afterwards, the xenopeptides were purified by a Äkta purifier system (GE Healthcare Bio-Sciences AB, Uppsala, Sweden). The fractions were combined and snap frozen and lyophilized to obtain the final products.

$LogD_{7.4}$ determination of xenopeptides

Octanol-water partition coefficient D at pH 7.4 ($logD_{7.4}$) was determined as previously reported with slight modification.[5] Scheme S3 illustrates the $logD_{7.4}$ determination procedures of all xenopeptides in this study. Briefly, 10 µL of 10 mg/mL xenopeptides in water were diluted with 90 µL HEPES buffer (20 mM, pH 7.4) in 1.5 mL reaction tubes. 100 µL *n*-octanol were then added. The tubes were shaken at 1400 rpm for 24 h. Afterwards, the tubes were centrifuged followed by storage at 4 °C for 1 h to separate *n*-octanol and water phases. 70 µL of each phase were injected for HPLC analysis. The extinction at 280 nm was monitored and the peak area (A) of the xenopeptides was calculated. $LogD_{7.4}$ was calculated as $\log(A_{octanol} / A_{water})$. All experiments were performed in triplicate.

Preparation of Cas9 RNP and Cas9 RNP/ssDNA nanocarriers

Cas9 protein was expressed, purified, and stored at -80 °C in storage buffer (20 mM HEPES, 200 mM KCl, 10 mM $MgCl_2$, 1 mM DTT, 30 % glycerol) as described previously.[6] Cas9 protein and sgRNA (dissolved in RNase-free water) were mixed at 1:1 molar ratio and incubated at RT for 15 min to form the Cas9/sgRNA ribonucleoprotein (RNP) complexes. Afterwards, Cas9 RNP was added into the xenopeptide solution in HBG buffer (pH 7.4) at an N/P (nitrogen to phosphate of sgRNA) ratio of 24. The solution was mixed thoroughly by pipetting up and down and incubated at RT for another 15 min to generate the final Cas9 RNP nanocarriers. In case of preparing Cas9 RNP/ssDNA nanocarriers for HDR experiments, Cas9 RNP was mixed with ssDNA HDR template at 1:1 molar ratio and then added into the xenopeptide solution in HBG buffer (pH 7.4) at an N/P (nitrogen to phosphate of sgRNA plus ssDNA) ratio of 12. The solution was mixed and incubated for 40 min to generate the Cas9 RNP/ssDNA nanocarriers.

Characterization of Cas9 RNP nanocarriers

The hydrodynamic size, PDI and zeta potential of Cas9 RNP nanocarriers were measured by DLS using a Zetasizer Nano ZS (Malvern Instruments, UK). Cas9 RNP nanocarriers were prepared as described above. The hydrodynamic size of Cas9 RNP nanocarriers containing 1.25 µg Cas9 protein and 0.25 µg sgRNA (Cas9/sgRNA, 1:1 molar ratio) in 100 µL HBG was measured. Afterwards, 700 µL HEPES buffer (20 mM, pH 7.4) were added to each sample and the solution was mixed for the zeta potential measurement. For the dilution stability study, Cas9 RNP nanocarriers were prepared at an RNP concentration of 375 nM (6.25 µg Cas9 protein and 1.25 µg sgRNA) and then diluted with HBG buffer to a series of RNP concentrations (75, 50, 25, 10, 5, 2.5, 1, 0.5, and 0.1 nM). The size and zeta potential of diluted samples were measured as described above.

Heparin competition assay

Heparin competition assay was performed to evaluate the nanocarrier stability against anions. 50 µL TE buffer containing different amounts of Heparin were added to the 96-well plate. 50 µL of Cas9 RNP nanocarriers containing 250 ng Cas9 protein and 50 ng sgRNA were prepared and added to each well. The final amounts of Heparin were 0, 0.25, 0.5, 1, 2.5, 5 IU per µg of sgRNA. The plate was incubated at 37 °C for 30 min. Afterwards, 100 µL Ribogreen solution were added and a Ribogreen assay was performed as described above. HBG buffer and naked Cas9 RNP at the same conditions were prepared as the controls. After subtraction of the blank wells (HBG buffer), the fraction of dye exclusion was calculated as [1 − fraction of Ribogreen intercalation (normalized to Ribogreen intercalation of naked Cas9/sgRNA RNP)]. All experiments were performed in triplicate.

Nanocarrier stability in salt conditions

Nanocarrier stability against salt was studied using NaCl solutions. 50 µL TE buffer containing different concentrations of NaCl were added to the 96-well plate. 50 µL of Cas9 RNP nanocarriers containing 250 ng Cas9 protein and 50 ng sgRNA were prepared and added to each well. The final concentrations of NaCl were 0, 0.05, 0.15, 0.25, 0.5, 5 M. The plate was incubated at 37 °C for 30 min. Afterwards, 100 µL Ribogreen solution were added and a Ribogreen assay was performed. HBG buffer and naked Cas9 RNP at the same conditions were prepared as the controls. After subtraction of the blank wells (HBG buffer), the fraction of dye exclusion was calculated as [1 − fraction of Ribogreen intercalation (normalized to Ribogreen intercalation of naked Cas9/sgRNA RNP)]. All experiments were performed in triplicate.

Cellular uptake of Cas9 RNP nanocarriers

Cellular uptake of Cas9 RNP nanocarriers was evaluated by flow cytometry and confocal laser scanning microscopy (CLSM). ATTO647N-labeled Cas9 protein was prepared as described previously.[7] For flow cytometry experiments, HeLa WT cells were seeded into 24-well plates at a density of 25000 cells/well one day prior to the treatments. On the next day, the medium was replaced with 400 µL of fresh low-glucose DMEM medium (10 % FBS, 100 U/mL penicillin, and 100 µg/mL streptomycin). Cas9 RNP nanocarriers were prepared as described above using 20 % of ATTO647N-Cas9 and 20 % of ATTO488-sgRNA. 100 µL of RNP nanocarriers were added to each well resulting in a final concentration of 75 nM Cas9 RNP followed by incubation of the cells for 4 h. Afterwards, the medium was removed and 500 µL PBS containing 2000 IU heparin were added to disassociate the nanocarriers attached on the cell membrane. The cells were incubated on ice for 30 min. Subsequently, the cells were collected and prepared for flow cytometry analysis as described above. All experiments were performed in triplicate.

For CLSM experiments, HeLa WT cells were seeded in 8-well Ibidi µ-slides (Ibidi GmbH, Germany) at a density of 15000 cells/well one day prior to the treatments. On the next day, the medium was replaced with 240 µL of fresh medium. 60 µL of dye-labeled RNP nanocarriers were added to each well resulting in a final concentration of 75 nM Cas9 RNP followed by incubation of the cells for 4 h. The medium was removed and 300 µL PBS containing 1200 IU heparin were added. The cells were incubated on ice for 30 min. Subsequently, the cells were washed twice with PBS and fixed with 4 % PFA in PBS at RT for 40 min. The cells were then washed twice with PBS and the cell nuclei were stained with 2 µg/mL DAPI in PBS at RT for 20 min. Afterwards, the DAPI solution was removed and 300 µL fresh PBS were added to each well. The slides were stored at 4 °C in the dark until the measurement.

Endocytosis pathway study

Potential endocytosis pathways of Cas9 RNP nanocarriers with varied hydrophobicity were investigated by using different endocytosis inhibitors. One day prior to the treatments, HeLa WT cells were seeded into 24-well plates at a density of 25000 cells/well. On the next day, the medium was replaced with 500 µL of serum-free medium containing different endocytosis inhibitors (15.4 mM sodium azide, 10 µM chlorpromazine, 450 mM sucrose, 54 µM nystatin, or 1 mM amiloride). The cells were incubated at 37 °C for 2 h. Afterwards, the medium was replaced with 400 µL of fresh medium. 100 µL of dye-labeled RNP nanocarriers were added to each well resulting in a final concentration of 75 nM Cas9 RNP followed by incubation of the cells for 4 h. For 4 °C group, the cells were placed on ice for 2 h prior to the treatment of 75 nM Cas9 RNP in ice-cold medium. Afterwards, the medium was removed and 500 µL PBS containing 2000 IU heparin were added followed by incubation of the cells on ice for 30 min. The cells were then collected and prepared for flow cytometry analysis. All experiments were performed in triplicate.

Endosomal escape of Cas9 RNP nanocarriers

Endosomal escape of Cas9 RNP nanocarriers were studied by CLSM using a HeLa reporter cell line stably expressing mRuby3-galectin 8 fusion protein. The PB-CAG-mRuby3-Gal8-P2A-Zeo plasmid for generation of the reporter cell line was a gift from Jordan Green's lab (Addgene plasmid no. 150815; http://n2t.net/addgene:150815; RRID: Addgene_150815). HeLa mRuby3/gal8 cells were seeded in 8-well Ibidi µ-slides (Ibidi GmbH, Germany) at a density of 15000 cells/well one day prior to the treatments. On the next day, the medium was replaced with 240 µL of fresh medium. 60 µL of RNP nanocarriers were added to each well resulting in a final concentration of 75 nM Cas9 RNP followed by incubation of the cells for 4 h. Next, the cells were washed twice with PBS and then fixed with 4 % PFA at RT for 40 min. Afterwards, the cells were washed and incubated with 2 µg/mL DAPI (for staining cell nuclei) for 30 min. Then, the staining solution was removed and 300 µL fresh PBS were added. The slides were stored at 4 °C in the dark until the measurement. The number of mRuby3/gal8 spots per cell were analyzed using ImageJ software. In brief, cells, cell nuclei, and background were firstly distinguished using Color Threshold tool. Then, the image was converted into 8-bit type. The mRuby3/gal8 spots were further determined by Threshold tool, and the number of the spots in the cell cytosol per cell were counted by Analyze Particles tool.

eGFP knockout study

One day prior to the treatments, HeLa eGFP/tub cells were seeded into 96-well plates at a density of 5000 cells/well. On the next day, the medium was replaced with 80 µL of fresh medium. 20 µL of Cas9 RNP nanocarriers were added to each well resulting in a series of concentrations of RNP (100, 75, 50, 25, 10, 5, 2.5, 1, 0.5, and 0.1 nM). Lipofectamine CRISPRMAX was tested at the same concentrations of RNP with the preparation procedures as recommended by the manufacturer. The cells were incubated for 48 h. Afterwards, the cells were trypsinized and transferred to 24-well plates, and incubated for another 72 h. Then, the cells were harvested and prepared for flow cytometry analysis. All experiments were performed in triplicate.

eGFP to BFP conversion (HDR) study

One day prior to the treatments, HeLa GFPd2 cells were seeded into 96-well plates at a density of 5000 cells/well. On the next day, the medium was replaced with 80 µL of fresh medium. 20 µL of Cas9 RNP/ssDNA nanocarriers containing different molar ratios of RNP/ssDNA were added to each well resulting in a series of concentrations of RNP (100, 75, 50, 25, 10, 5, 2.5, 1, 0.5, and 0.1 nM). Lipofectamine CRISPRMAX was tested at the same concentrations of RNP and ssDNA with the preparation procedures as recommended by the manufacturer. The cells were incubated for 48 h. Afterwards, the cells were trypsinized and transferred to 24-well plates, and incubated for another 72 h. Then, the cells were harvested and prepared for flow cytometry analysis. All experiments were performed in triplicate.

Cell viability assay (MTT)

Treatments were performed as described in eGFP knockout and HDR studies. The cells were incubated for 48 h and an MTT assay was then carried out as mentioned above. All experiments were performed in triplicate.

Statistical analysis

Half maximal effective concentration (EC50) values were calculated by GraphPad prism 5. EC50-log$D_{7.4}$ correlation was analyzed with a second order polynomial equation by GraphPad prism 5. All other data were analyzed with GraphPad prism 5 and presented as arithmetic mean ± standard deviation (SD) of at least triplicates. The statistical significance of the experiments was estimated using the two-tailed student's t-test, **** $p \leqslant 0.0001$, *** $p \leq 0.001$, ** $p \leq 0.01$, * $p \leq 0.05$, ns: not significant.

Supporting Figures and Tables

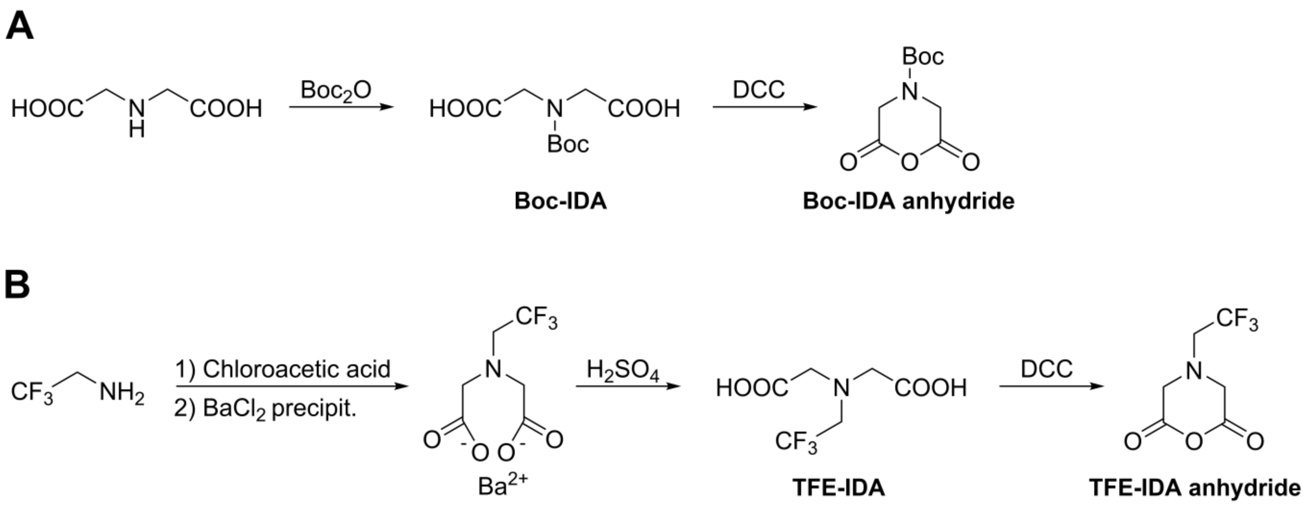

Scheme S1. Synthetic routes of Boc-IDA anhydride (A) and TFE-IDA anhydride (B).

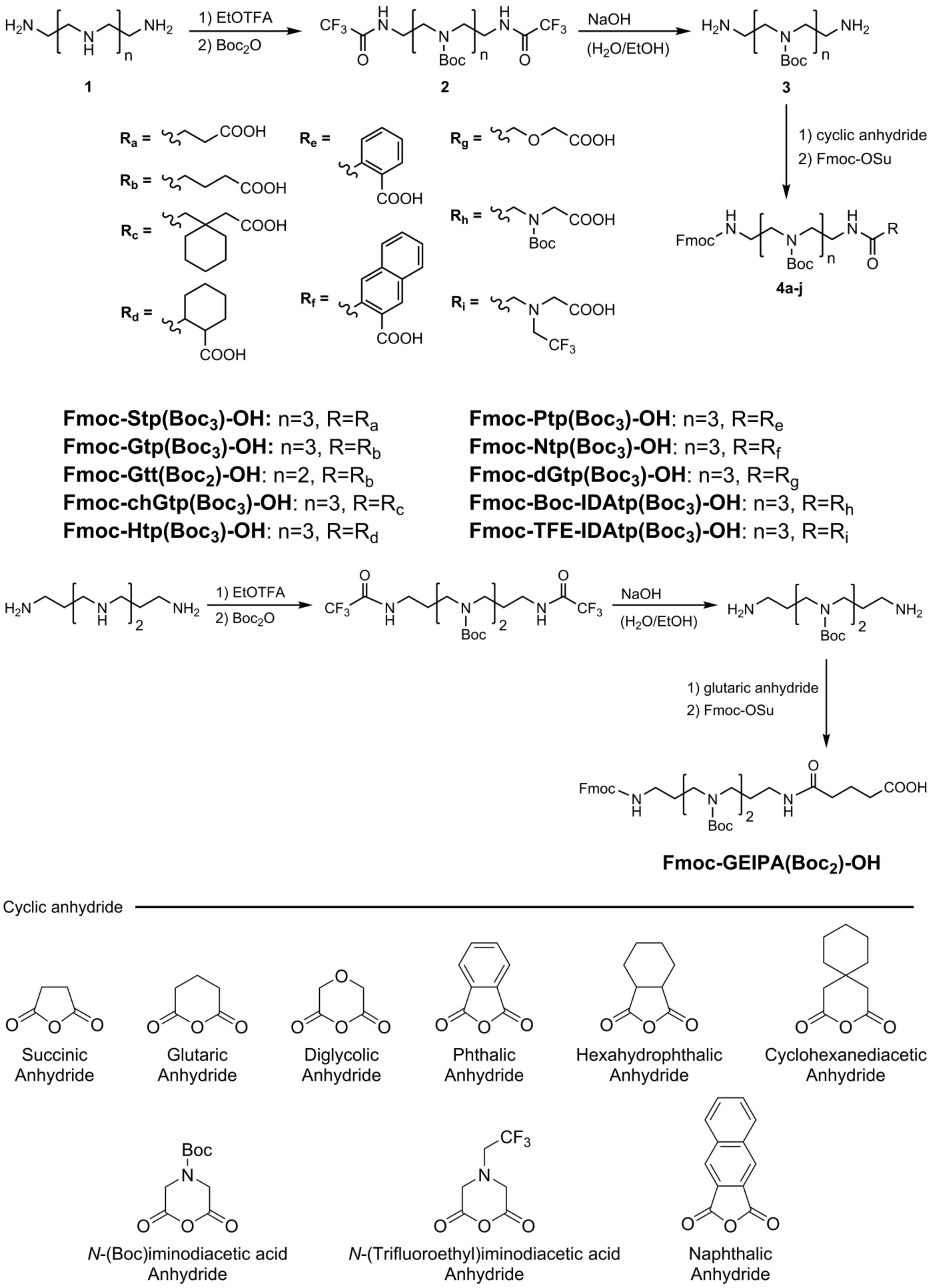

Scheme S2. A general synthetic route of artificial amino acid building blocks, and all cyclic anhydrides used in this study.

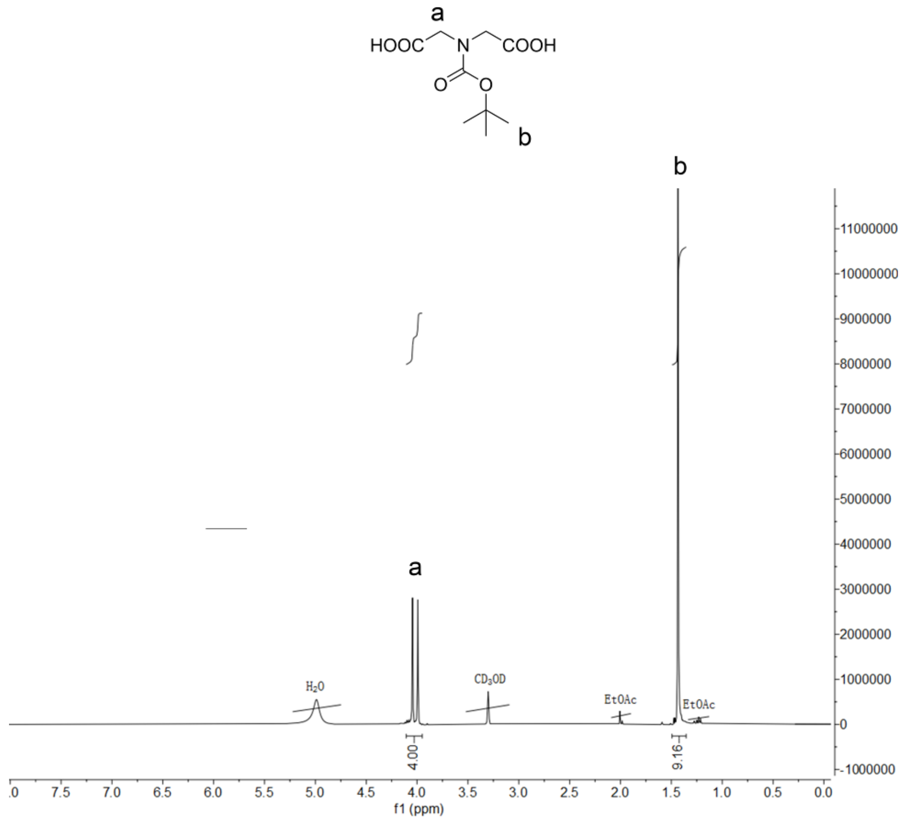

Figure S1. ^{1}H NMR spectrum of Boc-IDA in CD_3OD.

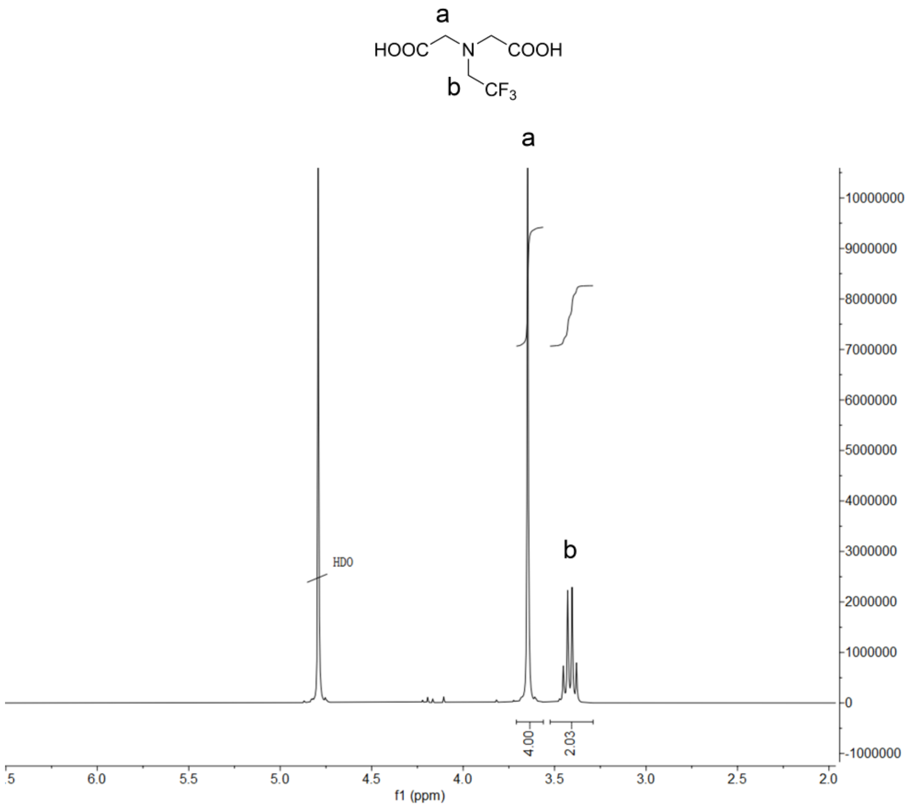

Figure S2. ^{1}H NMR spectrum of TFE-IDA in D_2O.

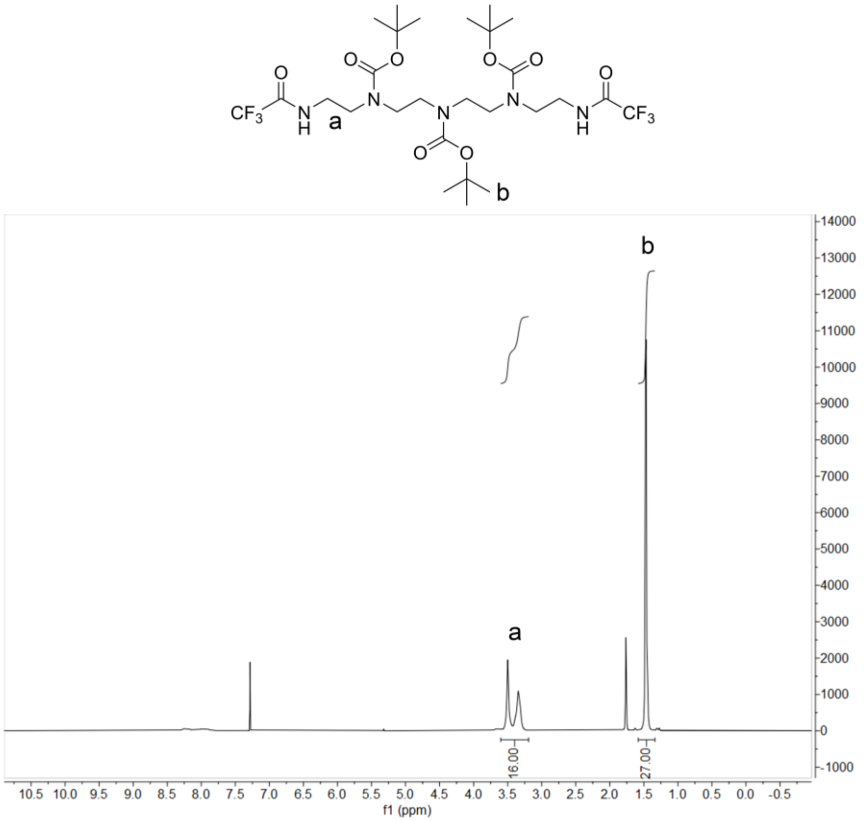

Figure S3. ^{1}H NMR spectrum of bis-tfa-TEPA(Boc_3) in $CDCl_3$.

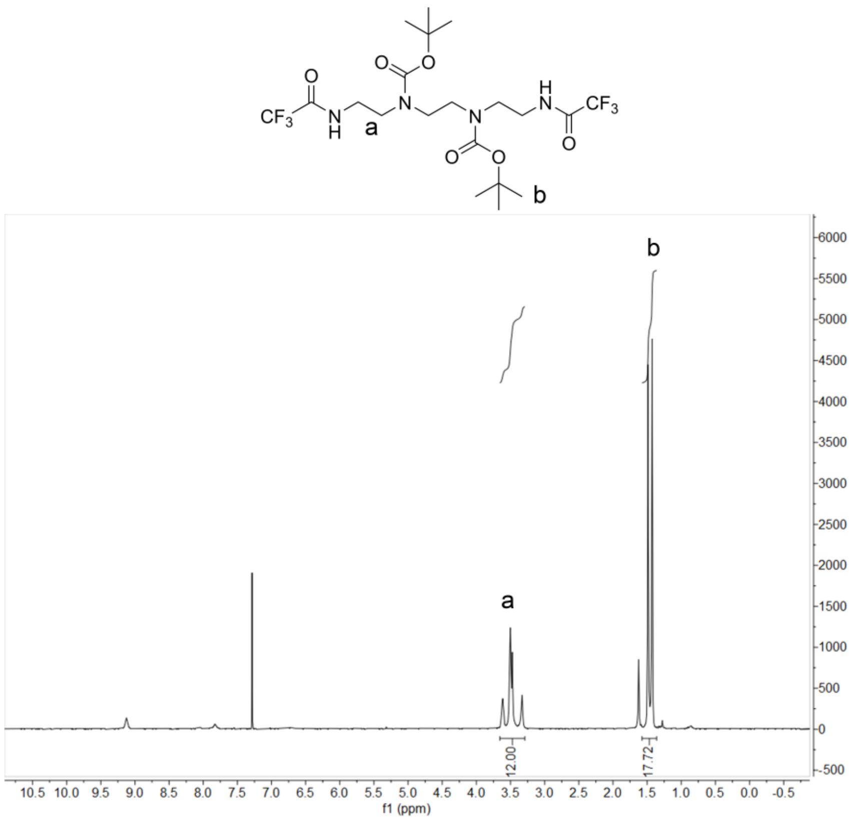

Figure S4. ^{1}H NMR spectrum of bis-tfa-TETA(Boc_2) in $CDCl_3$.

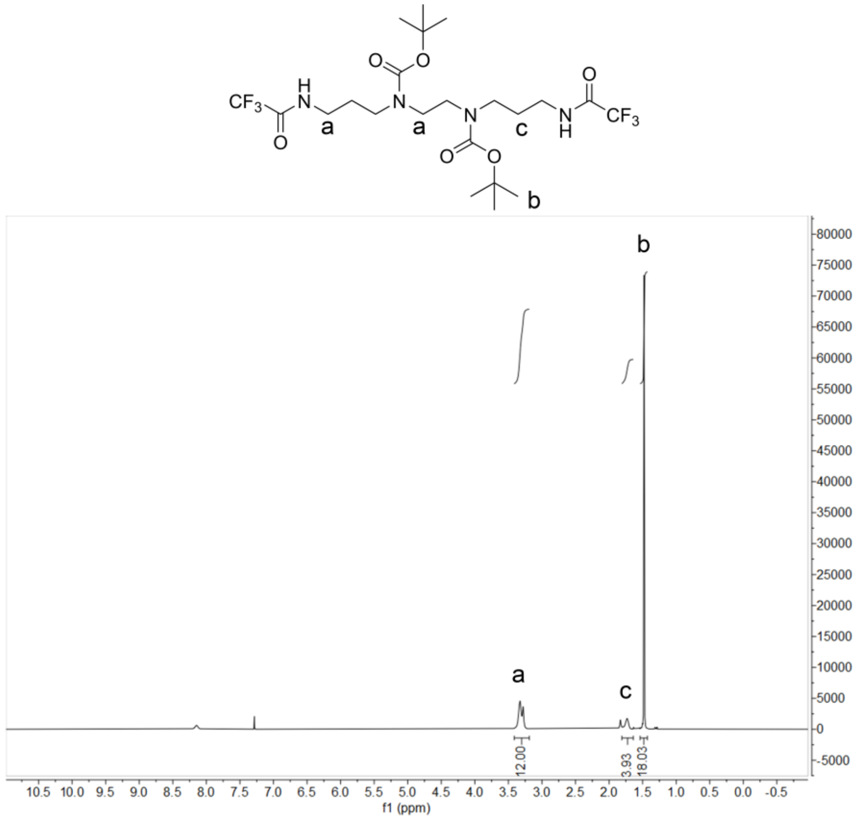

Figure S5. ^{1}H NMR spectrum of bis-tfa-EIPA(Boc$_2$) in CDCl$_3$.

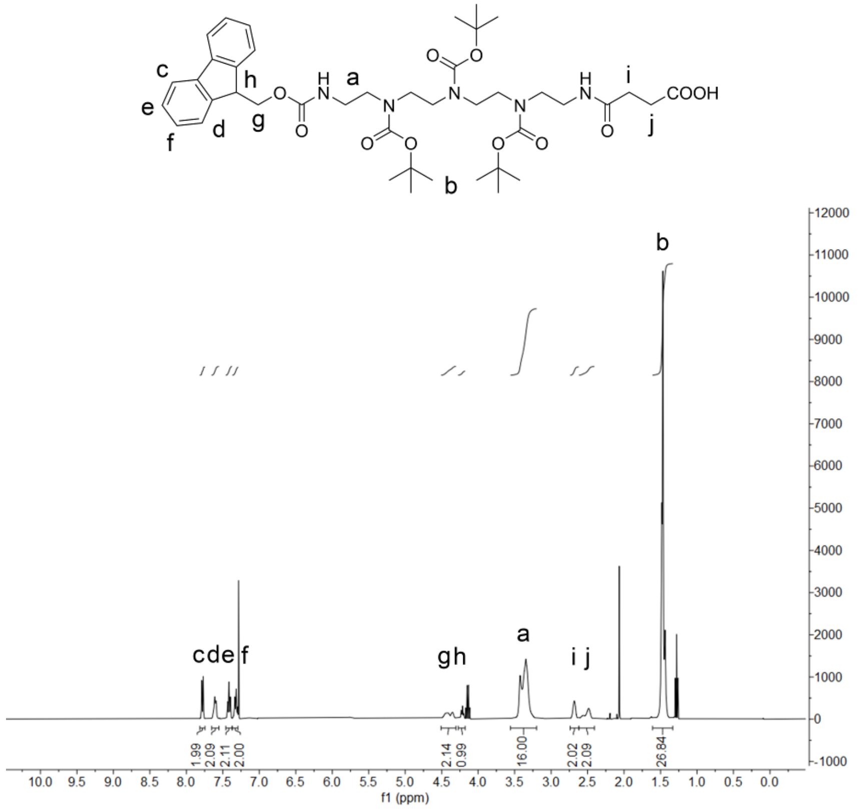

Figure S6. ^{1}H NMR spectrum of Fmoc-Stp(Boc$_3$)-OH in CDCl$_3$.

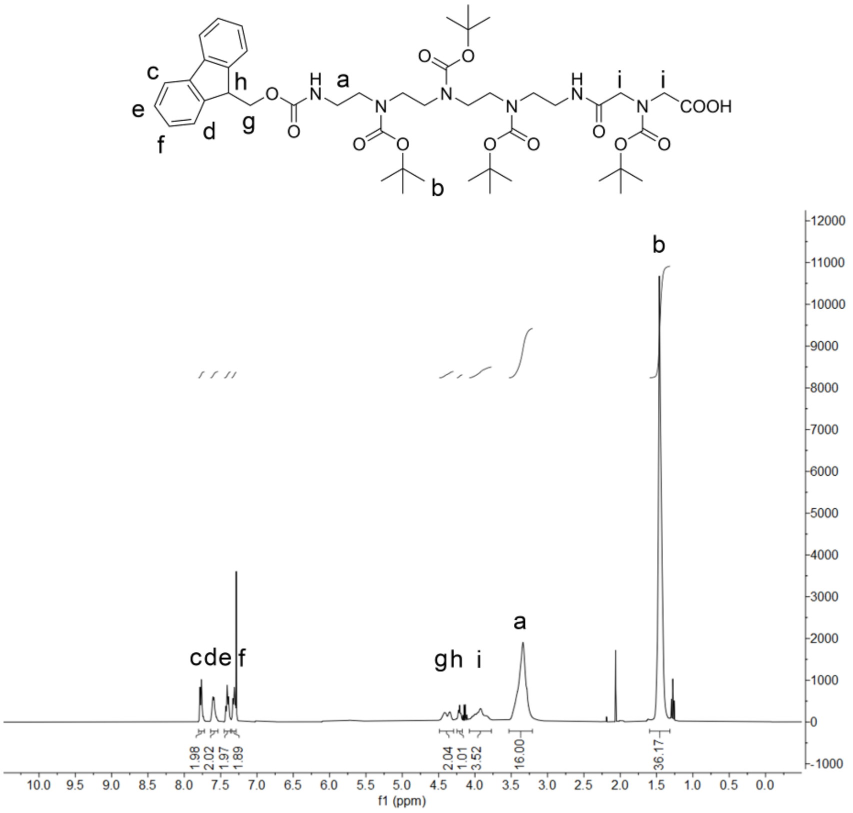

Figure S7. ^{1}H NMR spectrum of Fmoc-Boc-IDAtp(Boc$_3$)-OH in $CDCl_3$.

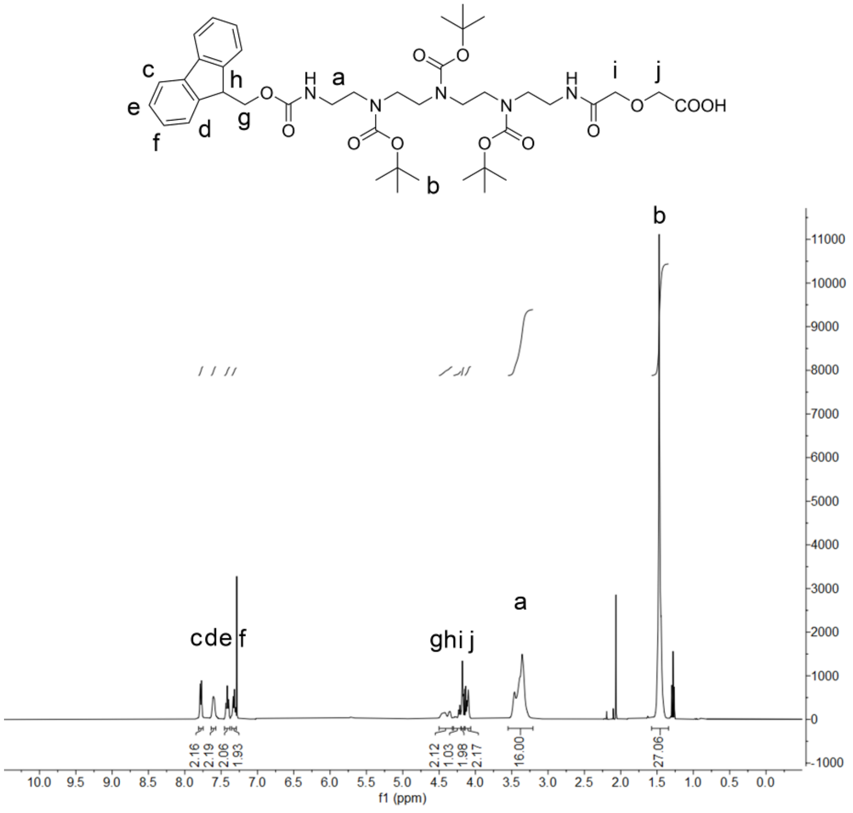

Figure S8. ^{1}H NMR spectrum of Fmoc-dGtp(Boc$_3$)-OH in $CDCl_3$.

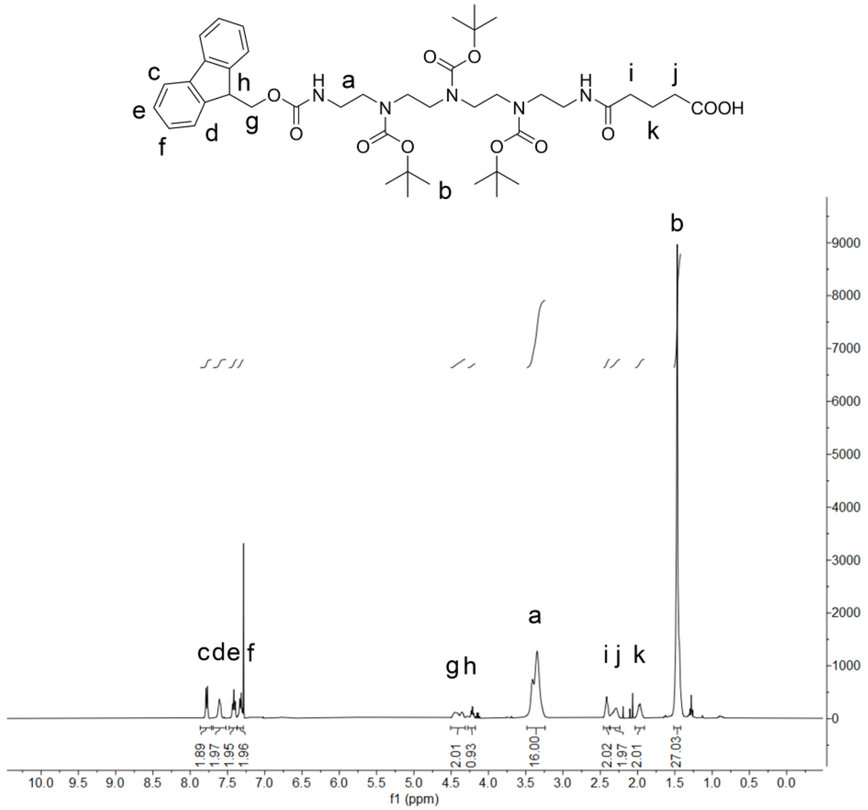

Figure S9. ${}^{1}H$ NMR spectrum of Fmoc-Gtp(Boc$_3$)-OH in $CDCl_3$.

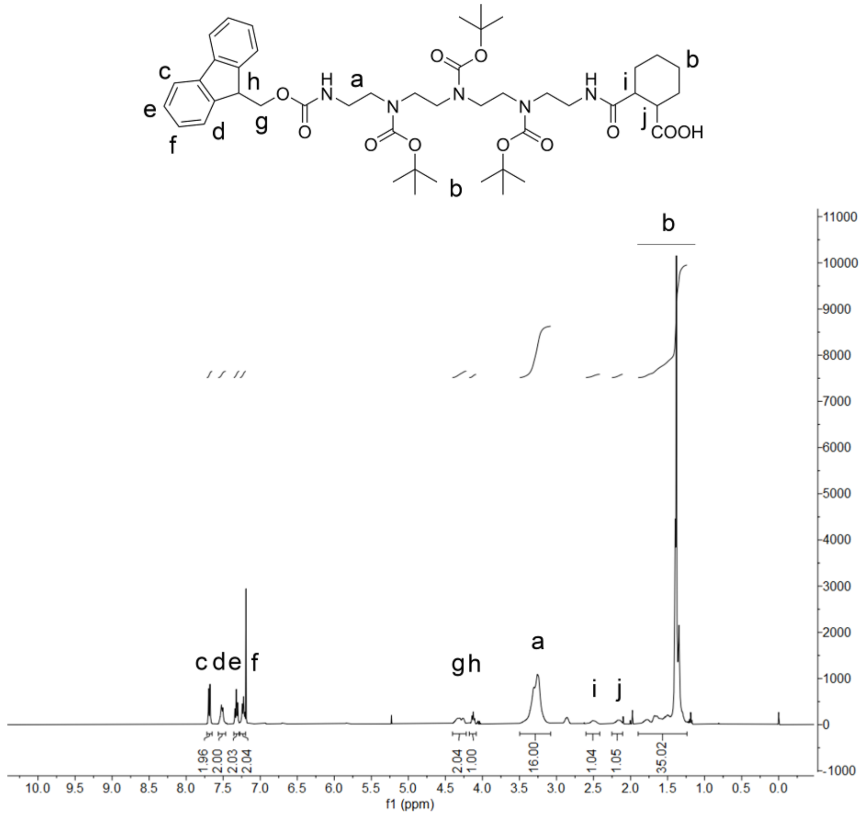

Figure S10. ${}^{1}H$ NMR spectrum of Fmoc-Htp(Boc$_3$)-OH in $CDCl_3$.

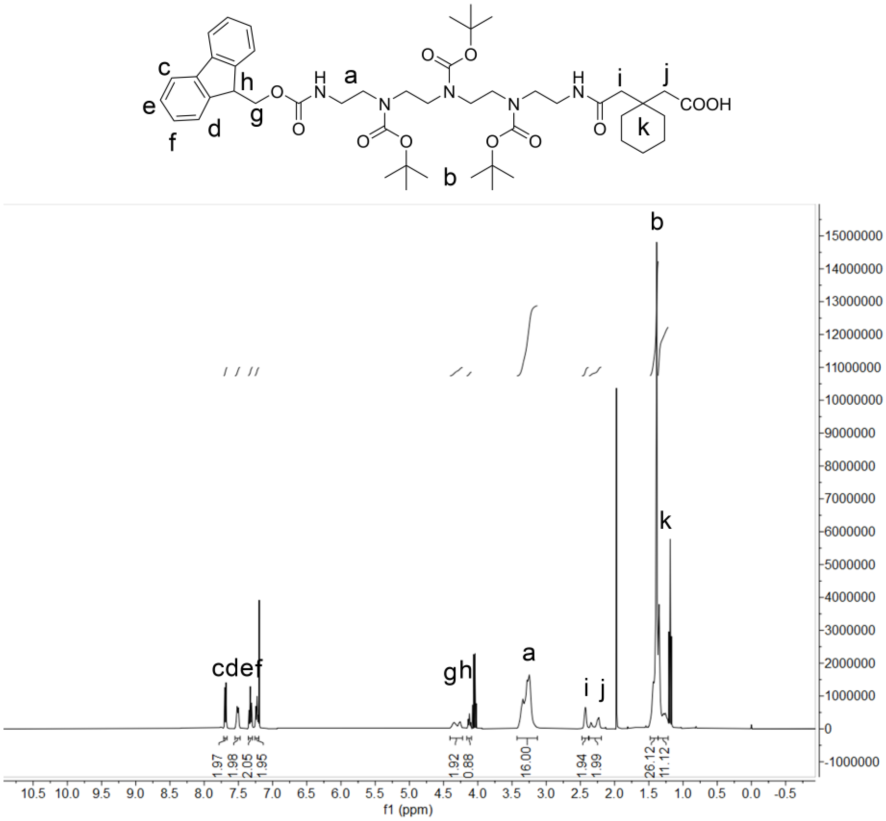

Figure S11. ^{1}H NMR spectrum of Fmoc-chGtp(Boc_3)-OH in $CDCl_3$.

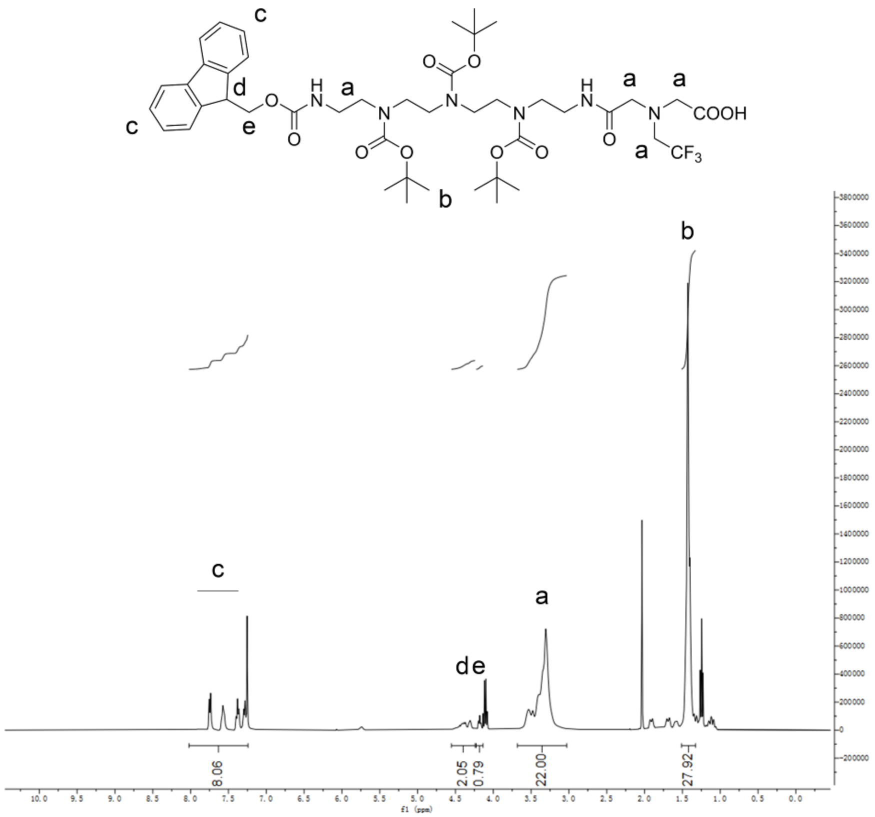

Figure S12. ^{1}H NMR spectrum of Fmoc-TFE-IDAtp(Boc_3)-OH in $CDCl_3$.

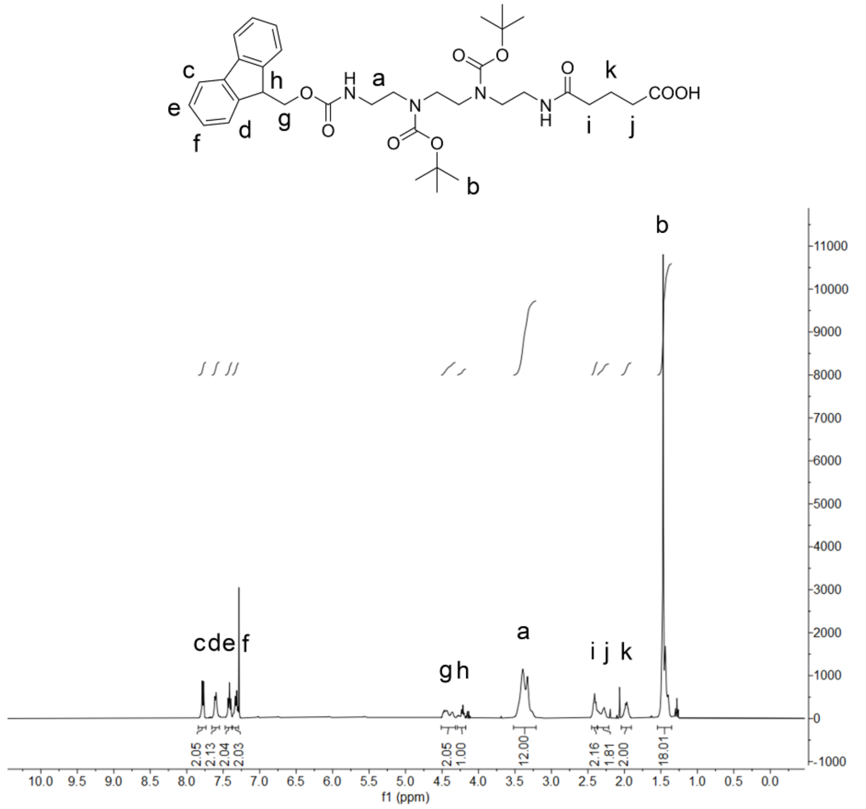

Figure S13. ^{1}H NMR spectrum of Fmoc-Gtt(Boc$_2$)-OH in $CDCl_3$.

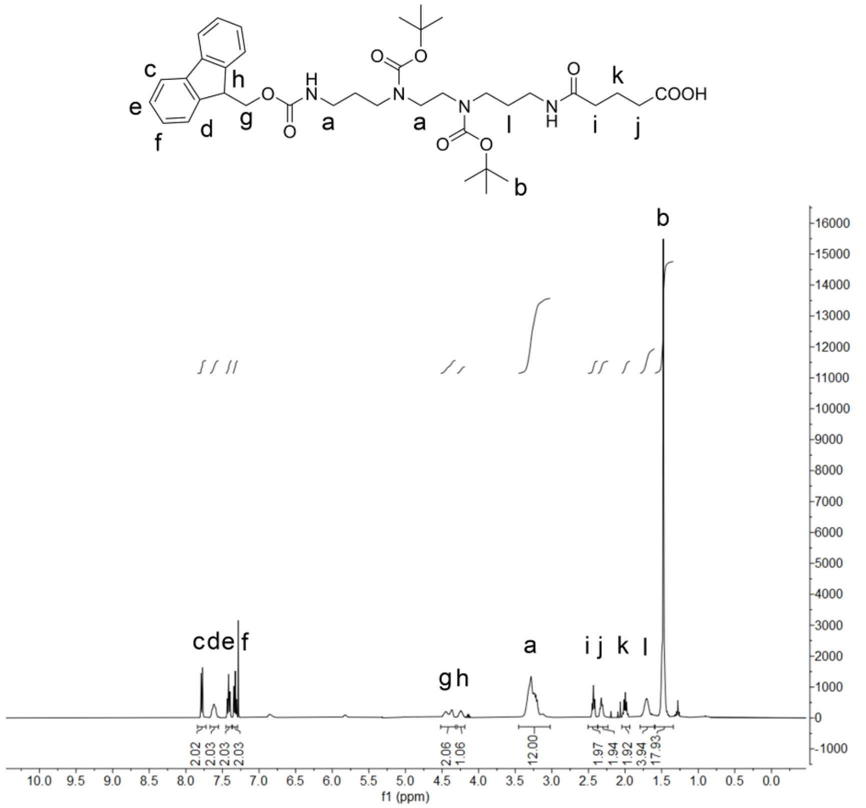

Figure S14. ^{1}H NMR spectrum of Fmoc-GEIPA(Boc$_2$)-OH in $CDCl_3$.

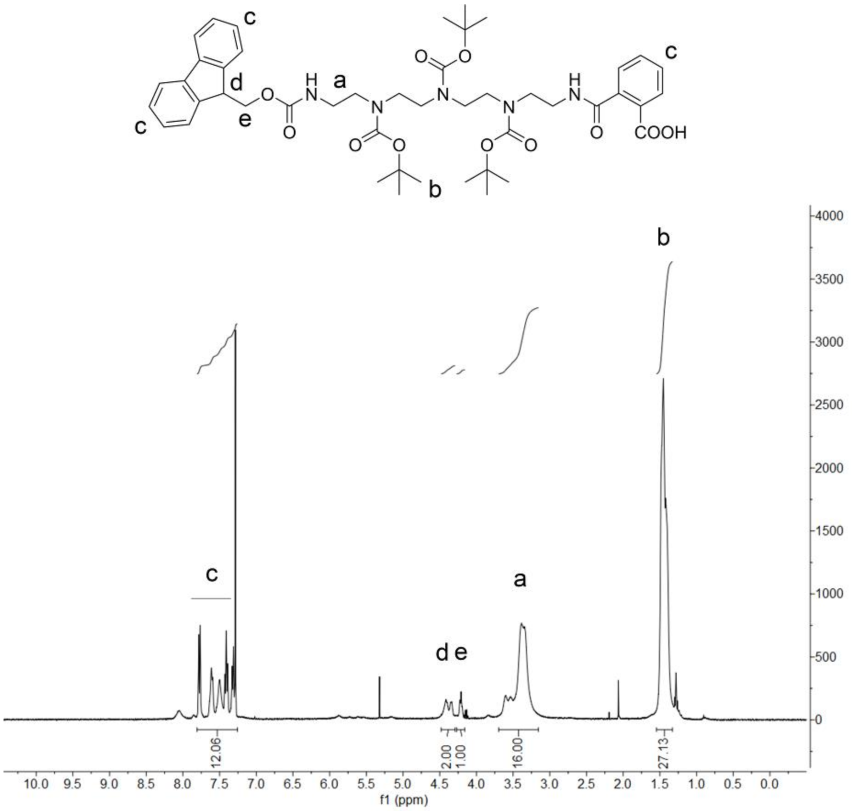

Figure S15. ^{1}H NMR spectrum of Fmoc-Ptp(Boc$_3$)-OH in $CDCl_3$.

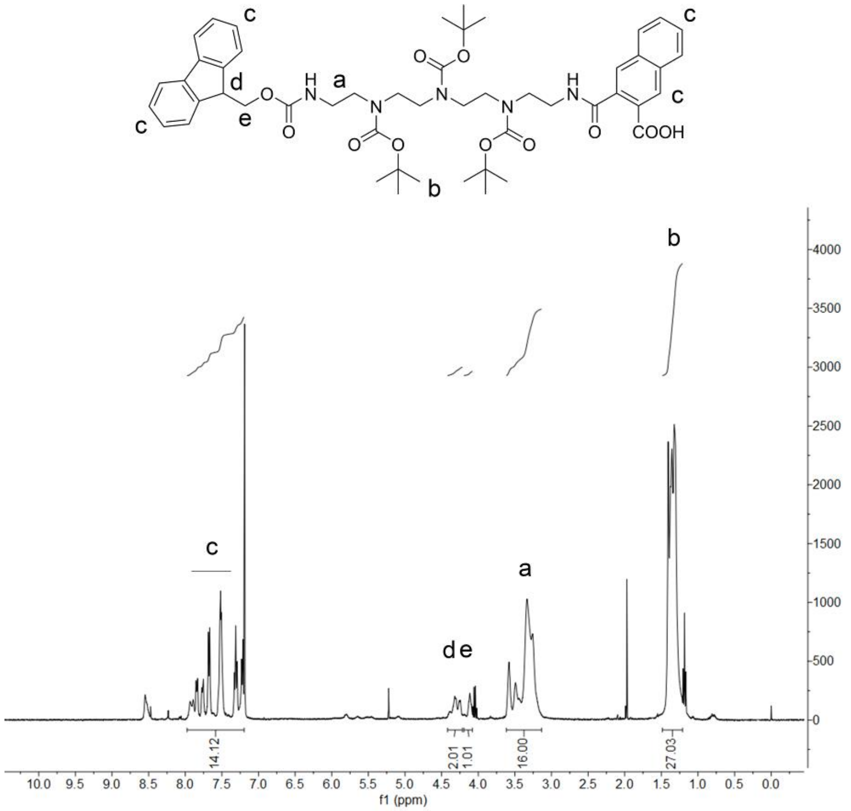

Figure S16. ^{1}H NMR spectrum of Fmoc-Ntp(Boc$_3$)-OH in $CDCl_3$.

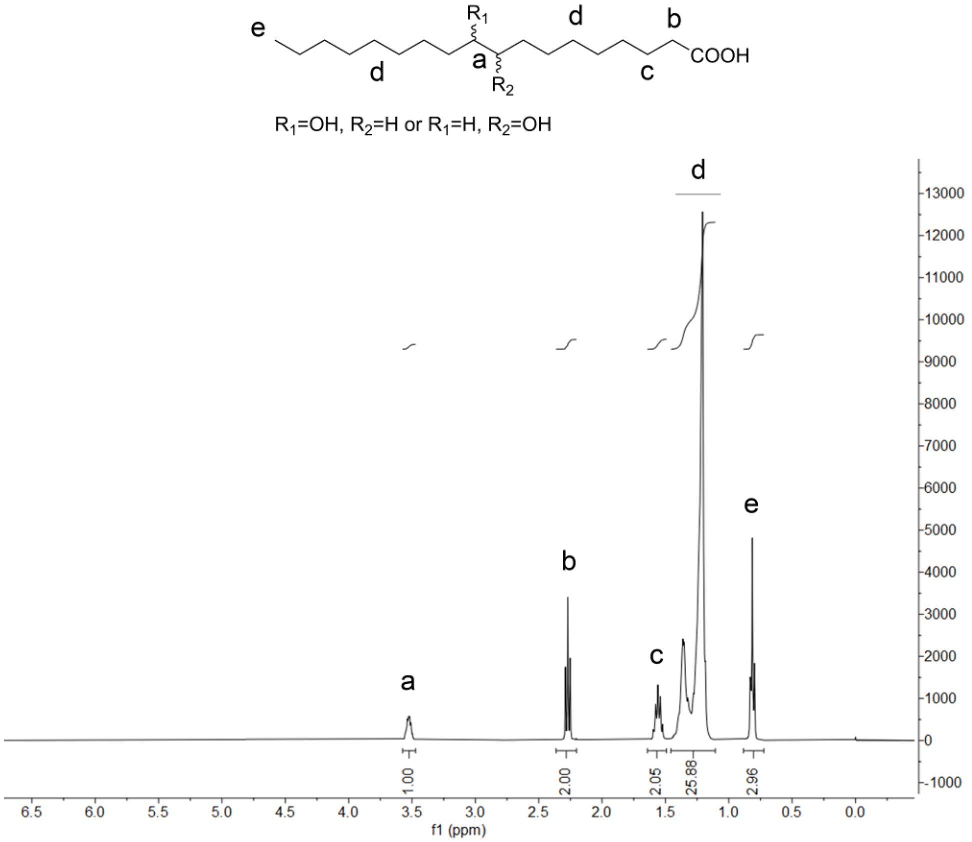

Figure S17. ^{1}H NMR spectrum of hydroxylstearic acid (OHSteA) in $CDCl_3$.

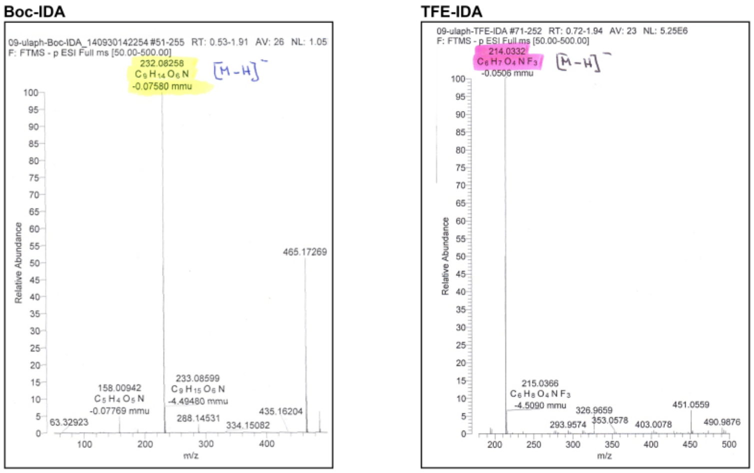

	Exact mass	Found mass [M-H]-
Boc-IDA	233.1	232.1
TFE-IDA	215.0	214.0

Figure S18. ESI-MS spectra of Boc-IDA and TFE-IDA.

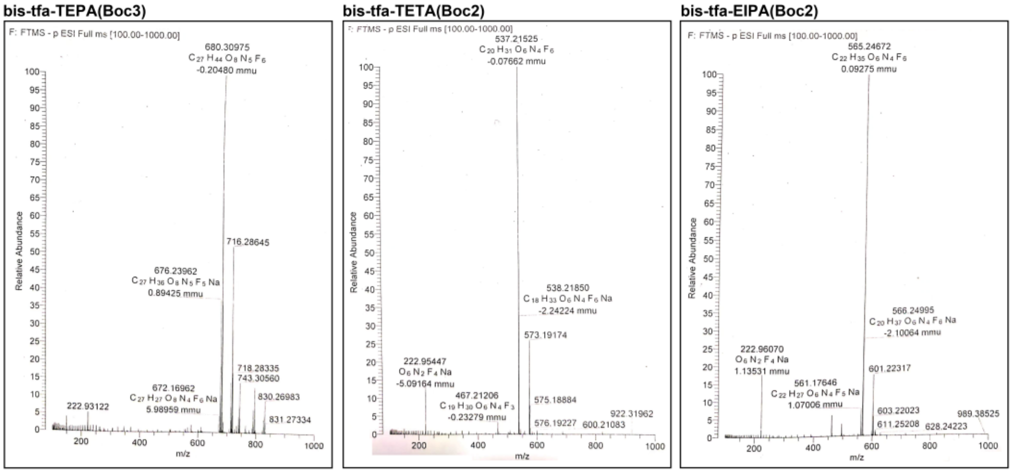

	Exact mass	Found mass [M-H]-
bis-tfa-TEPA(Boc3)	681.3	680.3
bis-tfa-TETA(Boc2)	538.2	537.2
bis-tfa-EIPA(Boc2)	566.2	565.2

Figure S19. ESI-MS spectra of bis-tfa-TEPA(Boc_3), bis-tfa-TETA(Boc_2), and bis-tfa-EIPA(Boc_2).

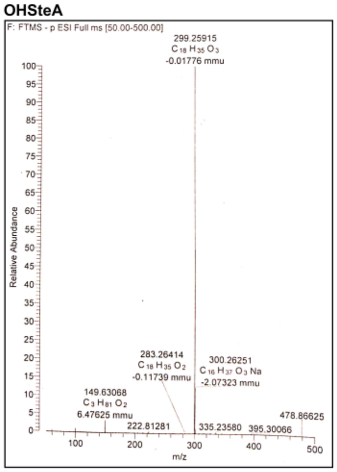

	Exact mass	Found mass [M-H]-
OHSteA	300.3	299.3

Figure S20. ESI-MS spectrum of hydroxylstearic acid (OHSteA).

Fmoc-Stp(Boc3)-OH

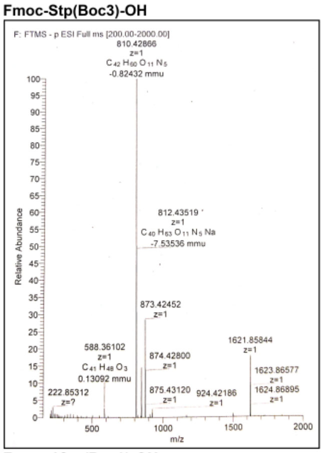

Fmoc-Boc-IDAtp(Boc3)-OH

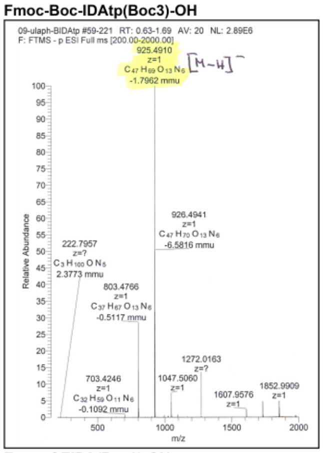

Fmoc-chGtp(Boc3)-OH

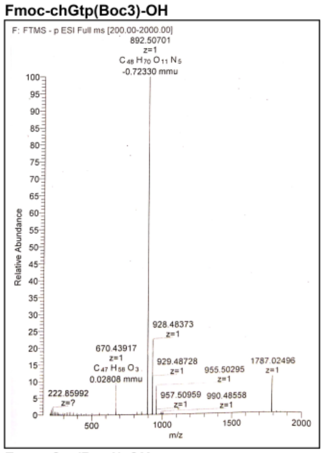

Fmoc-dGtp(Boc3)-OH

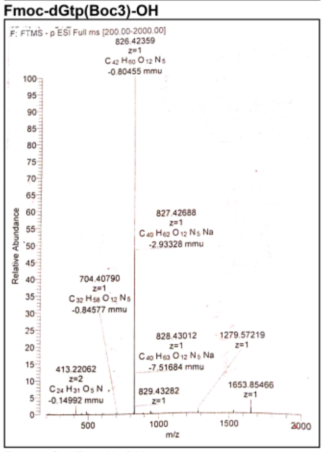

Fmoc-GEIPA(Boc2)-OH

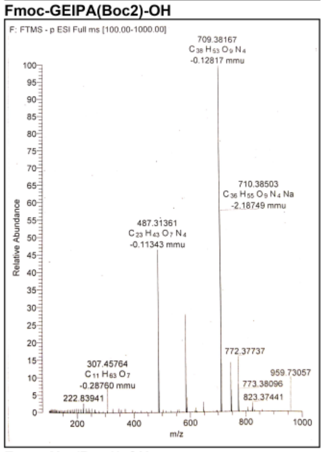

Fmoc-Gtp(Boc3)-OH

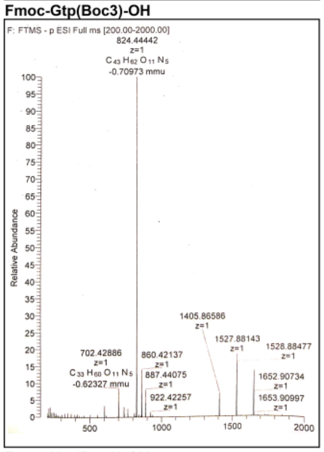

Fmoc-Gtt(Boc2)-OH

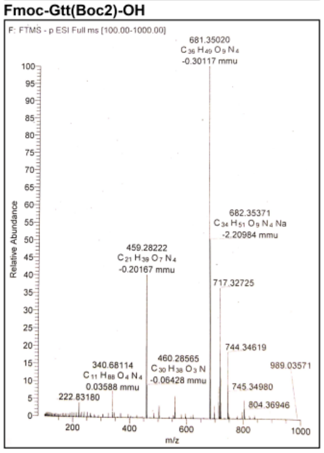

Fmoc-Htp(Boc3)-OH

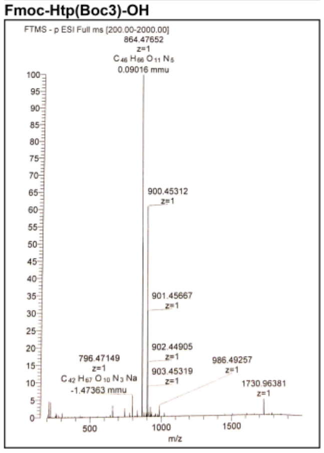

Fmoc-Ntp(Boc3)-OH

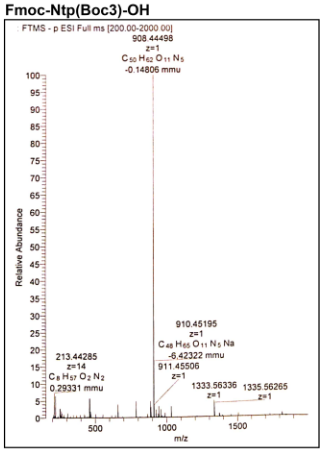

Fmoc-Ptp(Boc3)-OH

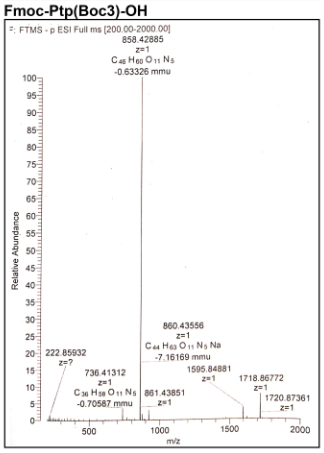

Fmoc-TFE-IDAtp(Boc3)-OH

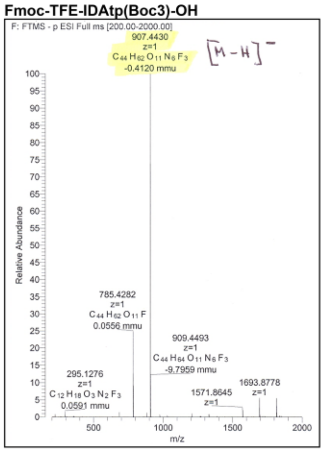

Building block	Exact mass	Found mass [M-H]-
Fmoc-Stp(Boc3)-OH	811.4	810.4
Fmoc-Boc-IDAtp(Boc3)-OH	926.5	925.5
Fmoc-chGtp(Boc3)-OH	893.5	892.5
Fmoc-dGtp(Boc3)-OH	827.4	826.4
Fmoc-GEIPA(Boc2)-OH	710.4	709.4
Fmoc-Gtp(Boc3)-OH	825.4	824.4
Fmoc-Gtt(Boc2)-OH	682.4	681.4
Fmoc-Htp(Boc3)-OH	865.5	864.5
Fmoc-Ntp(Boc3)-OH	909.4	908.4
Fmoc-Ptp(Boc3)-OH	859.4	858.4
Fmoc-TFE-IDAtp(Boc3)-OH	908.4	907.4

Figure S21. ESI-MS spectra of all artificial amino acid building blocks.

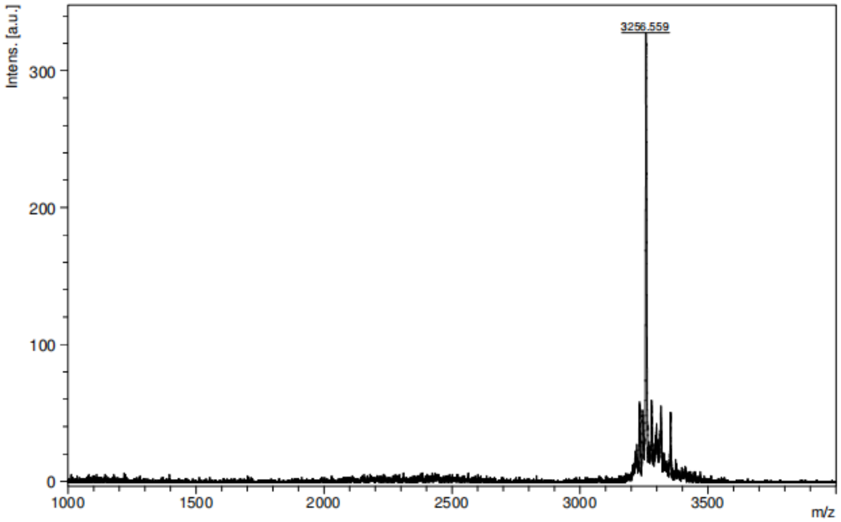

Figure S22. MALDI-TOF mass spectrum of Stp2-C-OHSteA, found mass: 3256.559.

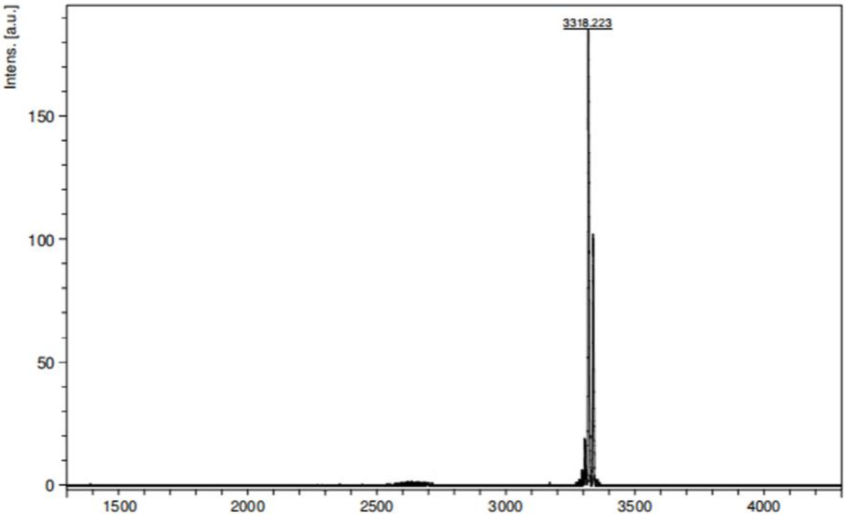

Figure S23. MALDI-TOF mass spectrum of IDAtp2-C-OHSteA, found mass: 3318.223.

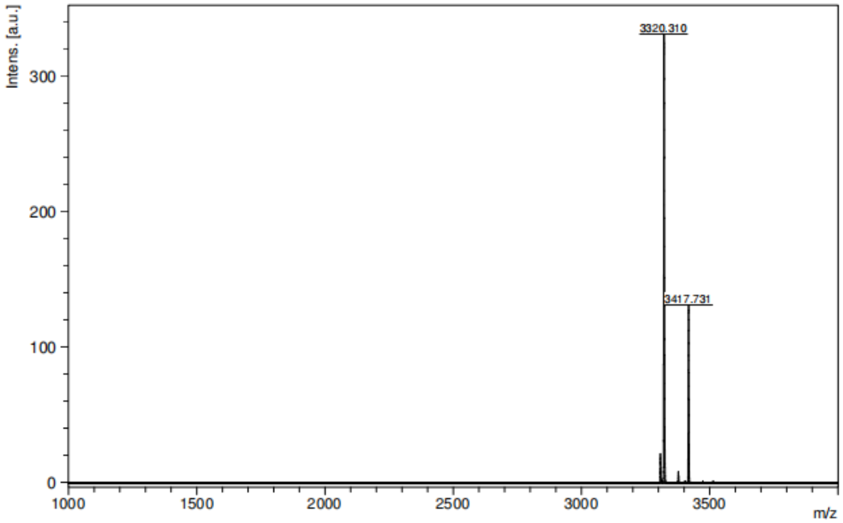

Figure S24. MALDI-TOF mass spectrum of dGtp2-C-OHSteA, found mass: 3320.310.

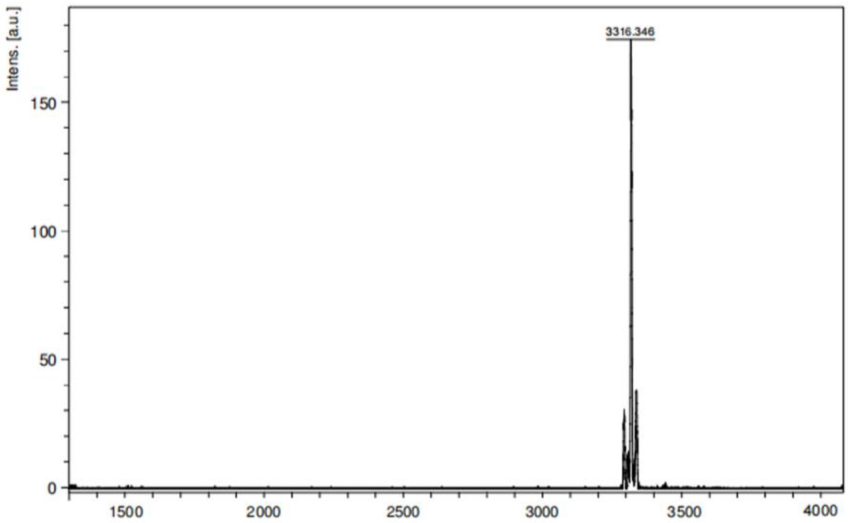

Figure S25. MALDI-TOF mass spectrum of Gtp2-C-OHSteA, found mass: 3316.346.

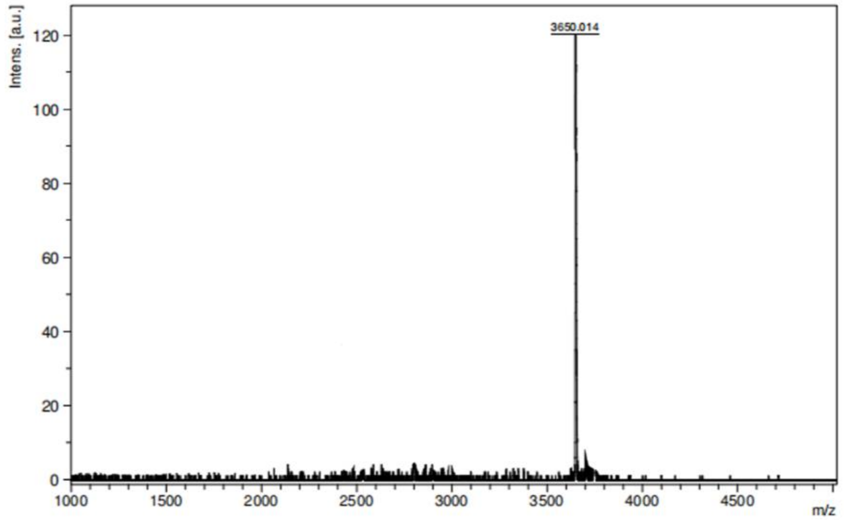

Figure S26. MALDI-TOF mass spectrum of TFE-IDAtp2-C-OHSteA, found mass: 3650.014.

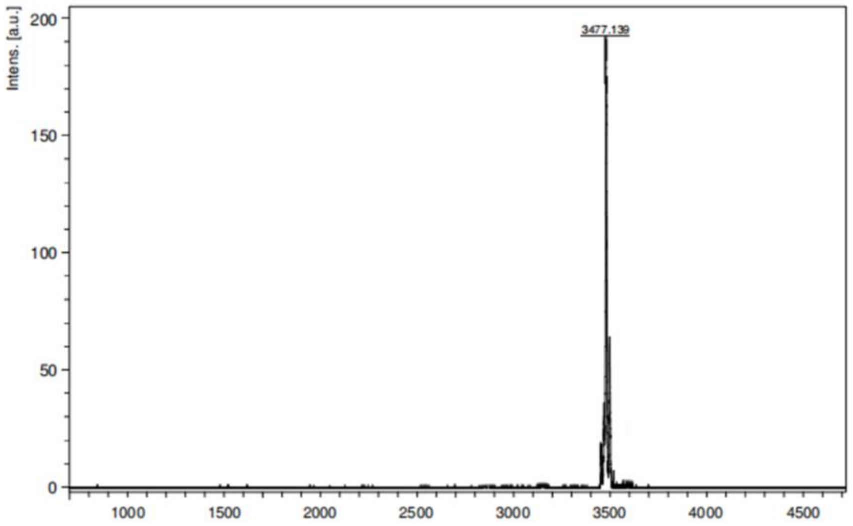

Figure S27. MALDI-TOF mass spectrum of Htp2-C-OHSteA, found mass: 3477.139.

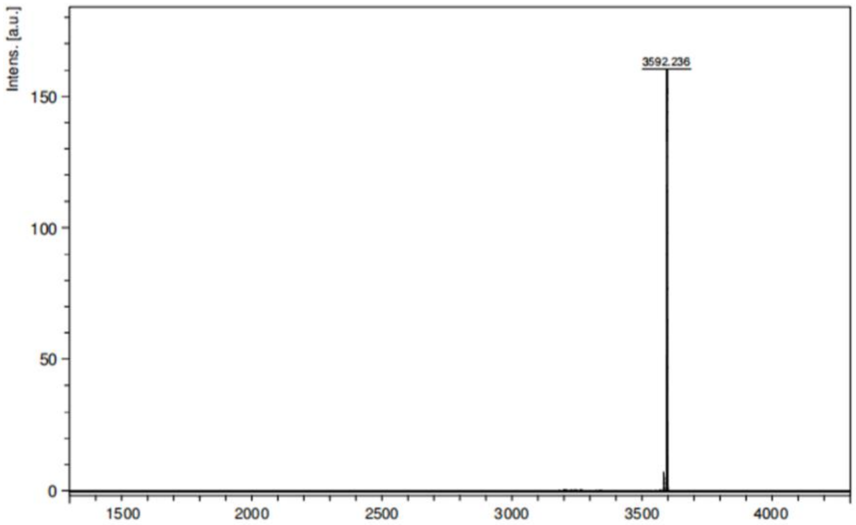

Figure S28. MALDI-TOF mass spectrum of chGtp2-C-OHSteA, found mass: 3592.236.

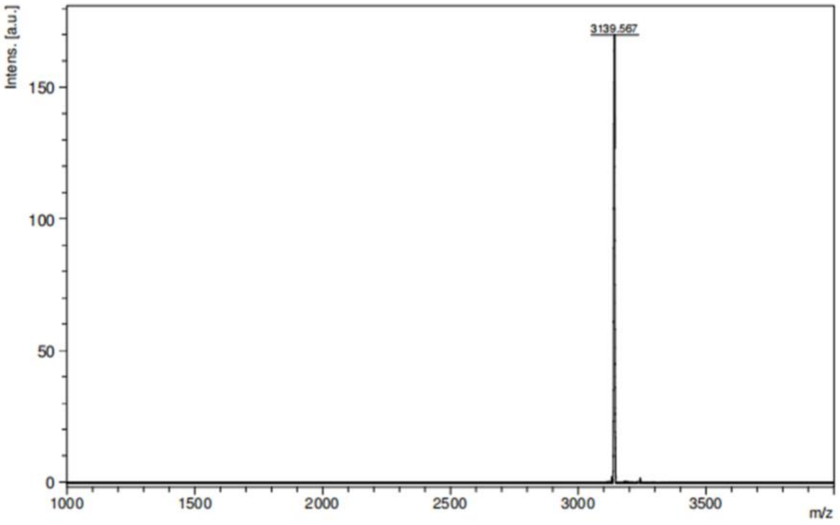

Figure S29. MALDI-TOF mass spectrum of Gtt2-C-OHSteA, found mass: 3139.567.

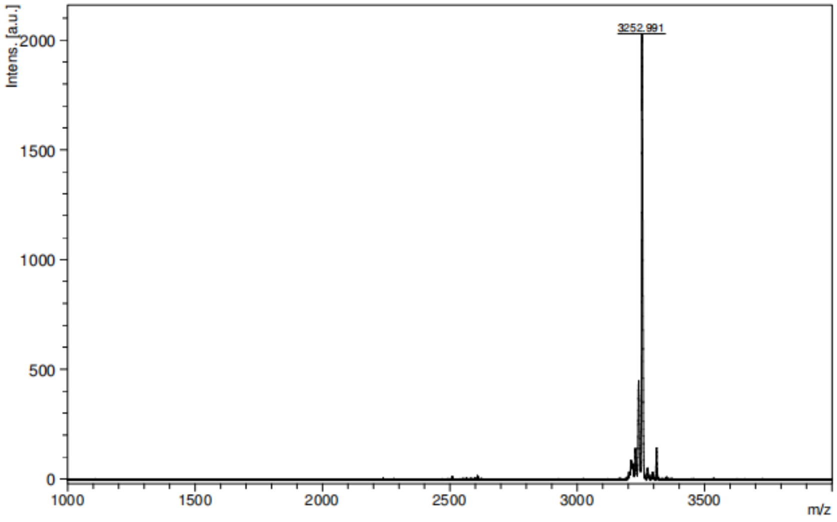

Figure S30. MALDI-TOF mass spectrum of GEIPA2-C-OHSteA, found mass: 3252.991.

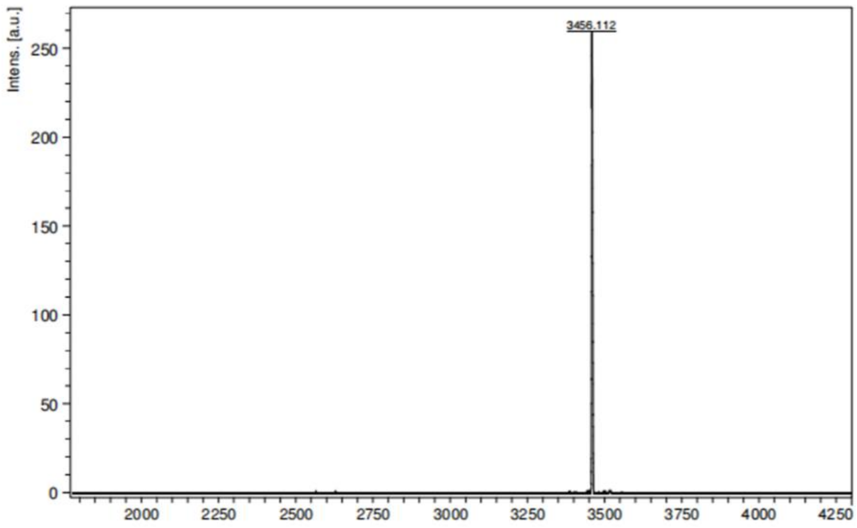

Figure S31. MALDI-TOF mass spectrum of Ptp2-C-OHSteA, found mass: 3456.112.

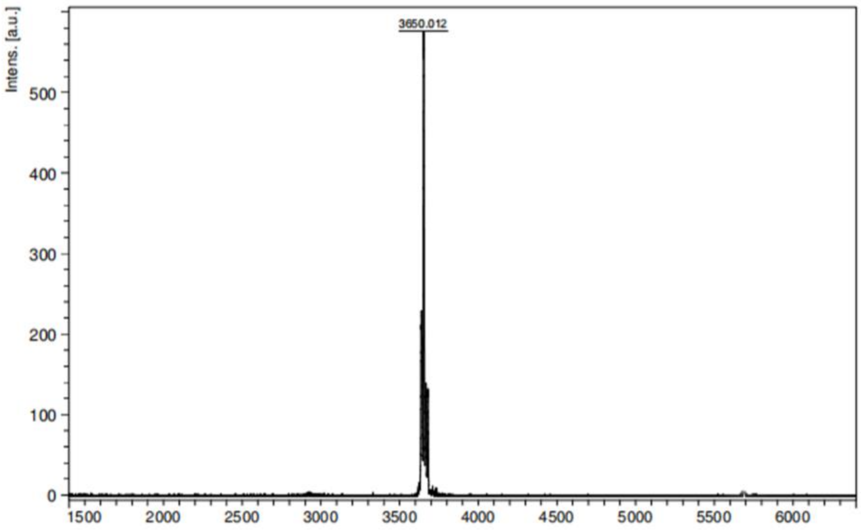

Figure S32. MALDI-TOF mass spectrum of Ntp2-C-OHSteA, found mass: 3650.012.

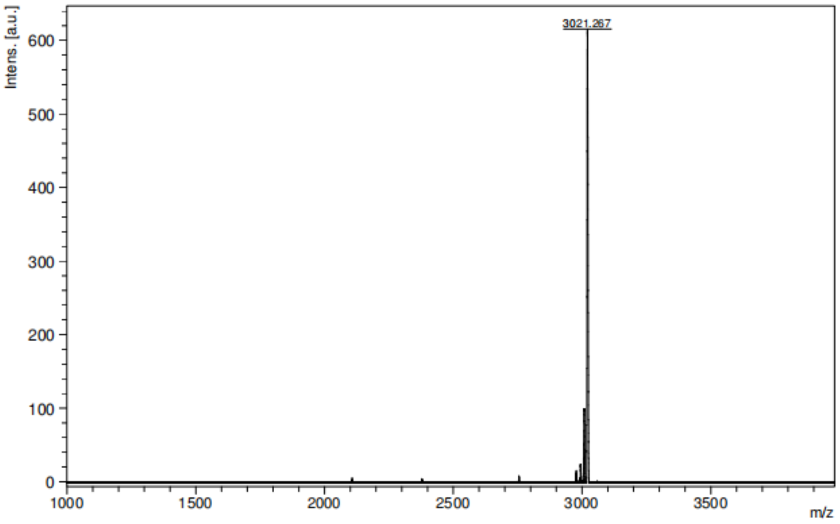

Figure S33. MALDI-TOF mass spectrum of Stp1-H-LinA, found mass: 3021.267.

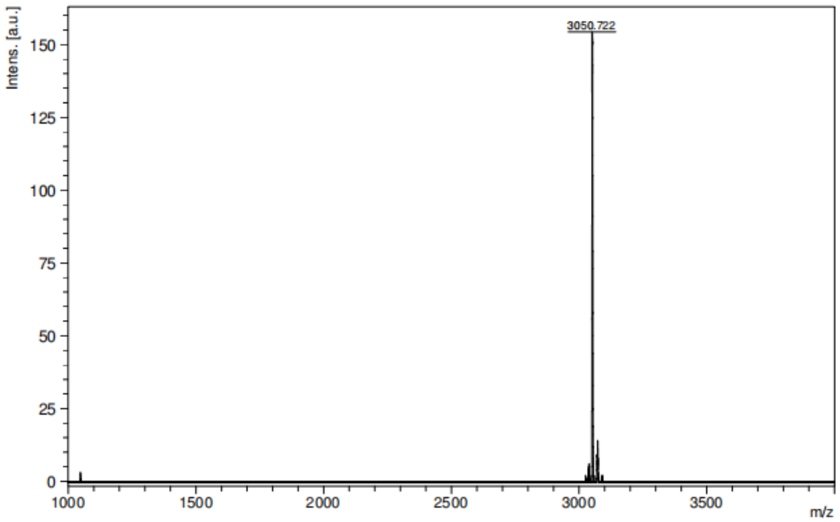

Figure S34. MALDI-TOF mass spectrum of IDAtp1-H-LinA, found mass: 3050.722.

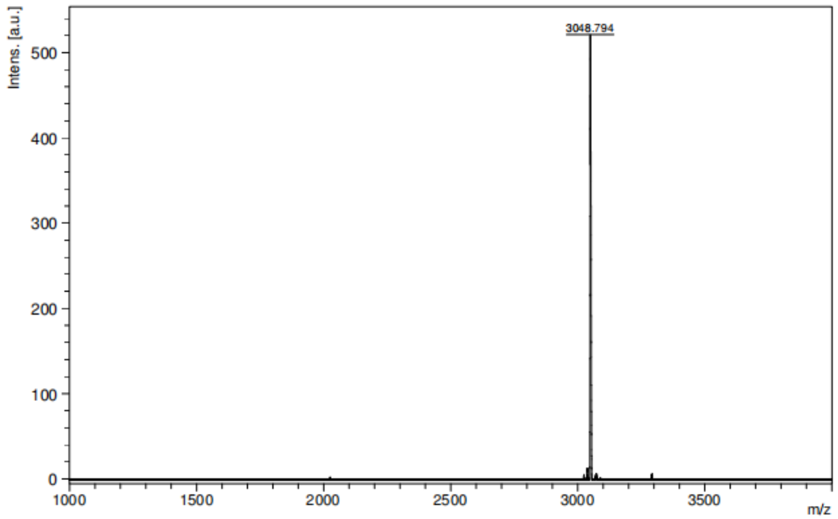

Figure S35. MALDI-TOF mass spectrum of dGtp1-H-LinA, found mass: 3048.794.

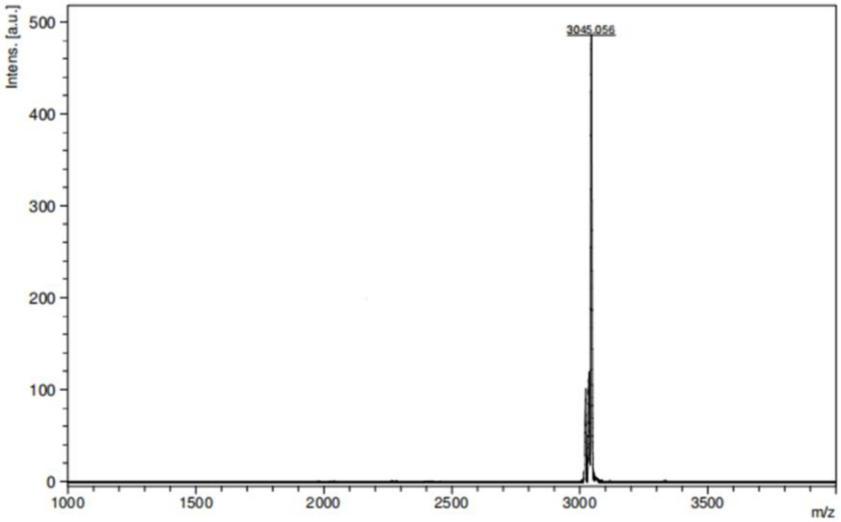

Figure S36. MALDI-TOF mass spectrum of Gtp1-H-LinA, found mass: 3045.056.

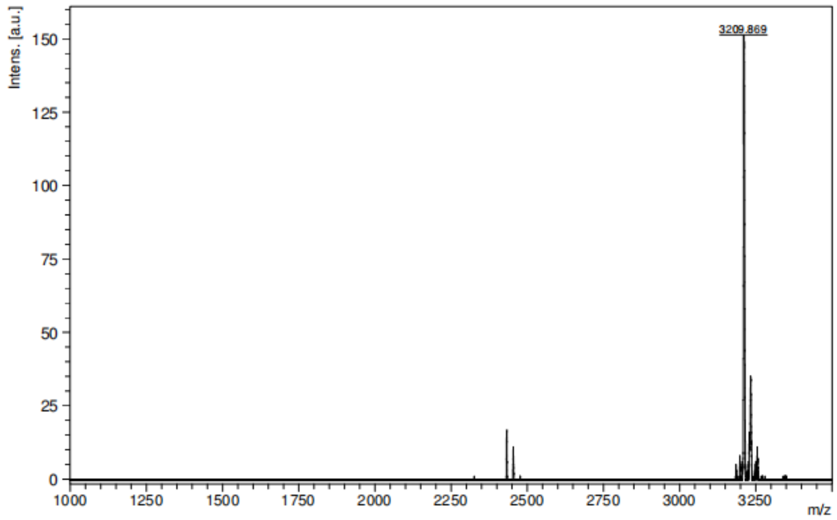

Figure S37. MALDI-TOF mass spectrum of TFE-IDAtp1-H-LinA, found mass: 3209.869.

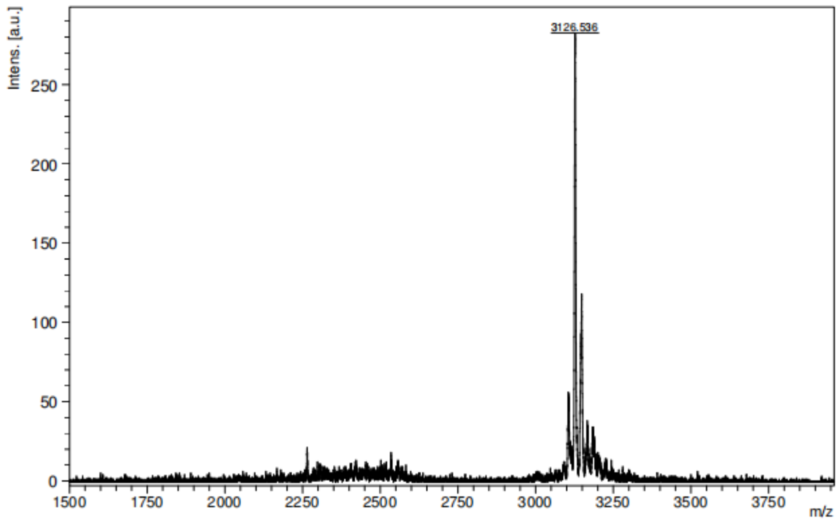

Figure S38. MALDI-TOF mass spectrum of Htp1-H-LinA, found mass: 3126.536.

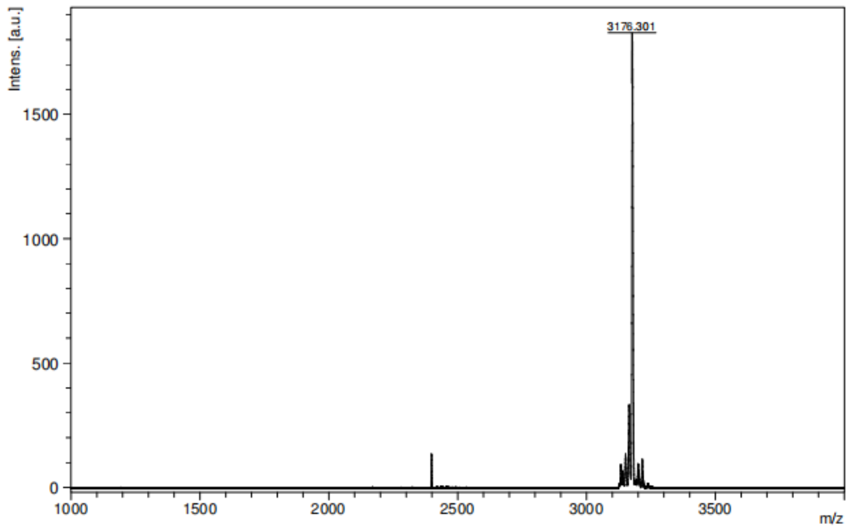

Figure S39. MALDI-TOF mass spectrum of chGtp1-H-LinA, found mass: 3176.301.

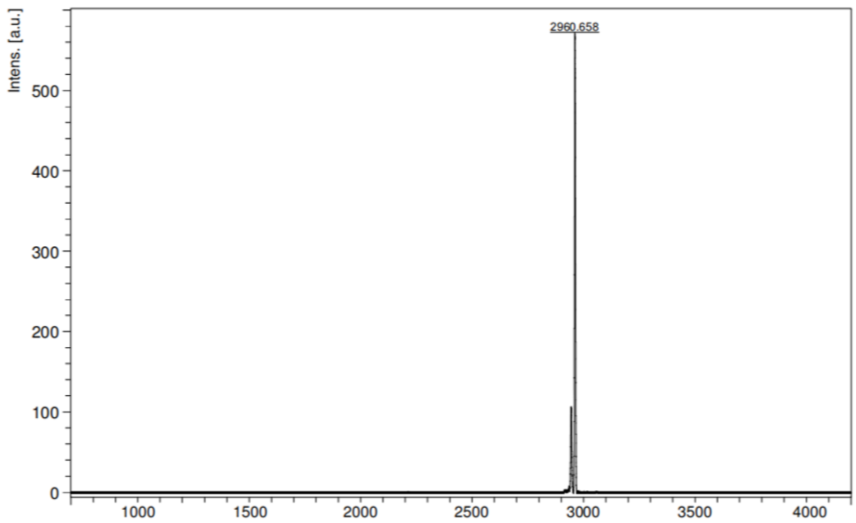

Figure S40. MALDI-TOF mass spectrum of Gtt1-H-LinA, found mass: 2960.658.

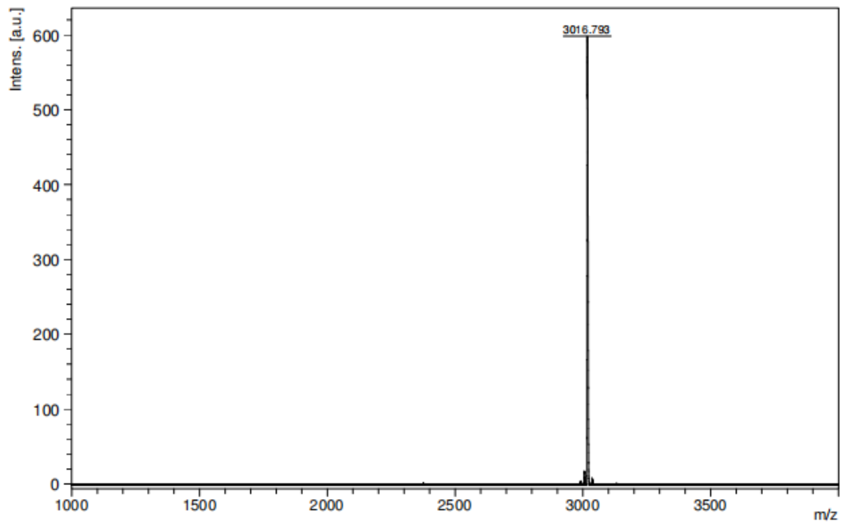

Figure S41. MALDI-TOF mass spectrum of GEIPA1-H-LinA, found mass: 3016.793.

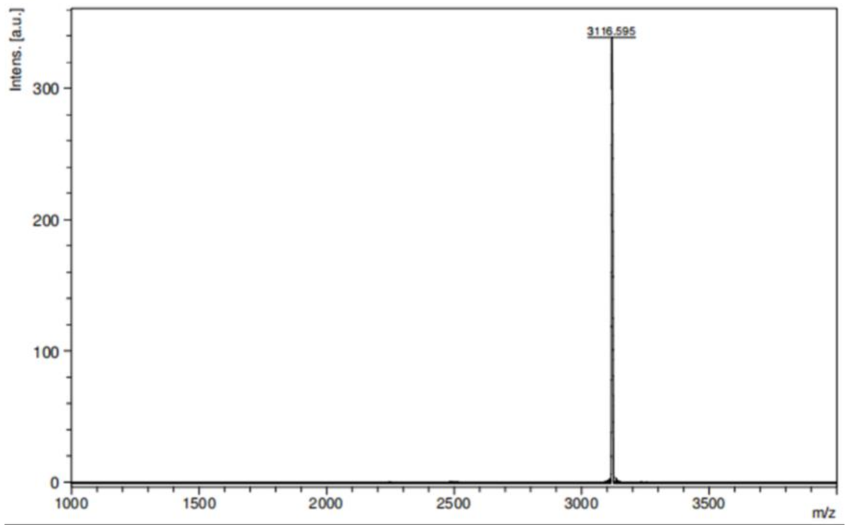

Figure S42. MALDI-TOF mass spectrum of Ptp1-H-LinA, found mass: 3116.595.

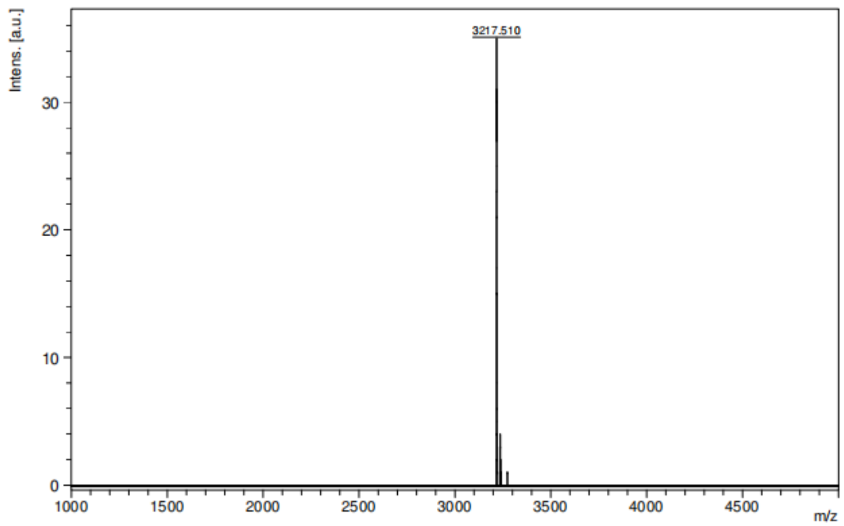

Figure S43. MALDI-TOF mass spectrum of Ntp1-H-LinA, found mass: 3217.510.

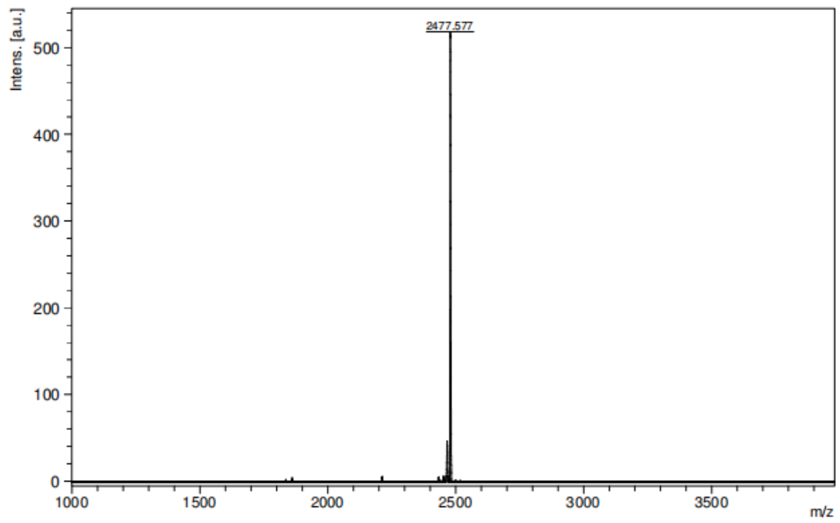

Figure S44. MALDI-TOF mass spectrum of Stp1-LinA, found mass: 2477.577.

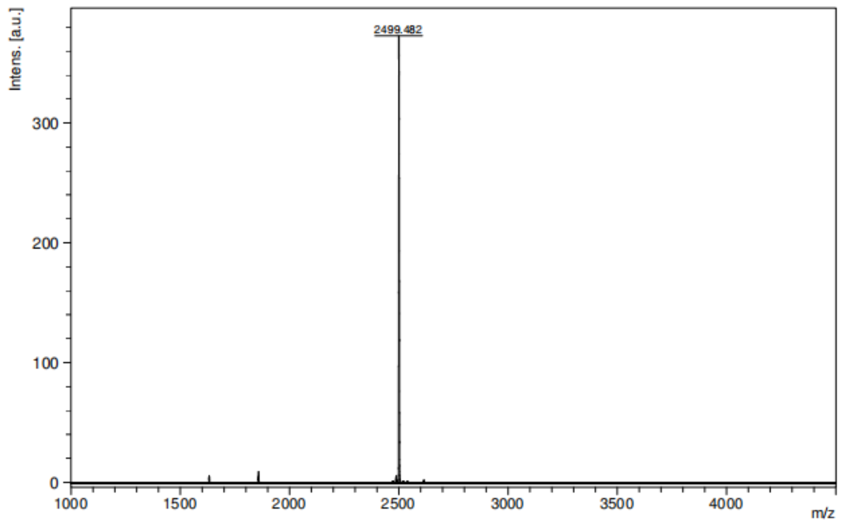

Figure S45. MALDI-TOF mass spectrum of IDAtp1-LinA, found mass: 2499.482.

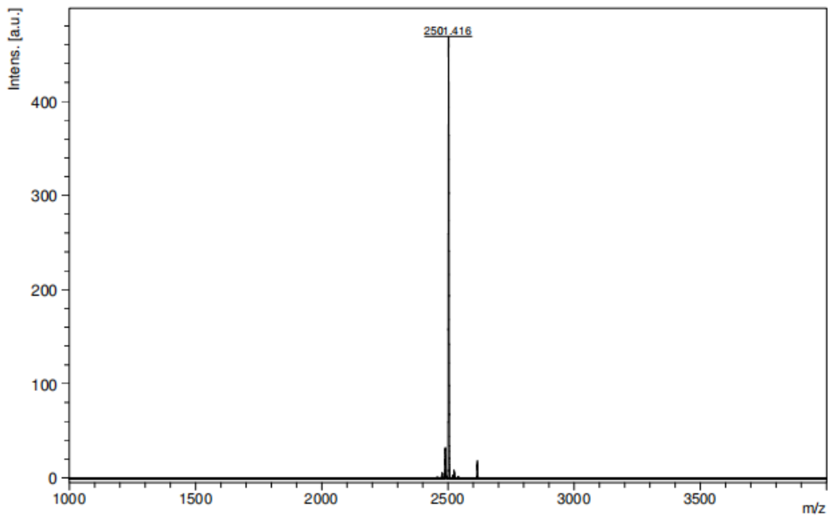

Figure S46. MALDI-TOF mass spectrum of dGtp1-LinA, found mass: 2501.416.

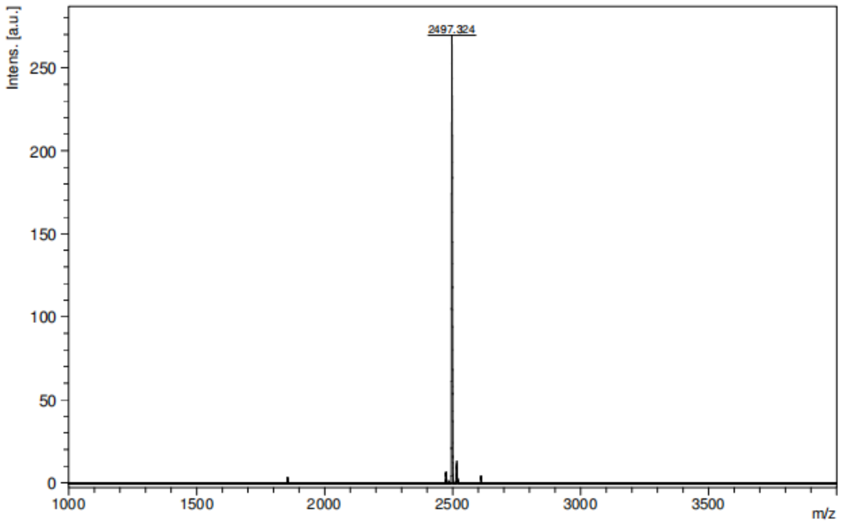

Figure S47. MALDI-TOF mass spectrum of Gtp1-LinA, found mass: 2497.324.

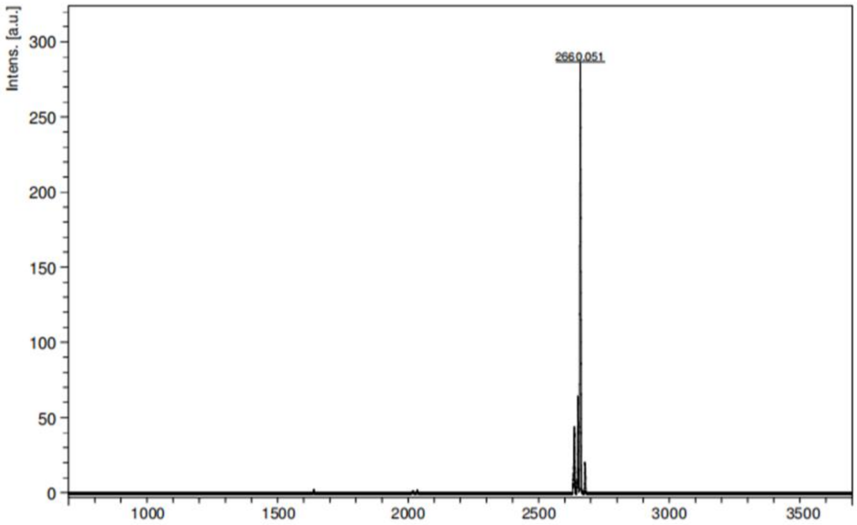

Figure S48. MALDI-TOF mass spectrum of TFE-IDAtp1-LinA, found mass: 2660.051.

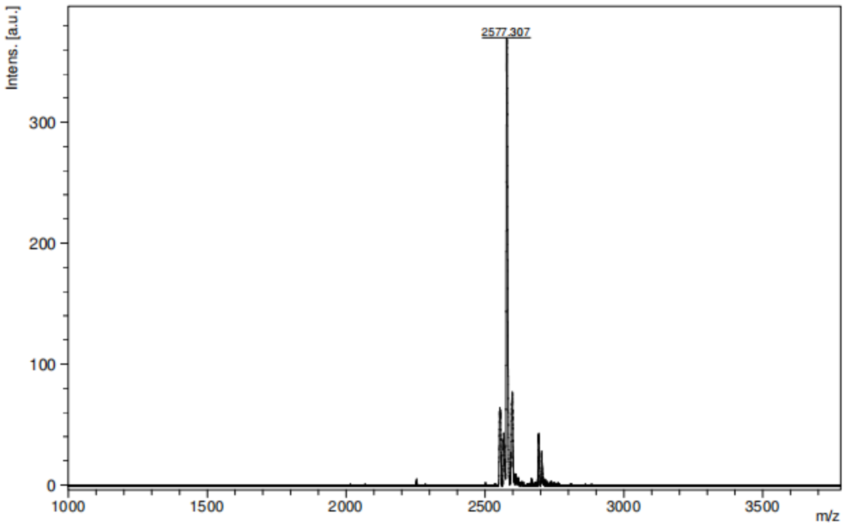

Figure S49. MALDI-TOF mass spectrum of Htp1-LinA, found mass: 2577.307.

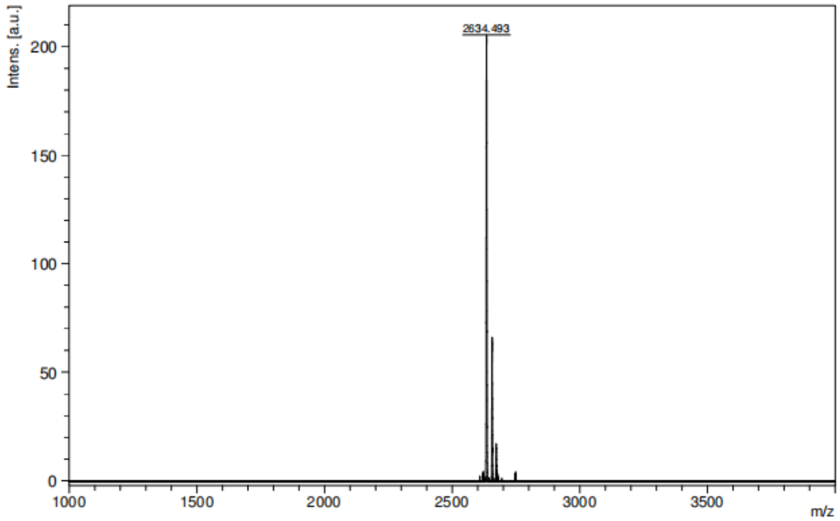

Figure S50. MALDI-TOF mass spectrum of chGtp1-LinA, found mass: 2634.493.

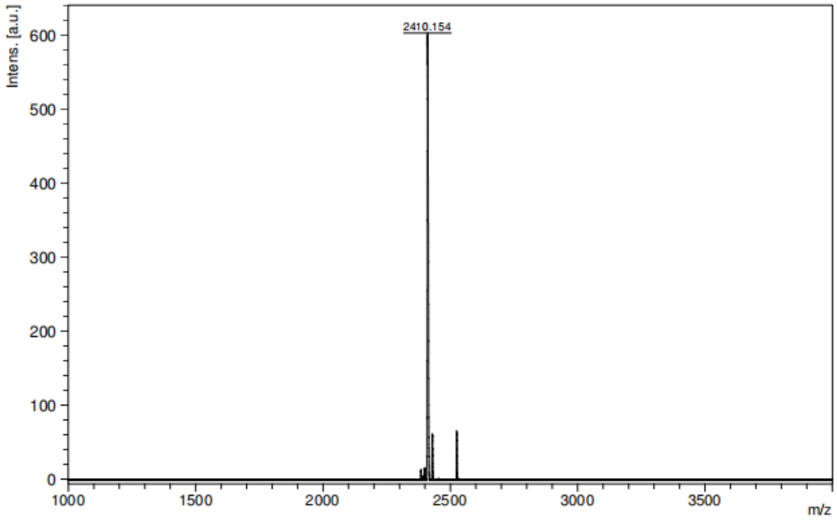

Figure S51. MALDI-TOF mass spectrum of Gtt1-LinA, found mass: 2410.154.

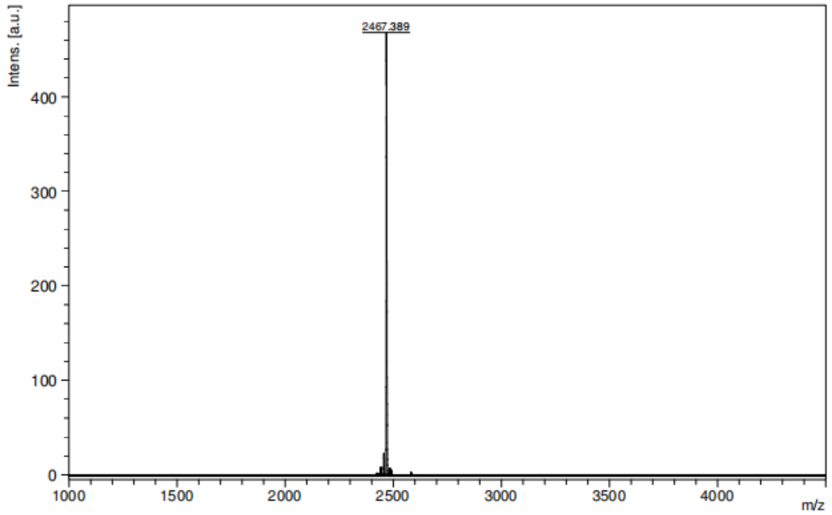

Figure S52. MALDI-TOF mass spectrum of GEIPA1-LinA, found mass: 2467.389.

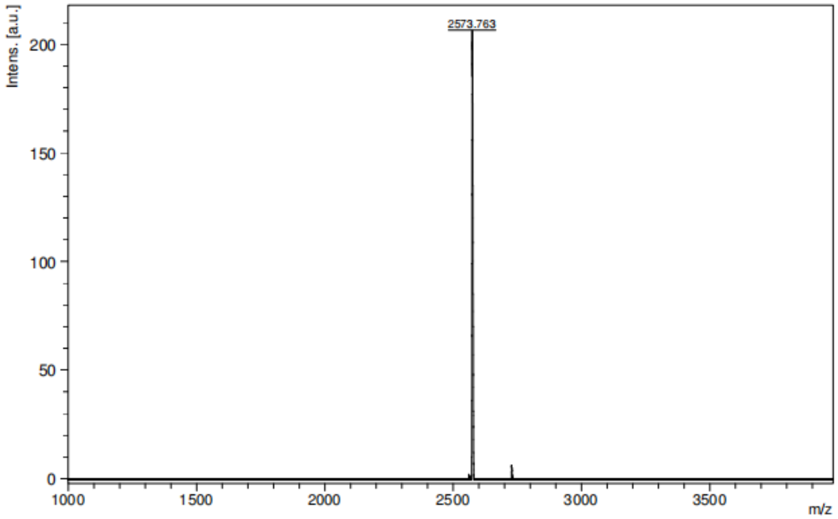

Figure S53. MALDI-TOF mass spectrum of Ptp1-LinA, found mass: 2573.763.

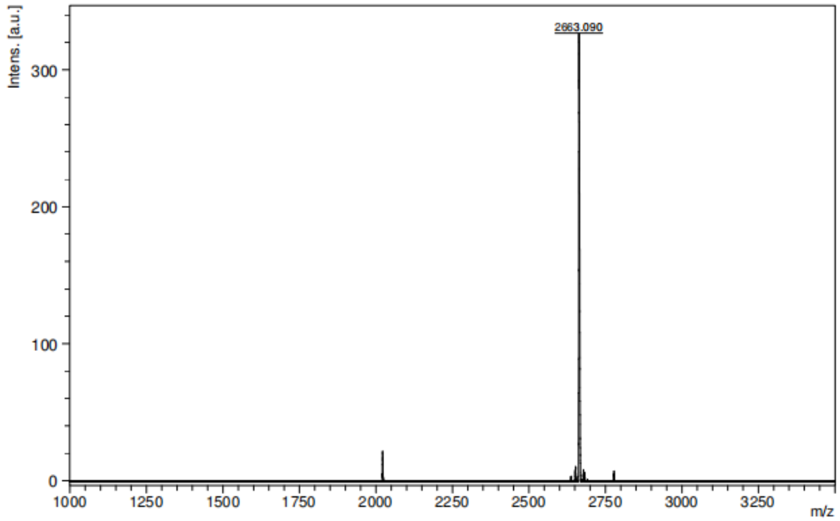

Figure S54. MALDI-TOF mass spectrum of Ntp1-LinA, found mass: 2663.090.

Table S2. ID number, sequence, MALDI-MS of all xenopeptides and hydrodynamic size, PDI, zeta potential of Cas9 RNP nanocarriers formed with the xenopeptides.

ID	Sequence (N → C)	Size[a]	PDI[a]	Zeta potential[a]	MS [calc.]	MS [found]
1468	K(N3)-Y3-Stp-K(K(SteA)2)-Stp-Y3	201.5 ± 2.8	0.26 ± 0.03	15.2 ± 1.1	2481.59	2480.21
1391	K(N3)-Y3-Stp-K(K(OleA)2)-Stp-Y3	152.3 ± 3.8	0.29 ± 0.03	12.6 ± 1.9	2477.56	2473.18
1392	K(N3)-Y3-Stp-K(K(LinA)2)-Stp-Y3	159.3 ± 1.5	0.20 ± 0.04	12.8 ± 0.4	2473.53	2477.58
1393	K(N3)-Y3-Stp-K(K(LenA)2)-Stp-Y3	173.9 ± 4.0	0.27 ± 0.03	11.7 ± 0.9	2469.50	2463.12
1394	K(N3)-Y3-Stp-K(K(CholA)2)-Stp-Y3	194.4 ± 1.0	0.38 ± 0.03	14.4 ± 1.3	2633.65	2630.41
1469	K(N3)-Y3-Stp-K(K(OHSteA)2)-Stp-Y3	161.2 ± 4.1	0.26 ± 0.03	13.4 ± 0.2	2513.58	2510.21
1470	K(N3)-Y3-H-Stp-H-K(K(SteA)2)-H-Stp-H-Y3	204.9 ± 5.7	0.16 ± 0.04	14.9 ± 1.9	3029.83	3024.77
1395	K(N3)-Y3-H-Stp-H-K(K(OleA)2)-H-Stp-H-Y3	200.2 ± 3.5	0.19 ± 0.02	11.0 ± 1.4	3025.79	3020.04
1396	K(N3)-Y3-H-Stp-H-K(K(LinA)2)-H-Stp-H-Y3	194.9 ± 3.8	0.22 ± 0.03	10.3 ± 1.3	3021.76	3021.27
1397	K(N3)-Y3-H-Stp-H-K(K(LenA)2)-H-Stp-H-Y3	185.7 ± 0.9	0.35 ± 0.03	12.2 ± 1.5	3017.73	3013.67
1398	K(N3)-Y3-H-Stp-H-K(K(CholA)2)-H-Stp-H-Y3	167.1 ± 5.7	0.15 ± 0.03	16.1 ± 1.2	3181.89	3177.96
1471	K(N3)-Y3-H-Stp-H-K(K(OHSteA)2)-H-Stp-H-Y3	193.8 ± 4.4	0.22 ± 0.02	16.0 ± 0.6	3061.81	3055.77
1581	K(N3)-C-Y3-Stp-K(K(SteA)2)-Stp-Y3-C	190.3 ± 5.2	0.31 ± 0.01	15.2 ± 1.6	2687.61	2683.51
1577	K(N3)-C-Y3-Stp-K(K(OleA)2)-Stp-Y3-C	156.2 ± 3.9	0.40 ± 0.05	13.2 ± 1.7	2683.58	2678.47
1578	K(N3)-C-Y3-Stp-K(K(LinA)2)-Stp-Y3-C	147.0 ± 3.4	0.16 ± 0.02	14.0 ± 0.2	2679.55	2673.43
1579	K(N3)-C-Y3-Stp-K(K(LenA)2)-Stp-Y3-C	151.5 ± 6.8	0.29 ± 0.03	11.5 ± 0.2	2675.51	2671.4
1580	K(N3)-C-Y3-Stp-K(K(CholA)2)-Stp-Y3-C	190.1 ± 6.0	0.19 ± 0.03	14.1 ± 0.8	2839.67	2833.69
1582	K(N3)-C-Y3-Stp-K(K(OHSteA)2)-Stp-Y3-C	180.2 ± 4.3	0.24 ± 0.02	12.2 ± 1.2	2719.60	2713.53
1587	K(N3)-C-Y3-H-Stp-H-K(K(SteA)2)-H-Stp-H-Y3-C	245.1 ± 10.3	0.27 ± 0.02	14.1 ± 0.1	3235.84	3230.05
1583	K(N3)-C-Y3-H-Stp-H-K(K(OleA)2)-H-Stp-H-Y3-C	195.3 ± 5.8	0.15 ± 0.01	12.3 ± 1.9	3231.81	3228.02
1584	K(N3)-C-Y3-H-Stp-H-K(K(LinA)2)-H-Stp-H-Y3-C	235.8 ± 9.5	0.23 ± 0.04	12.8 ± 1.7	3227.78	3223.99
1585	K(N3)-C-Y3-H-Stp-H-K(K(LenA)2)-H-Stp-H-Y3-C	202.4 ± 8.7	0.39 ± 0.04	13.4 ± 0.7	3223.75	3218.96
1586	K(N3)-C-Y3-H-Stp-H-K(K(CholA)2)-H-Stp-H-Y3-C	199.0 ± 8.6	0.36 ± 0.03	16.1 ± 0.6	3387.91	3383.24
1588	K(N3)-C-Y3-H-Stp-H-K(K(OHSteA)2)-H-Stp-H-Y3-C	190.1 ± 6.0	0.20 ± 0.03	12.9 ± 0.7	3267.83	3261.05
1361	K(N3)-Y3-Stp2-K(K(SteA)2)-Stp2-Y3	377.5 ± 56.6	0.37 ± 0.01	17.5 ± 1.4	3023.99	3017.93
1208	K(N3)-Y3-Stp2-K(K(OleA)2)-Stp2-Y3	379.2 ± 70.5	0.29 ± 0.03	14.1 ± 0.4	3019.96	3013.69
1399	K(N3)-Y3-Stp2-K(K(LinA)2)-Stp2-Y3	345.5 ± 33.8	0.29 ± 0.01	13.1 ± 0.9	3015.93	3011.87
1400	K(N3)-Y3-Stp2-K(K(LenA)2)-Stp2-Y3	385.0 ± 47.5	0.35 ± 0.03	14.3 ± 1.6	3011.90	3005.84
1364	K(N3)-Y3-Stp2-K(K(CholA)2)-Stp2-Y3	501.6 ± 52.6	0.41 ± 0.01	13.0 ± 1.9	3176.05	3170.12
1573	K(N3)-Y3-Stp2-K(K(OHSteA)2)-Stp2-Y3	440.3 ± 33.2	0.24 ± 0.01	14.3 ± 0.3	3055.98	3051.93
1321	K(N3)-Y3-(H-Stp)2-H-K(K(SteA)2)-H-(Stp-H)2-Y3	340.2 ± 31.2	0.16 ± 0.04	14.3 ± 1.5	3846.34	3840.77
1209	K(N3)-Y3-(H-Stp)2-H-K(K(OleA)2)-H-(Stp-H)2-Y3	338.3 ± 28.0	0.28 ± 0.05	14.9 ± 0.5	3842.31	3836.73

1401	K(N3)-Y3-(H-Stp)2-H-K(K(LinA)2)-H-(Stp-H)2-Y3	478.7 ± 8.1	0.16 ± 0.02	11.1 ± 1.5	3838.28	3832.71
1402	K(N3)-Y3-(H-Stp)2-H-K(K(LenA)2)-H-(Stp-H)2-Y3	288.5 ± 17.5	0.45 ± 0.04	11.9 ± 0.5	3834.25	3830.67
1403	K(N3)-Y3-(H-Stp)2-H-K(K(CholA)2)-H-(Stp-H)2-Y3	366.8 ± 37.7	0.40 ± 0.04	13.9 ± 1.0	3998.41	3995.96
1574	K(N3)-Y3-(H-Stp)2-H-K(K(OHSteA)2)-H-(Stp-H)2-Y3	256.4 ± 11.5	0.37 ± 0.03	12.4 ± 0.3	3878.33	3872.76
1337	K(N3)-C-Y3-Stp2-K(K(SteA)2)-Stp2-Y3-C	201.4 ± 10.6	0.29 ± 0.03	16.7 ± 1.9	3230.01	3226.22
1338	K(N3)-C-Y3-Stp2-K(K(OleA)2)-Stp2-Y3-C	166.6 ± 5.8	0.25 ± 0.03	16.6 ± 0.4	3225.98	3221.18
1199	K(N3)-C-Y3-Stp2-K(K(LinA)2)-Stp2-Y3-C	159.3 ± 5.9	0.23 ± 0.02	15.9 ± 1.3	3221.95	3217.15
1200	K(N3)-C-Y3-Stp2-K(K(LenA)2)-Stp2-Y3-C	160.6 ± 4.3	0.17 ± 0.03	16.8 ± 1.2	3217.92	3211.12
1340	K(N3)-C-Y3-Stp2-K(K(CholA)2)-Stp2-Y3-C	203.4 ± 6.9	0.28 ± 0.03	15.4 ± 0.8	3382.07	3376.41
1445	K(N3)-C-Y3-Stp2-K(K(OHSteA)2)-Stp2-Y3-C	177.7 ± 5.9	0.25 ± 0.04	16.5 ± 1.7	3262.00	3256.56
1278	K(N3)-C-Y3-(H-Stp)2-H-K(K(SteA)2)-H-(Stp-H)2-Y3-C	205.1 ± 12.9	0.20 ± 0.04	15.1 ± 1.1	4052.36	4048.05
1214	K(N3)-C-Y3-(H-Stp)2-H-K(K(OleA)2)-H-(Stp-H)2-Y3-C	185.5 ± 5.7	0.26 ± 0.05	18.5 ± 1.5	4048.33	4042.02
1389	K(N3)-C-Y3-(H-Stp)2-H-K(K(LinA)2)-H-(Stp-H)2-Y3-C	178.5 ± 10.2	0.30 ± 0.02	16.8 ± 1.2	4044.30	4038.99
1390	K(N3)-C-Y3-(H-Stp)2-H-K(K(LenA)2)-H-(Stp-H)2-Y3-C	188.8 ± 9.9	0.28 ± 0.02	15.8 ± 0.6	4042.96	4036.96
1575	K(N3)-C-Y3-(H-Stp)2-H-K(K(CholA)2)-H-(Stp-H)2-Y3-C	200.7 ± 10.3	0.33 ± 0.03	14.7 ± 1.4	4204.43	4200.25
1576	K(N3)-C-Y3-(H-Stp)2-H-K(K(OHSteA)2)-H-(Stp-H)2-Y3-C	191.6 ± 6.3	0.33 ± 0.07	14.6 ± 0.6	4084.35	4078.06
1639	K(N3)-Y3-Gtp-K(K(LinA)2)-Gtp-Y3	156.3 ± 2.9	0.15 ± 0.04	13.1 ± 0.8	2501.56	2497.32
1641	K(N3)-Y3-Htp-K(K(LinA)2)-Htp-Y3	165.3 ± 2.7	0.22 ± 0.05	12.1 ± 0.9	2581.62	2577.31
1642	K(N3)-Y3-Ptp-K(K(LinA)2)-Ptp-Y3	168.1 ± 3.2	0.33 ± 0.01	10.9 ± 1.9	2569.53	2573.76
1643	K(N3)-Y3-Ntp-K(K(LinA)2)-Ntp-Y3	175.6 ± 1.1	0.26 ± 0.02	11.3 ± 1.3	2669.56	2663.09
1644	K(N3)-Y3-Gtt-K(K(LinA)2)-Gtt-Y3	165.3 ± 10.0	0.22 ± 0.03	11.5 ± 0.9	2415.47	2410.15
1645	K(N3)-Y3-GEIPA-K(K(LinA)2)-GEIPA-Y3	169.3 ± 9.1	0.28 ± 0.04	11.6 ± 0.8	2471.54	2467.39
1737	K(N3)-Y3-chGtp-K(K(LinA)2)-chGtp-Y3	176.3 ± 3.5	0.31 ± 0.05	12.5 ± 1.9	2637.68	2634.49
1738	K(N3)-Y3-dGtp-K(K(LinA)2)-dGtp-Y3	173.5 ± 1.3	0.20 ± 0.03	13.9 ± 2.9	2505.52	2501.42
1739	K(N3)-Y3-(TFE-IDAtp)-K(K(LinA)2)-(TFE-IDAtp)-Y3	166.3 ± 6.1	0.29 ± 0.03	12.5 ± 1.1	2667.55	2660.05
1740	K(N3)-Y3-IDAtp-K(K(LinA)2)-IDAtp-Y3	138.9 ± 9.0	0.31 ± 0.03	13.6 ± 3.1	2503.55	2499.48
1647	K(N3)-Y3-H-Gtp-H-K(K(LinA)2)-H-Gtp-H-Y3	165.6 ± 3.2	0.28 ± 0.01	12.1 ± 1.3	3049.79	3045.06
1649	K(N3)-Y3-H-Htp-H-K(K(LinA)2)-H-Htp-H-Y3	178.5 ± 3.2	0.25 ± 0.02	11.9 ± 0.3	3129.86	3126.54
1650	K(N3)-Y3-H-Ptp-H-K(K(LinA)2)-H-Ptp-H-Y3	180.3 ± 6.1	0.38 ± 0.03	10.5 ± 2.1	3117.76	3116.60
1651	K(N3)-Y3-H-Ntp-H-K(K(LinA)2)-H-Ntp-H-Y3	178.5 ± 3.2	0.35 ± 0.05	10.9 ± 1.6	3217.79	3217.51
1652	K(N3)-Y3-H-Gtt-H-K(K(LinA)2)-H-Gtt-H-Y3	180.5 ± 1.2	0.28 ± 0.02	11.6 ± 1.3	2963.71	2960.66
1653	K(N3)-Y3-H-GEIPA-H-K(K(LinA)2)-H-GEIPA-H-Y3	179.3 ± 2.3	0.25 ± 0.01	13.1 ± 3.2	3019.77	3016.79
1741	K(N3)-Y3-H-chGtp-H-K(K(LinA)2)-H-chGtp-H-Y3	175.4 ± 3.6	0.31 ± 0.02	11.8 ± 1.3	3185.92	3176.30
1742	K(N3)-Y3-H-dGtp-H-K(K(LinA)2)-H-dGtp-H-Y3	169.6 ± 3.9	0.22 ± 0.01	13.2 ± 1.4	3053.75	3048.79
1743	K(N3)-Y3-H-(TFE-IDAtp)-H-K(K(LinA)2)-H-(TFE-IDAtp)-H-Y3	185.6 ± 4.7	0.25 ± 0.03	12.8 ± 0.6	3215.79	3209.87

1744	K(N3)-Y3-H-IDAtp-H-K(K(LinA)2)-H-IDAtp-H-Y3	189.1 ± 4.2	0.19 ± 0.02	13.7 ± 1.2	3051.78	3050.72
1631	K(N3)-C-Y3-Gtp2-K(K(OHSteA)2)-Gtp2-Y3-C	168.3 ± 3.9	0.29 ± 0.02	16.0 ± 2.0	3318.06	3316.35
1633	K(N3)-C-Y3-Htp2-K(K(OHSteA)2)-Htp2-Y3-C	183.6 ± 4.5	0.36 ± 0.01	15.4 ± 1.2	3478.19	3477.14
1634	K(N3)-C-Y3-Ptp2-K(K(OHSteA)2)-Ptp2-Y3-C	190.3 ± 5.1	0.33 ± 0.02	14.1 ± 3.6	3454.00	3456.11
1635	K(N3)-C-Y3-Ntp2-K(K(OHSteA)2)-Ntp2-Y3-C	167.2 ± 3.0	0.29 ± 0.02	14.8 ± 2.8	3654.06	3650.01
1636	K(N3)-C-Y3-Gtt2-K(K(OHSteA)2)-Gtt2-Y3-C	189.8 ± 9.1	0.34 ± 0.05	13.2 ± 1.8	3145.89	3139.57
1637	K(N3)-C-Y3-GEIPA2-K(K(OHSteA)2)-GEIPA2-Y3-C	179.8 ± 5.3	0.28 ± 0.02	13.4 ± 2.1	3258.02	3252.99
1733	K(N3)-C-Y3-chGtp2-K(K(OHSteA)2)-chGtp2-Y3-C	185.6 ± 6.6	0.26 ± 0.03	15.9 ± 1.1	3590.31	3592.24
1734	K(N3)-C-Y3-dGtp2-K(K(OHSteA)2)-dGtp2-Y3-C	175.5 ± 3.6	0.23 ± 0.03	16.3 ± 1.3	3325.98	3320.31
1735	K(N3)-C-Y3-(TFE-IDAtp)2-K(K(OHSteA)2)-(TFE-IDAtp)2-Y3-C	173.3 ± 4.1	0.28 ± 0.03	15.5 ± 2.1	3650.06	3650.01
1736	K(N3)-C-Y3-IDAtp2-K(K(OHSteA)2)-IDAtp2-Y3-C	175.1 ± 2.2	0.31 ± 0.02	17.2 ± 1.6	3322.04	3318.22

[a] Cas9 RNP nanocarriers were prepared at a N/P ratio of 24 and an RNP concentration of 75 nM. The hydrodynamic size (z-average, nm), PDI and zeta potential of the nanocarriers were measured by DLS using a Zetasizer Nano ZS (Malvern Instruments, UK).

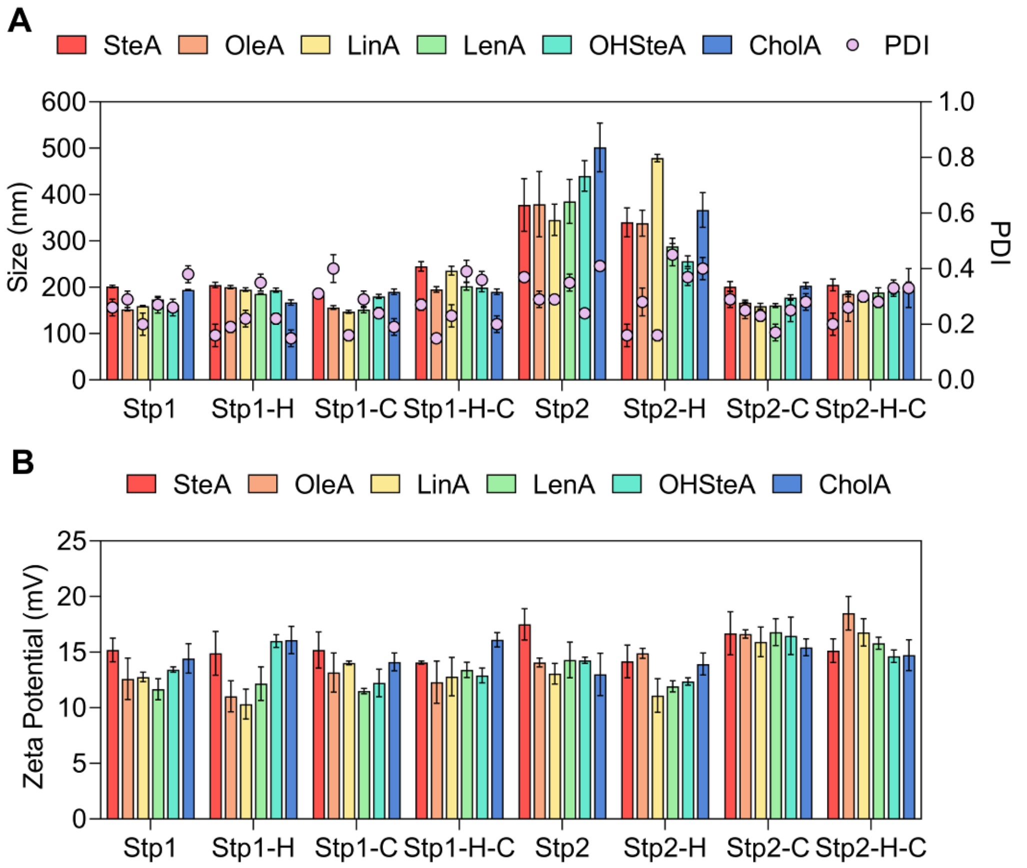

Figure S55. (A) Hydrodynamic particle size (z-average), polydispersity index (PDI) and (B) zeta potential of Cas9 RNP nanocarriers of different xenopeptides at an RNP dose of 75 nM (N/P = 24). Three technical replicates were measured.

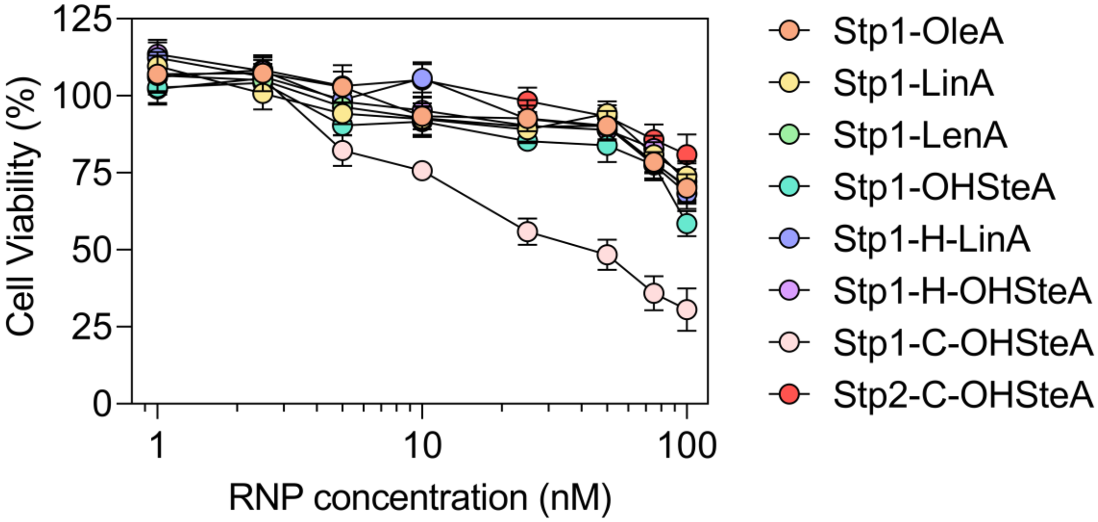

Figure S56. Cell viability of HeLa eGFP/tub cells after 48 h treatment with Cas9 RNP nanocarriers at a series of RNP concentrations ranging from 1 nM to 100 nM. Data are presented as mean ± SD (n = 3).

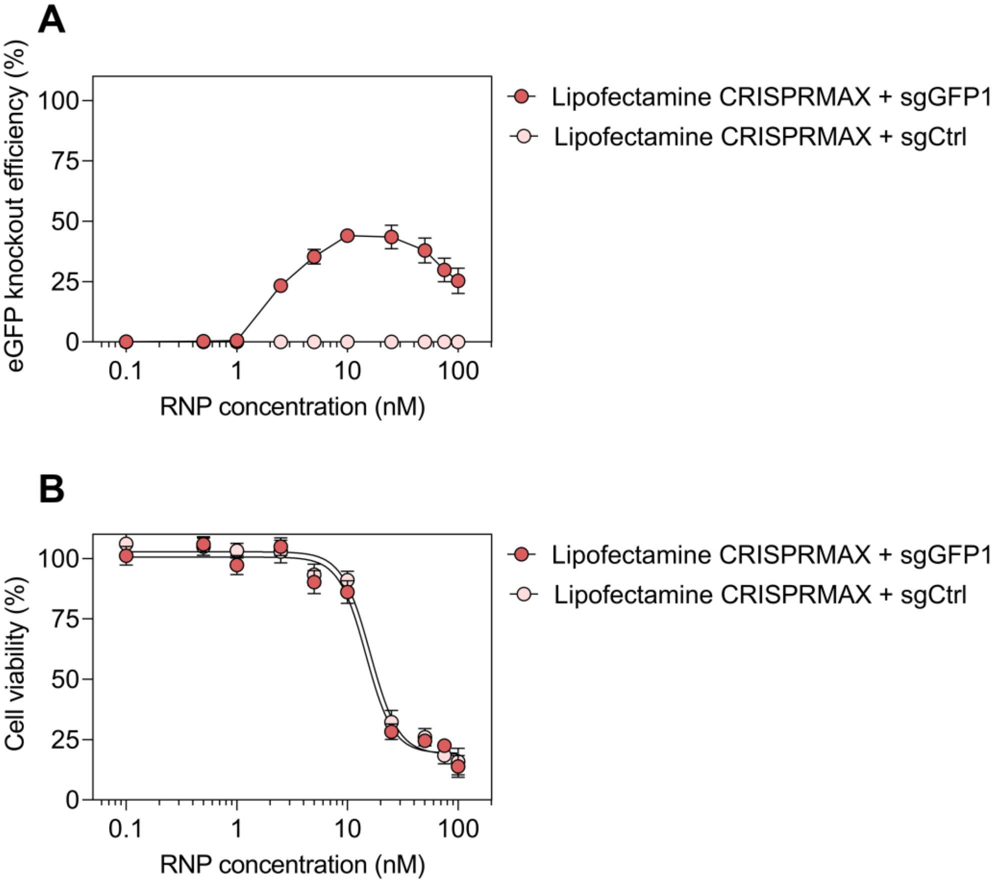

Figure S57. eGFP knockout efficiency (A) and cell viability (B) of HeLa eGFP/tub cells treated with Lipofectamine CRISPRMAX at different concentrations of Cas9/sgGFP1 RNP or Cas9/sgCtrl RNP for 48 h. Metabolic activity of the cells was determined using a MTT assay. Data are presented as % cell viability with respect to the control cells ± SD (n = 3).

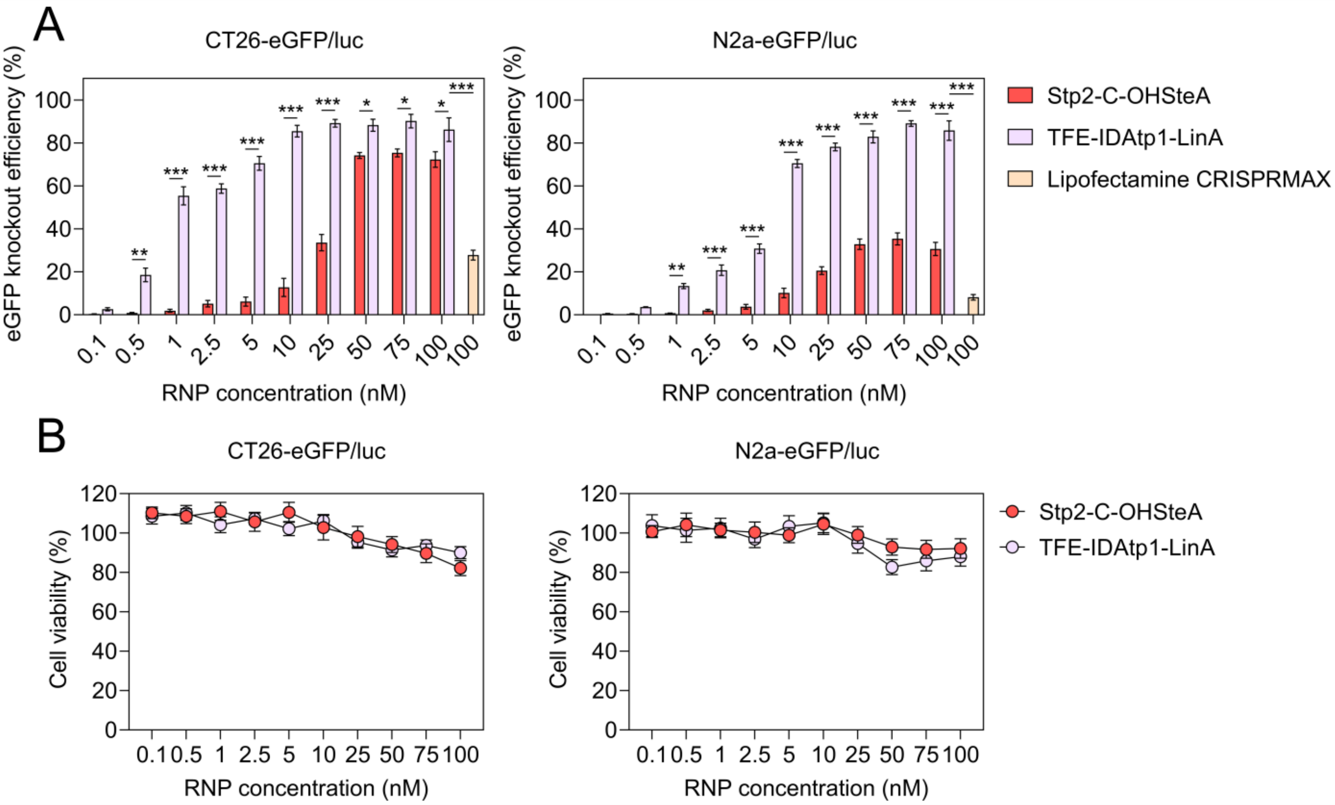

Figure S58. (A) eGFP knockout efficiency of Stp2-C-OHSteA, TFE-IDAtp1-LinA, and Lipofectamine CRISPRMAX in CT26 eGFP/luc and N2a eGFP/luc cells after 48 h treatment with Cas9 RNP nanocarriers at a series of RNP concentrations ranging from 0.1 nM to 100 nM; Data are presented as mean ± SD (n = 3). (B) Cell viability of CT26 eGFP/luc and N2a eGFP/luc cells treated with Stp2-C-OHSteA and TFE-IDAtp1-LinA at different concentrations of Cas9 RNP for 48 h. Metabolic activity of the cells was determined using a MTT assay. Data are presented as % cell viability with respect to the control cells ± SD (n = 3).

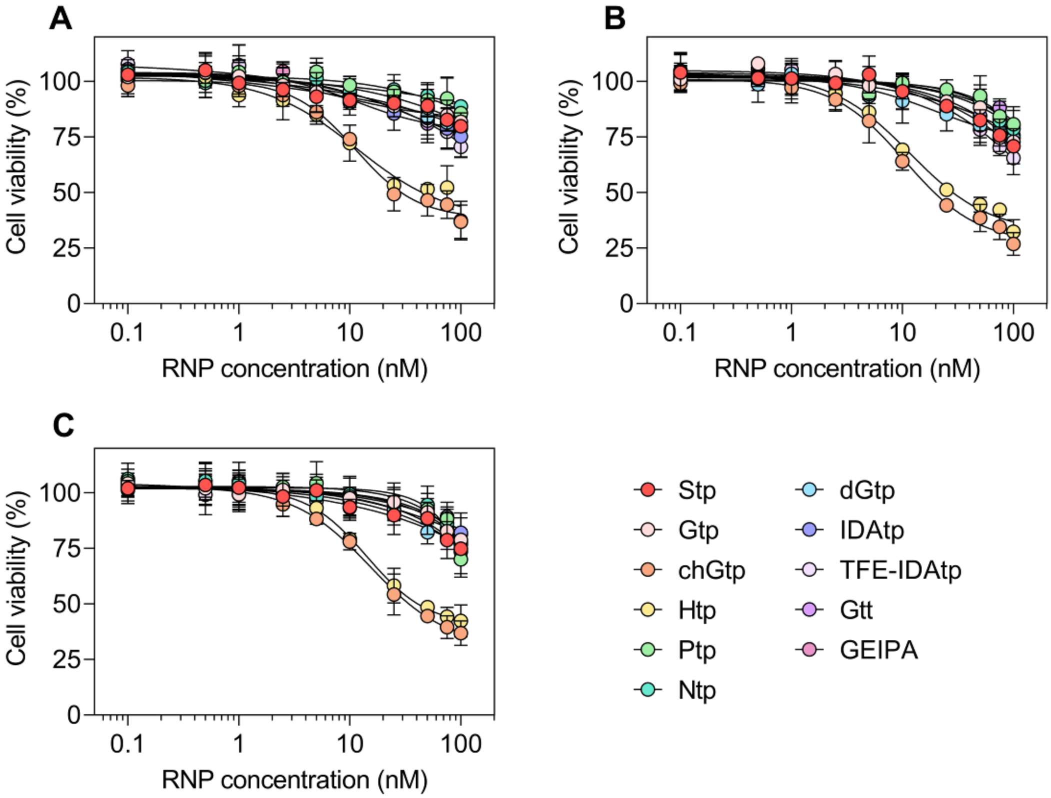

Figure S59. Cell viability of HeLa eGFP/tub cells treated with **(**A) X2-C-OHSteA, (B) X1-H-LinA, and (C) X1-LinA based nanocarriers at different concentrations of Cas9 RNP for 48 h. Metabolic activity of the cells was determined using a MTT assay. Data are presented as % cell viability with respect to the control cells ± SD (n = 3).

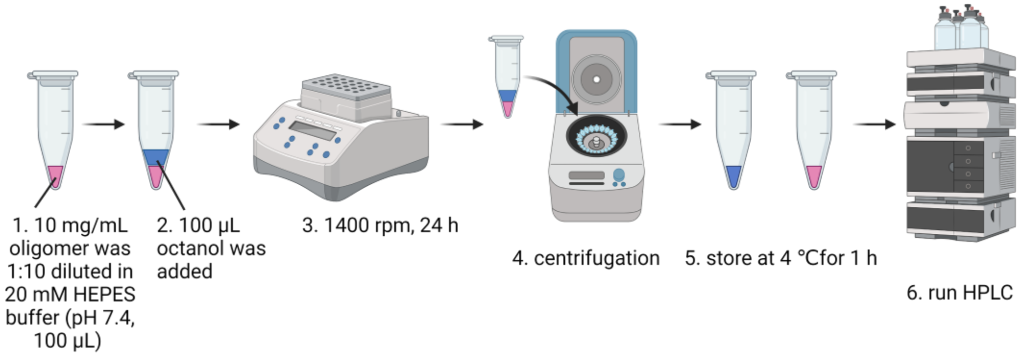

Scheme S3. Schematic illustration of $logD_{7.4}$ determination workflow. Created with BioRender.com.

Table S3. Architecture, ID number, abbreviation, and $\log D_{7.4}$, of all xenopeptides and EC50 of Cas9 RNP nanocarriers formed with the xenopeptides.

Architecture	ID	Abbreviation	$\log D_{7.4}$	EC50 (nM)[a]
X2-C-OHSteA	1445	Stp2-C-OHSteA	-1.33	14.50
	1631	Gtp2-C-OHSteA	-1.30	15.38
	1633	Htp2-C-OHSteA	-1.12	3.08
	1634	Ptp2-C-OHSteA	-0.64	18.75
	1635	Ntp2-C-OHSteA	-0.49	40.23
	1636	Gtt2-C-OHSteA	-0.85	1.60
	1637	GEIPA2-C-OHSteA	-0.80	3.18
	1733	chGtp2-C-OHSteA	-1.00	4.15
	1734	dGtp2-C-OHSteA	-1.41	35.03
	1735	TFE-IDAtp2-C-OHSteA	-1.21	2.20
	1736	IDAtp2-C-OHSteA	-1.47	17.67
X1-H-LinA	1396	Stp1-H-LinA	-1.08	0.71
	1647	Gtp1-H-LinA	-1.02	0.69
	1649	Htp1-H-LinA	-0.89	0.67
	1650	Ptp1-H-LinA	-0.48	12.24
	1651	Ntp1-H-LinA	-0.29	18.59
	1652	Gtt1-H-LinA	-0.68	0.75
	1653	GEIPA1-H-LinA	-0.63	0.51
	1741	chGtp1-H-LinA	-0.82	0.64
	1742	dGtp1-H-LinA	-1.12	6.97
	1743	TFE-IDAtp1-H-LinA	-0.99	0.65
	1744	IDAtp1-H-LinA	-1.51	5.44
X1-LinA	1392	Stp1-LinA	-0.83	0.44
	1639	Gtp1-LinA	-0.79	0.80
	1641	Htp1-LinA	-0.58	0.55
	1642	Ptp1-LinA	-0.31	2.52
	1643	Ntp1-LinA	-0.24	2.97
	1644	Gtt1-LinA	-0.37	7.36
	1645	GEIPA1 -LinA	-0.33	3.36
	1737	chGtp1-LinA	-0.48	0.53
	1738	dGtp1-LinA	-0.89	0.41
	1739	TFE-IDAtp1-LinA	-0.70	0.38
	1740	IDAtp1-LinA	-1.11	5.35

[a] EC50 (nM) is defined as the concentration of delivered Cas9 RNP required to provoke 50 % eGFP knockout in HeLa eGFP/tub cells.

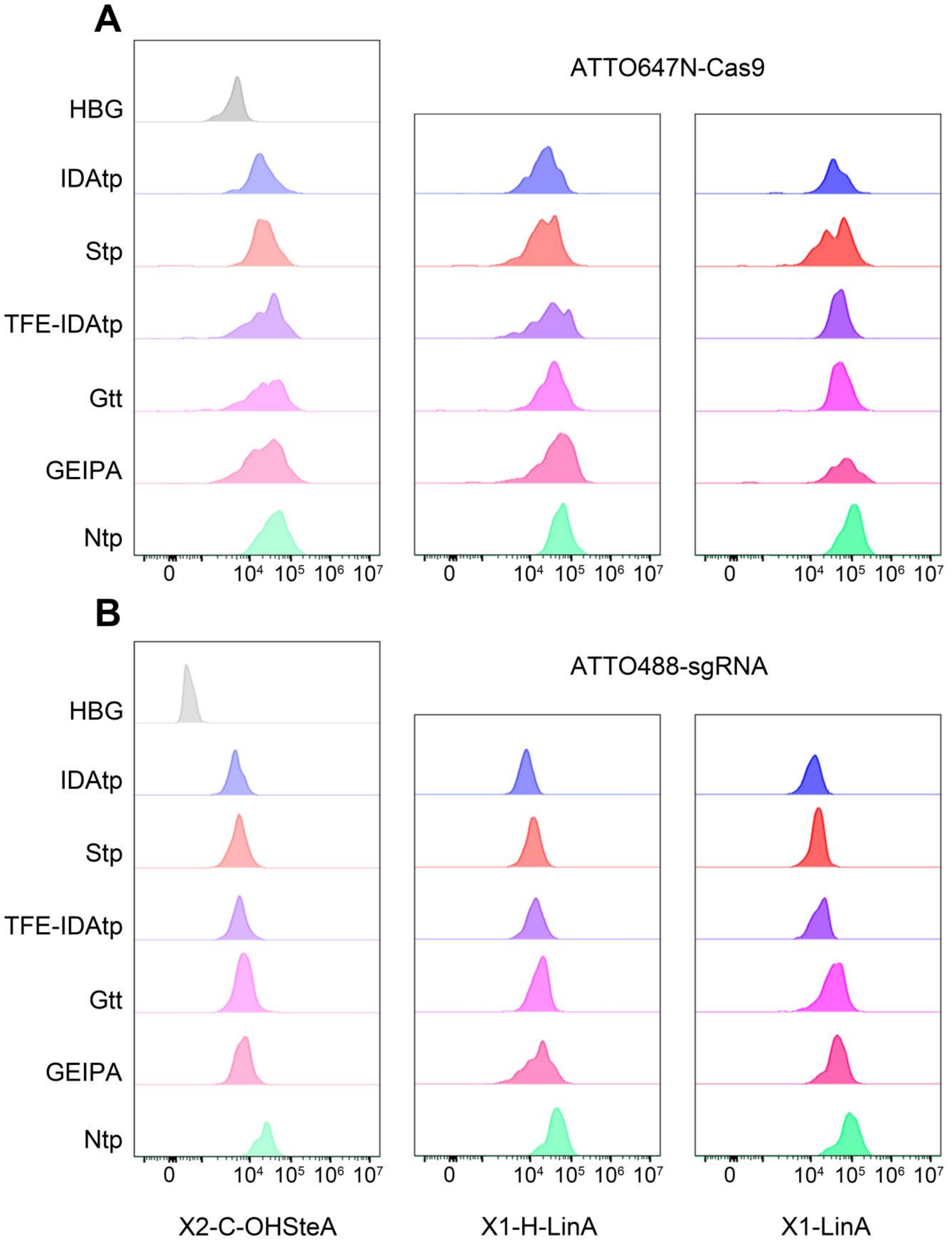

Figure S60. Flow cytometry histograms of Cas9 protein (A) and sgRNA (B) uptake of Cas9 RNP nanocarriers (75 nM RNP, 20 % ATTO647N-labeled Cas9 protein and 20 % ATTO488-labeled sgRNA) formed with different xenopeptides. Flow cytometry was conducted 4 h after RNP transfection.

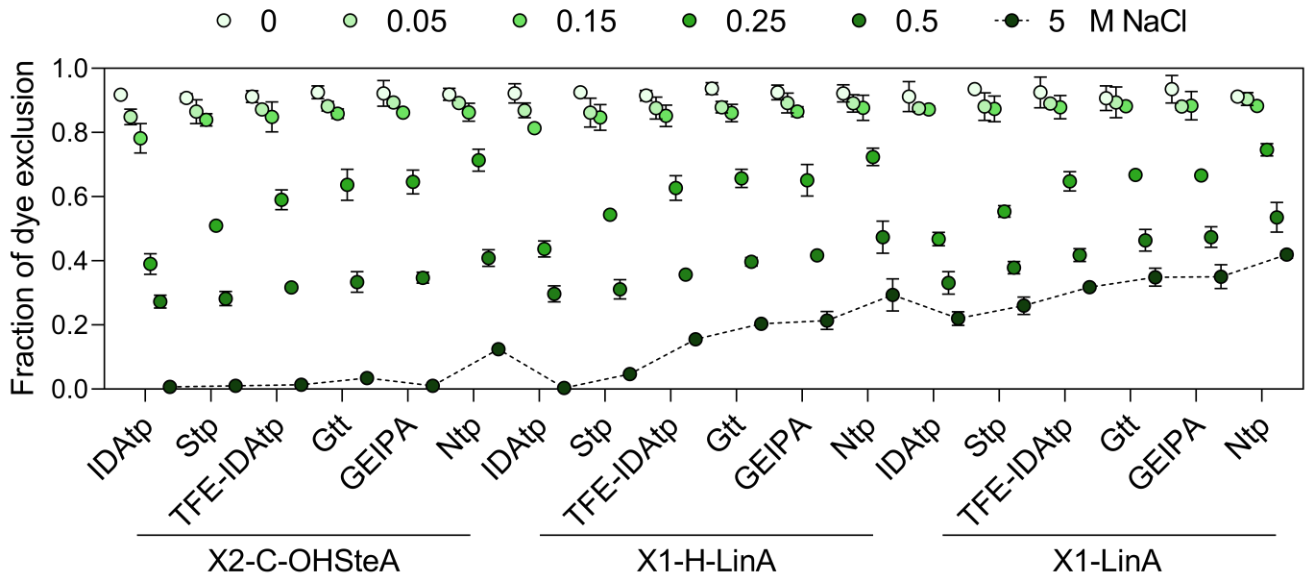

Figure S61. Nanocarrier stability against different concentrations of NaCl (0, 0.05, 0.15, 0.25, 0.5, and 5 M). Ribogreen was used for the detection of free RNA.

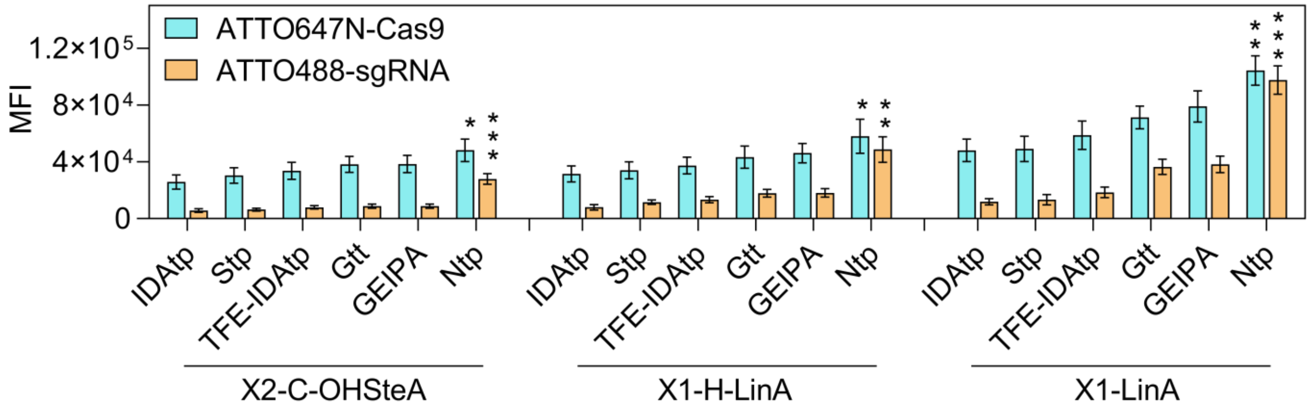

Figure S62. Cellular uptake of Cas9 RNP (75 nM) containing 20 % ATTO647N-Cas9 and 20 % ATTO488-sgRNA into HeLa cells determined by flow cytometry after 4 h incubation. The median fluorescence intensities (MFI) of ATTO647N-Cas9 protein and ATTO488-sgRNA versus xenopeptide logD$_{7.4}$ values of each xenopeptide series were also plotted as shown in Figure 4C of the main manuscript.

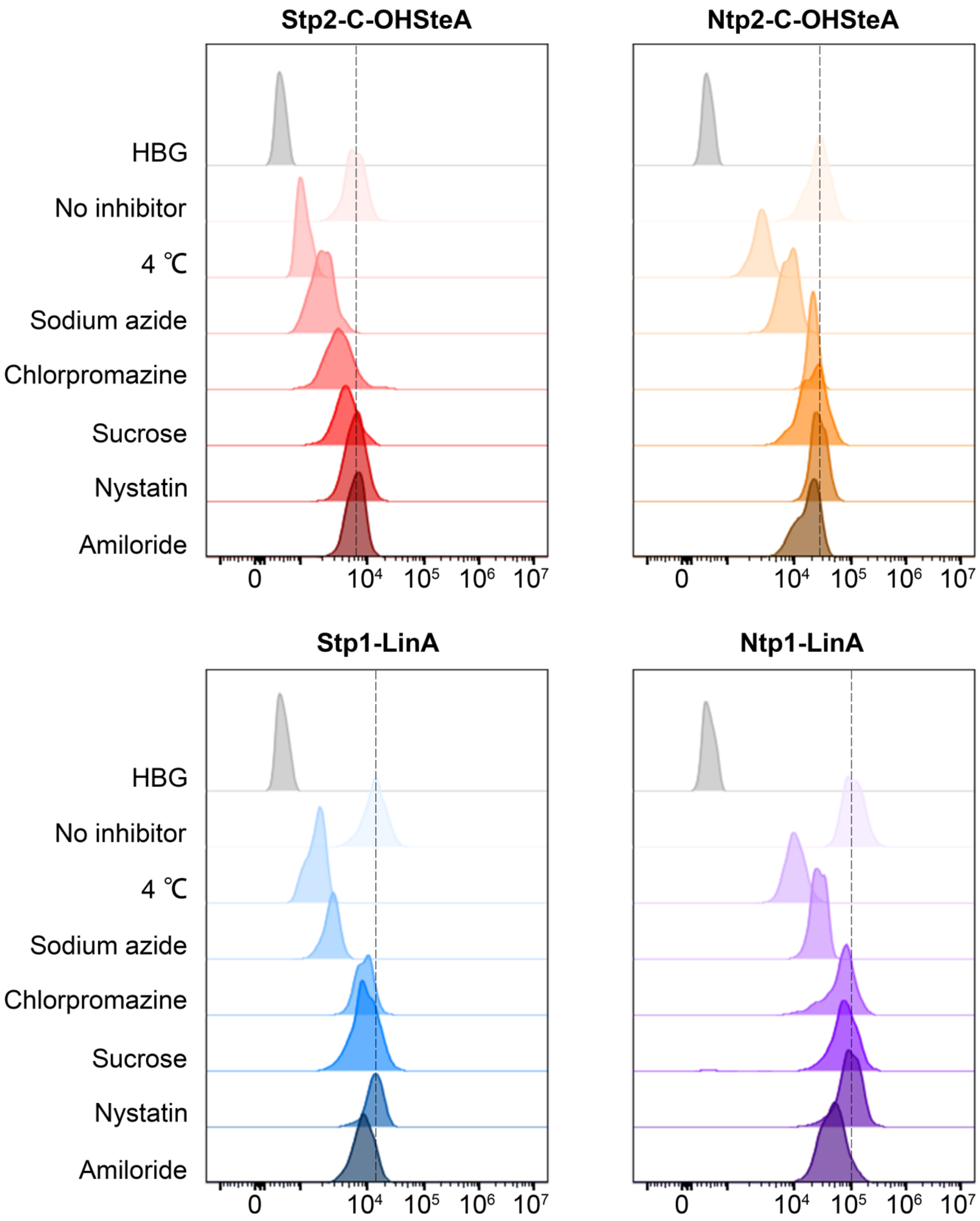

Figure S63. Flow cytometry histograms of sgRNA uptake of Cas9 RNP nanocarriers (75 nM RNP, 20 % ATTO488-labeled sgRNA) formed with **Stp2-C-OHSteA**, **Ntp2-C-OHSteA**, **Stp1-LinA**, or **Ntp1-LinA** after pre-incubation with different endocytosis inhibitors in HeLa cells. Flow cytometry was conducted 4 h after RNP transfection.

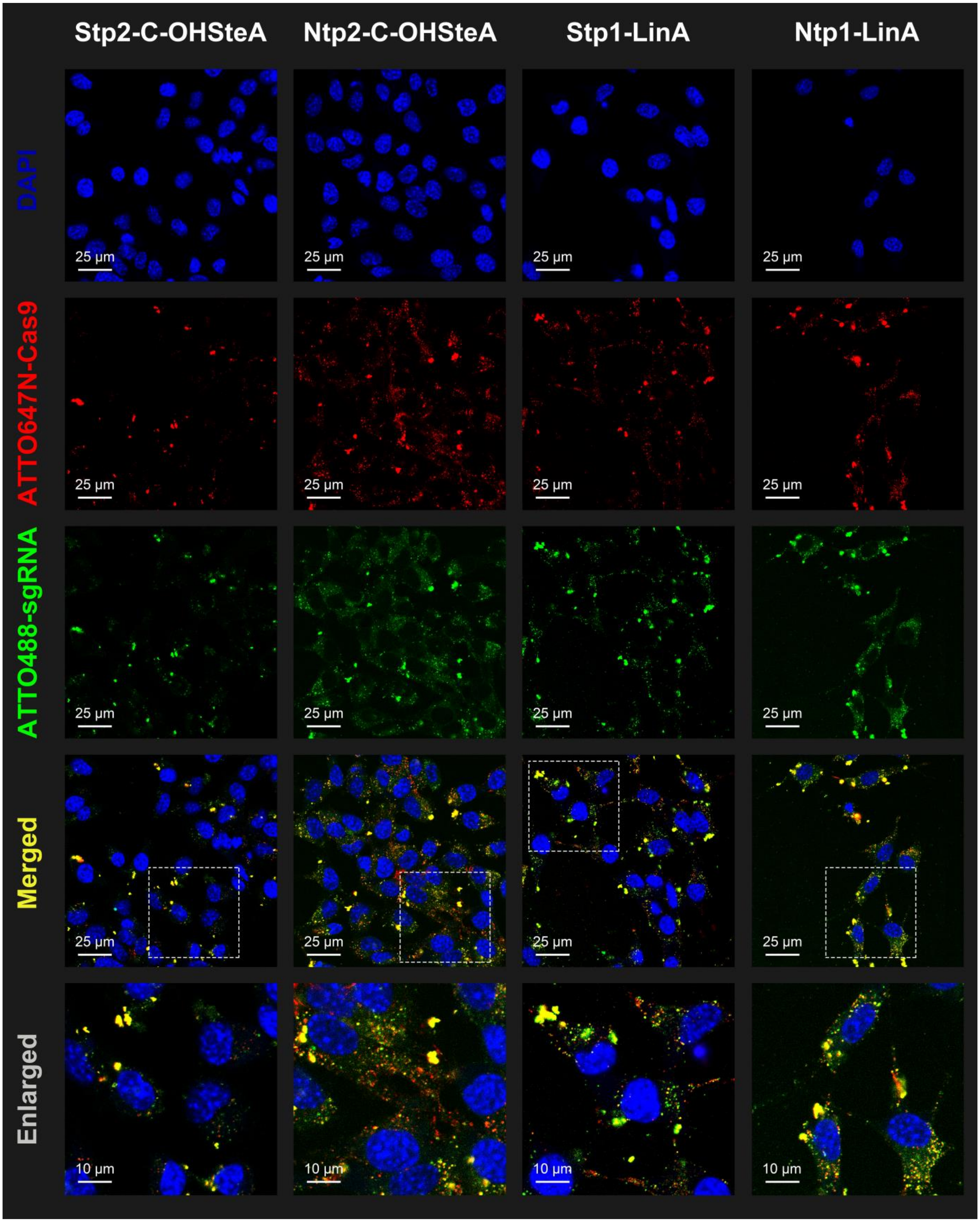

Figure S64. Confocal laser scanning microscopy (CLSM) images of HeLa WT cells 4 h after treatments with different Cas9 RNP nanocarriers containing 20 % of ATTO647N-Cas9/ATTO488-sgRNA at an RNP concentration of 75 nM. Nuclei were stained with DAPI (blue). A subset of the data is shown in Figure 4E of the main manuscript.

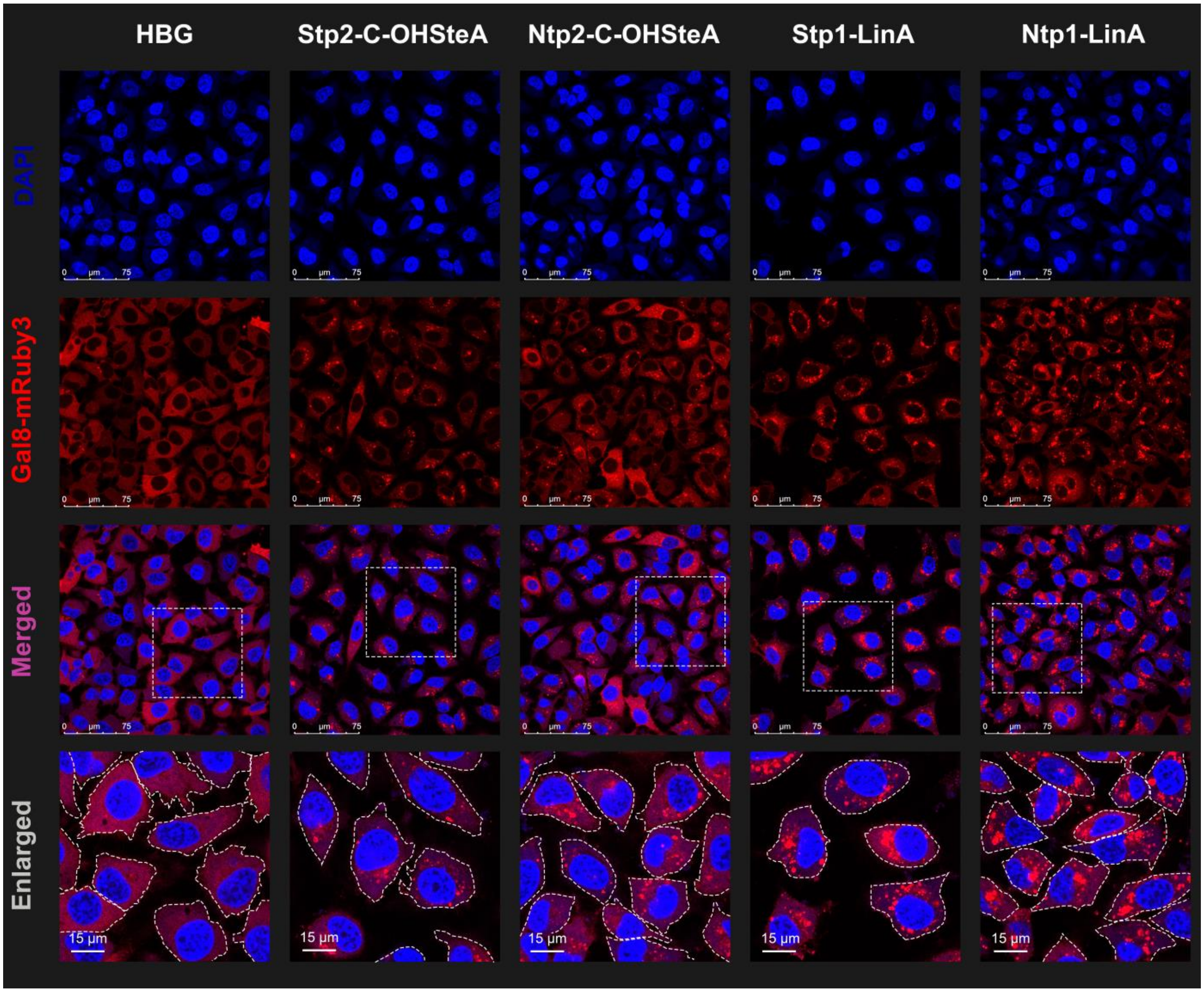

Figure S65. Confocal laser scanning microscopy (CLSM) images of HeLa mRuby3/gal8 cells treated with the selected Cas9 RNP nanocarriers (75 nM RNP) for 4 h. Nuclei were stained with DAPI (blue). Red punctuate mRuby3/gal8 spots indicate damaged endosomes. A subset of the data is shown in Figure 4F of the main manuscript.

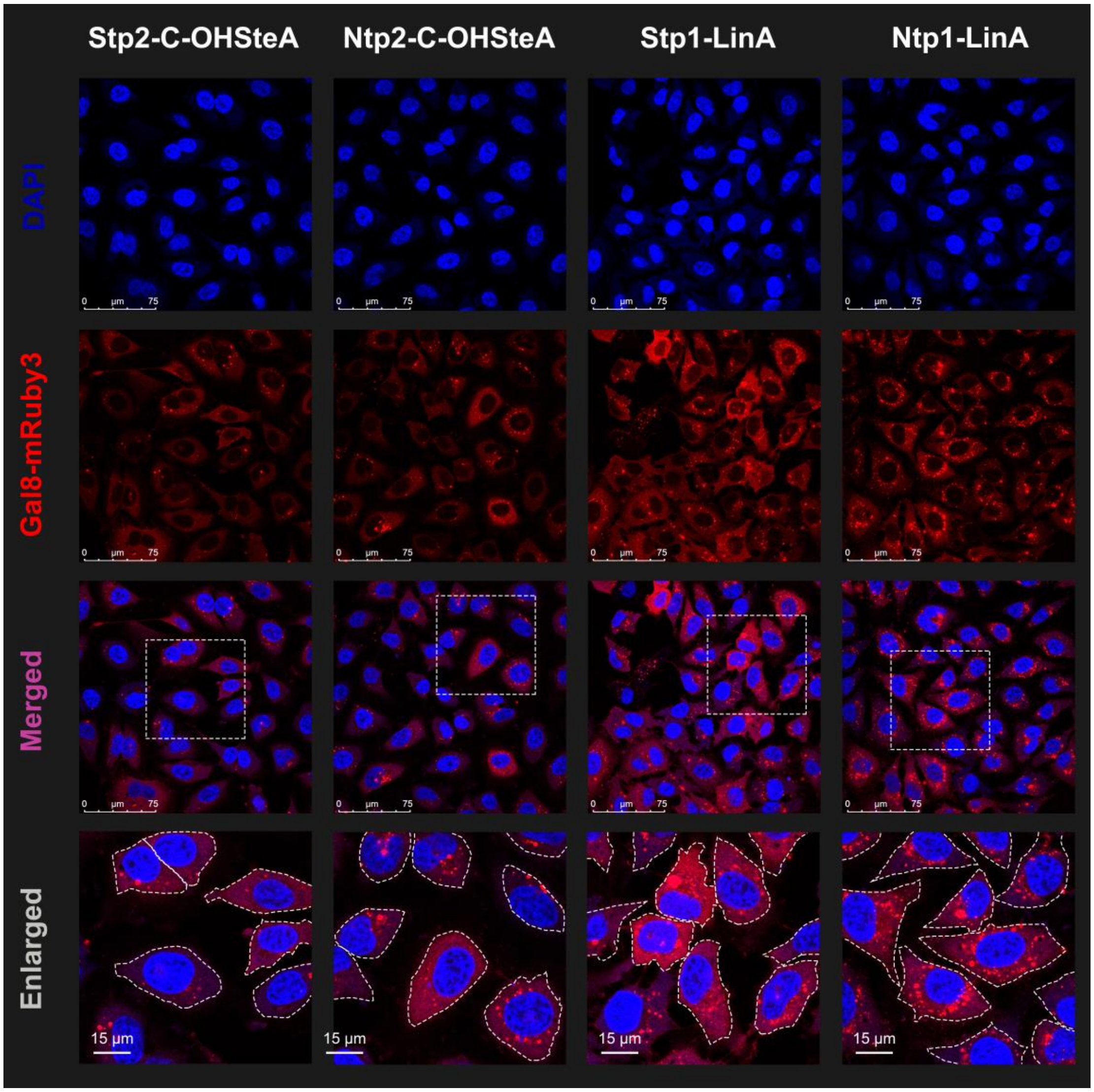

Figure S66. Confocal laser scanning microscopy (CLSM) images of HeLa mRuby3/gal8 cells treated with the selected Cas9 RNP nanocarriers (5 nM RNP) for 4 h. Nuclei were stained with DAPI (blue). Red punctuate mRuby3/gal8 spots indicate damaged endosomes. A subset of the data is shown in Figure 4F of the main manuscript.

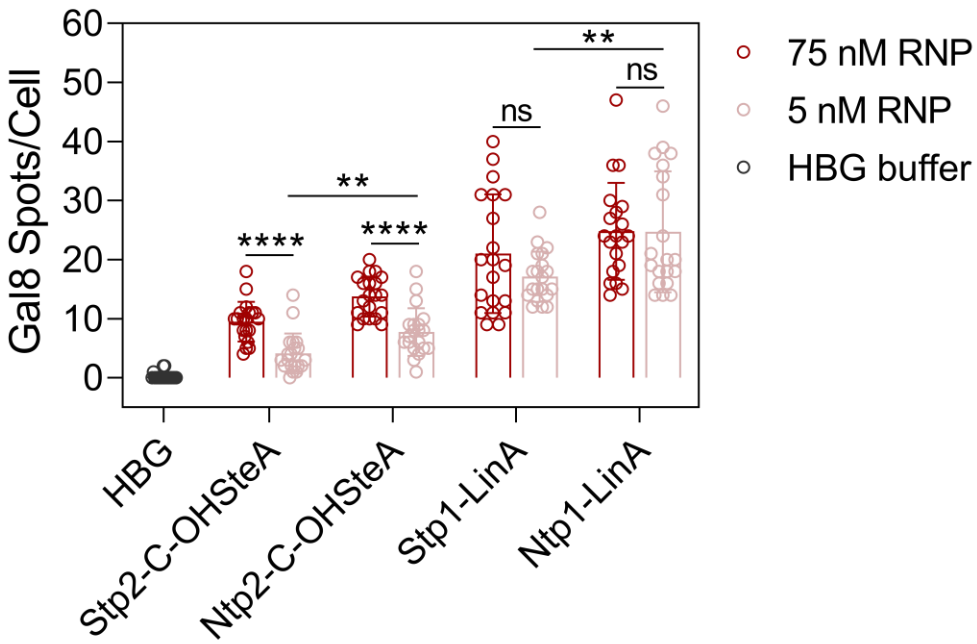

Figure S67. Endosomal disruption level at 4 h of the selected Cas9 RNP nanocarriers quantified by the number of mRuby3/gal8 spots per cell using ImageJ software. Data are presented as means ± SD (n = 20).

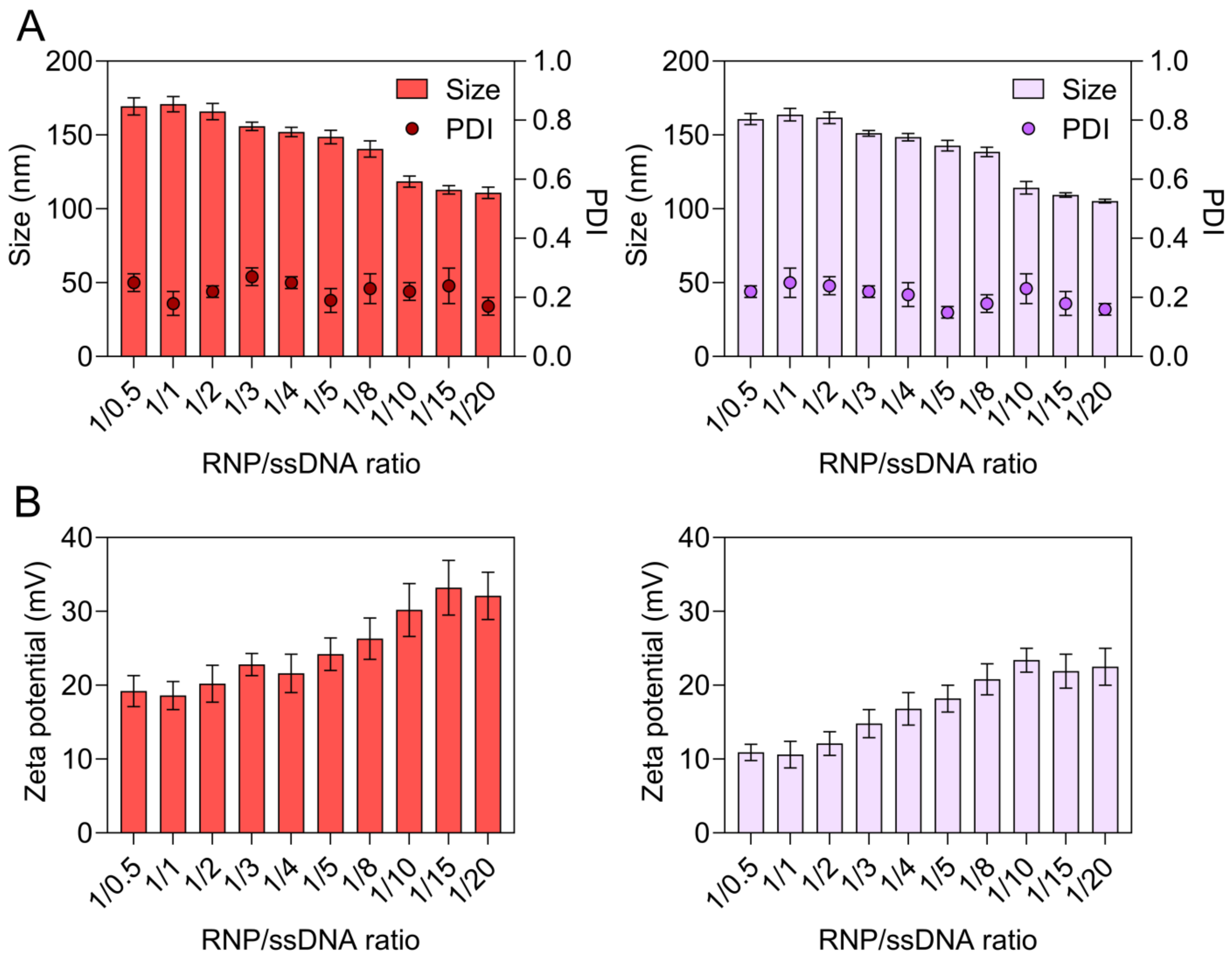

Figure S68. (A) Hydrodynamic particle size (z-average), polydispersity index (PDI) and (B) zeta potential of Cas9 RNP/ssDNA nanocarriers of Stp2-C-OHSteA (left) and TFE-IDAtp1-LinA (right) at a fixed RNP concentration of 75 nM and different RNP/ssDNA ratios of 75 nM. Three technical replicates were measured.

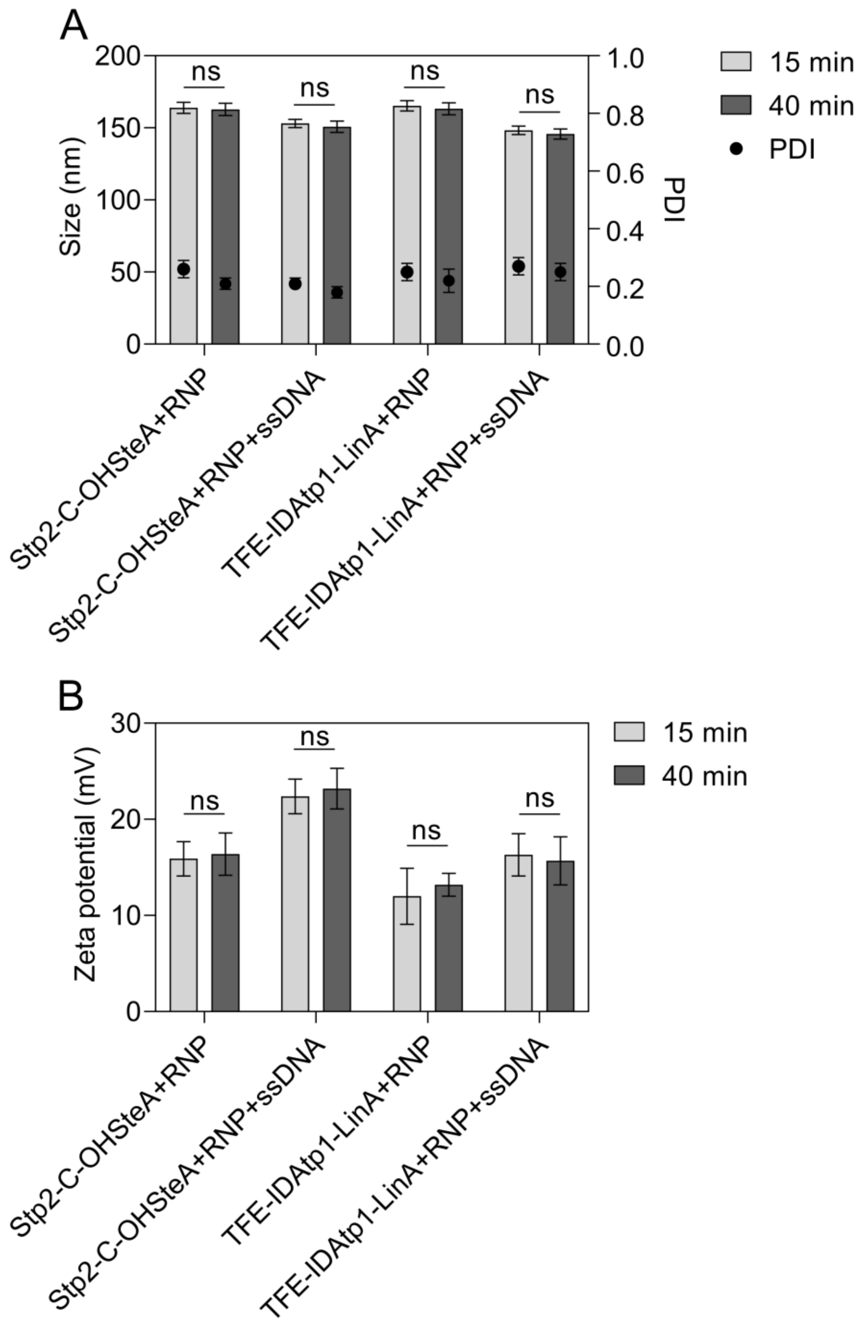

Figure S69. (A) Hydrodynamic particle size (z-average), polydispersity index (PDI) and (B) zeta potential of Cas9 RNP and Cas9 RNP/ssDNA nanocarriers of Stp2-C-OHSteA (left) and TFE-IDAtp1-LinA (right) at a fixed RNP concentration of 75 nM and a RNP/ssDNA ratio of 1/4. The nanocarriers were prepared by complexing Cas9 RNP or Cas9 RNP/ssDNA with the xenopeptides and incubated for 15 min and 40 min. Three technical replicates were measured.

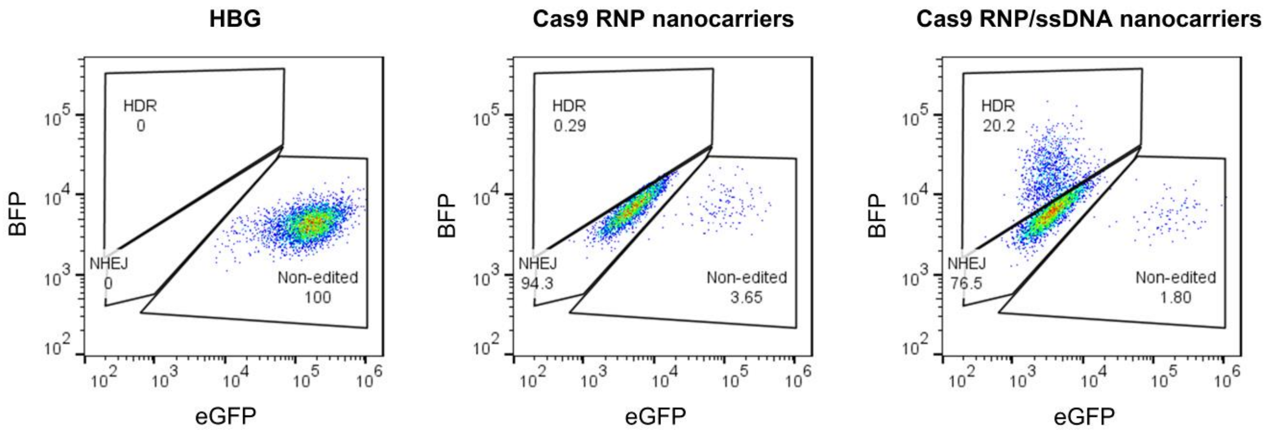

Figure S70. Gating strategy to differentiate non-edited (eGFP positive), NHEJ (eGFP negative), and HDR (BFP positive) cell populations.

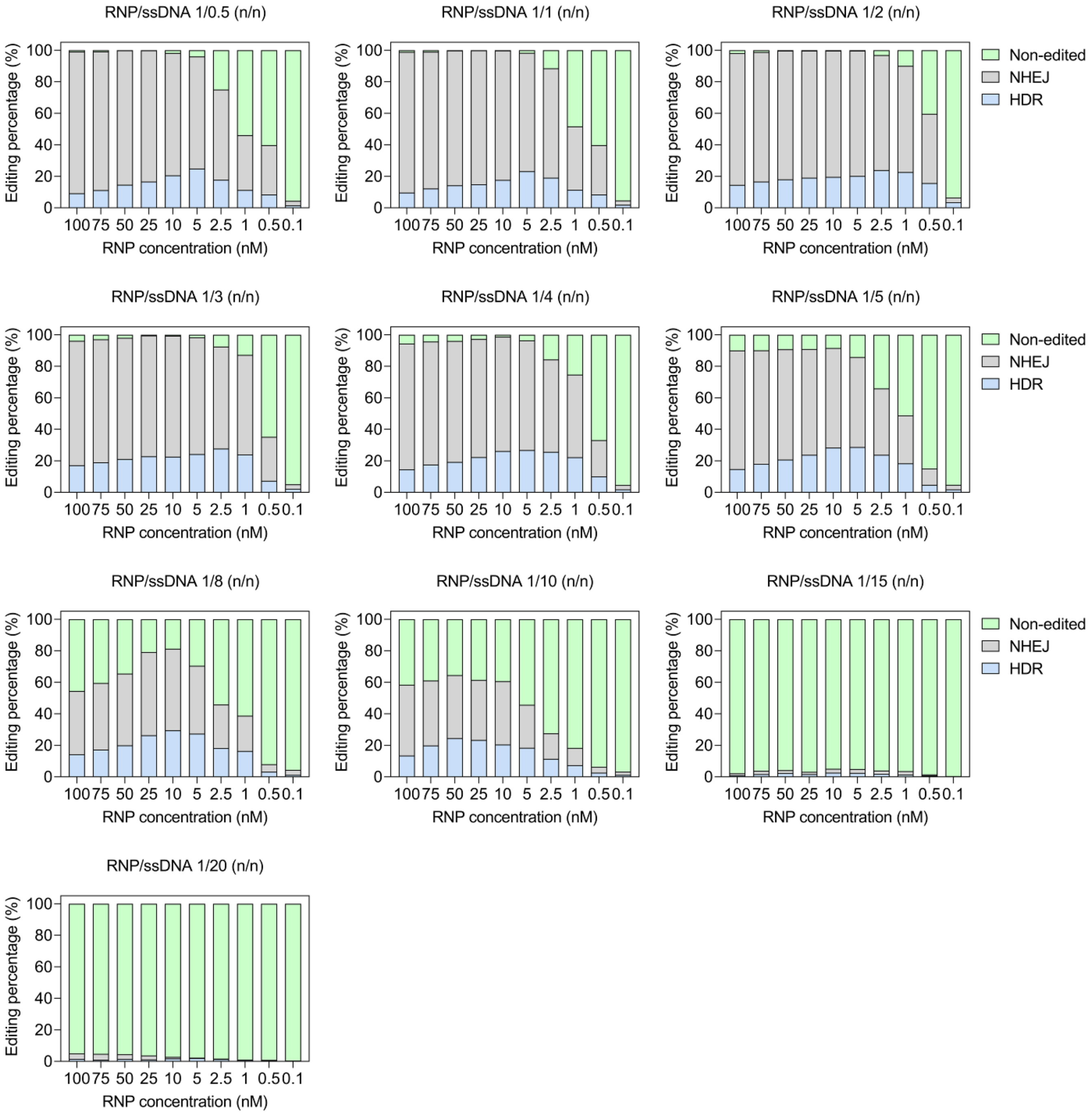

Figure S71. Editing percentage of NHEJ and HDR in HeLa GFPd2 cells treated with **TFE-IDAtp1-LinA** Cas9 RNP/ssDNA nanocarriers at various concentrations and ratios of RNP/ssDNA.

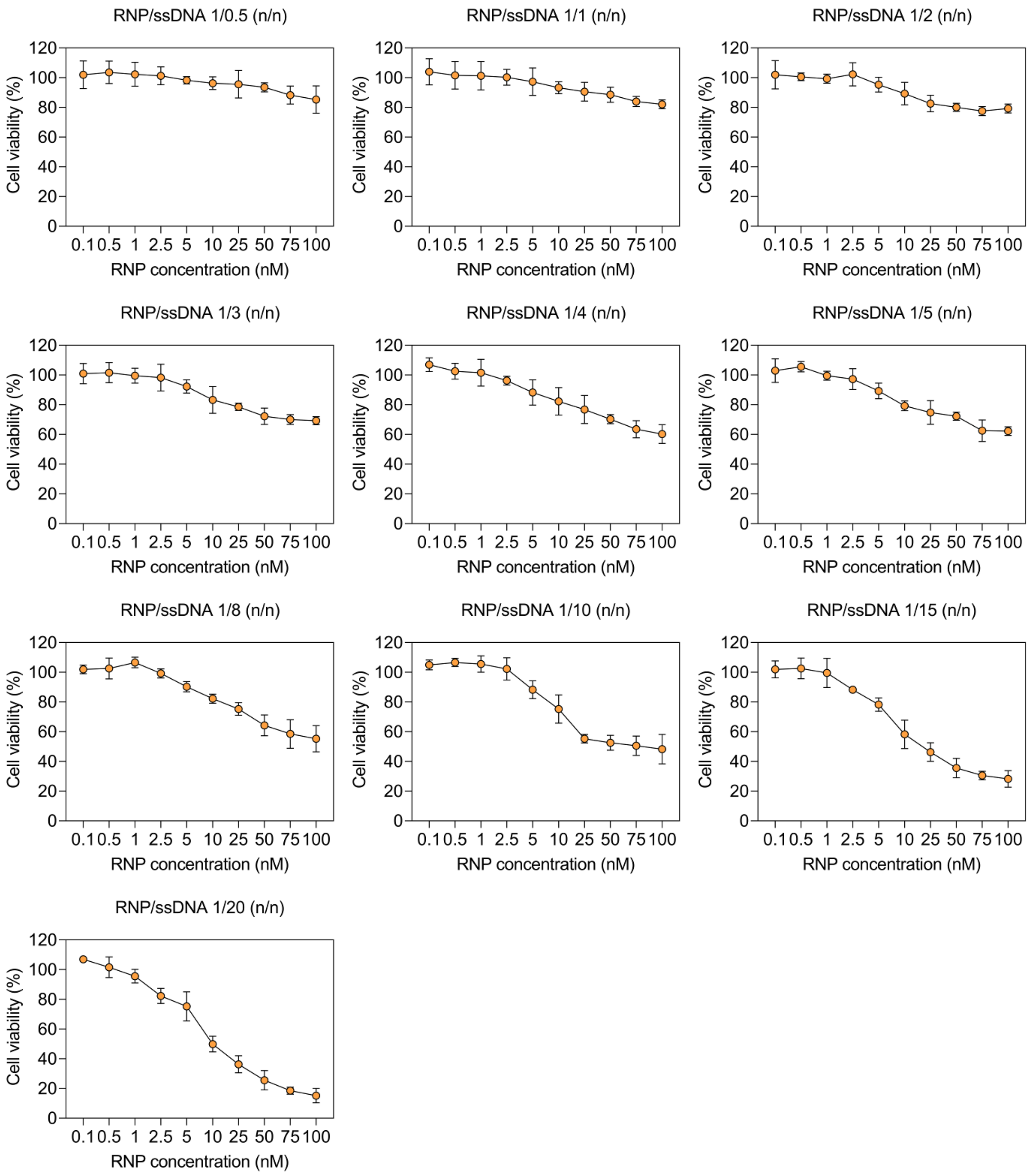

Figure S72. Cell viability of HeLa GFPd2 cells treated with **TFE-IDAtp1-LinA** Cas9 RNP/ssDNA nanocarriers at various concentrations and ratios of RNP/ssDNA.

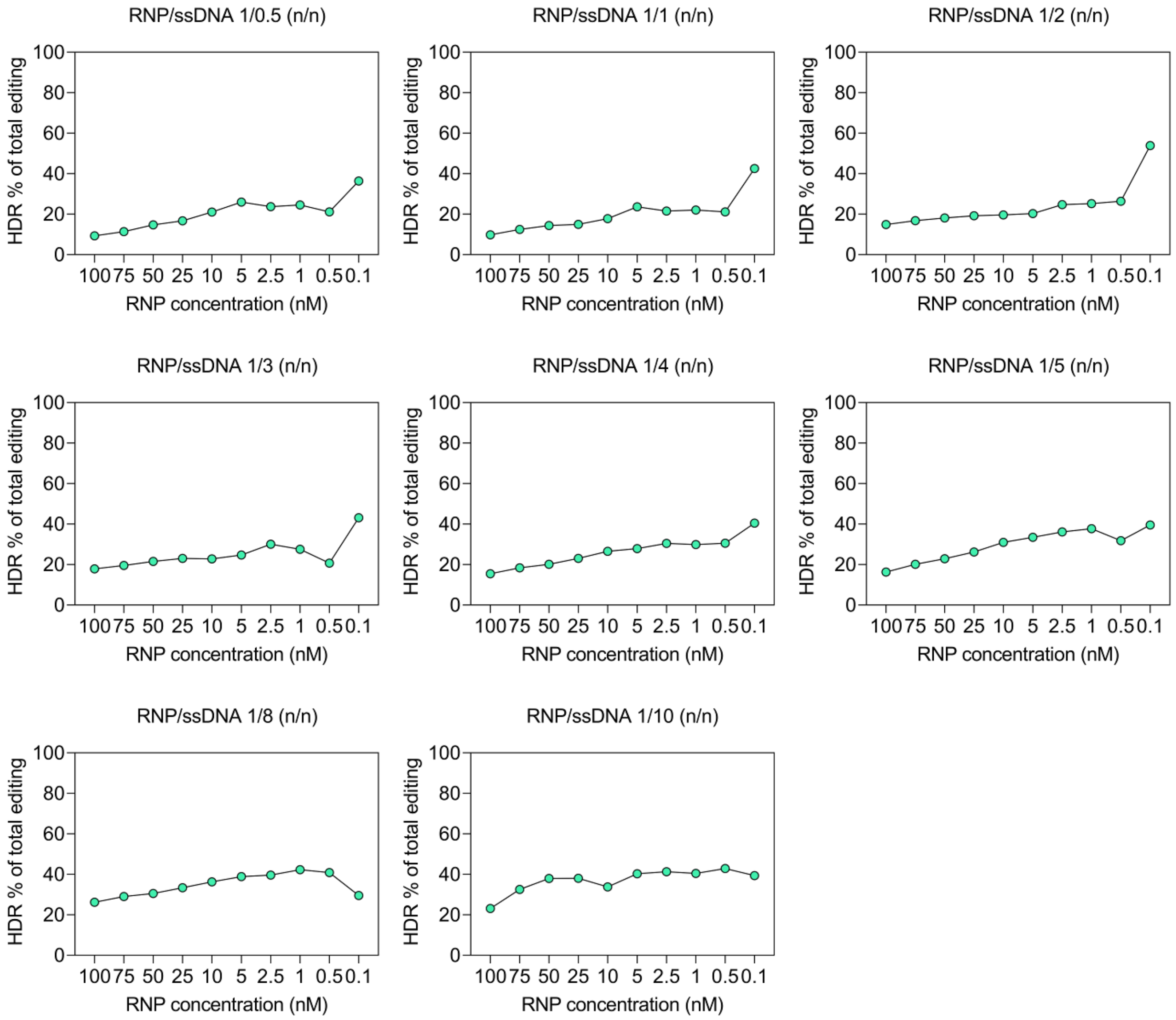

Figure S73. HDR percentage of total editing events in HeLa GFPd2 cells treated with **TFE-IDAtp1-LinA** Cas9 RNP/ssDNA nanocarriers at various concentrations and ratios of RNP/ssDNA.

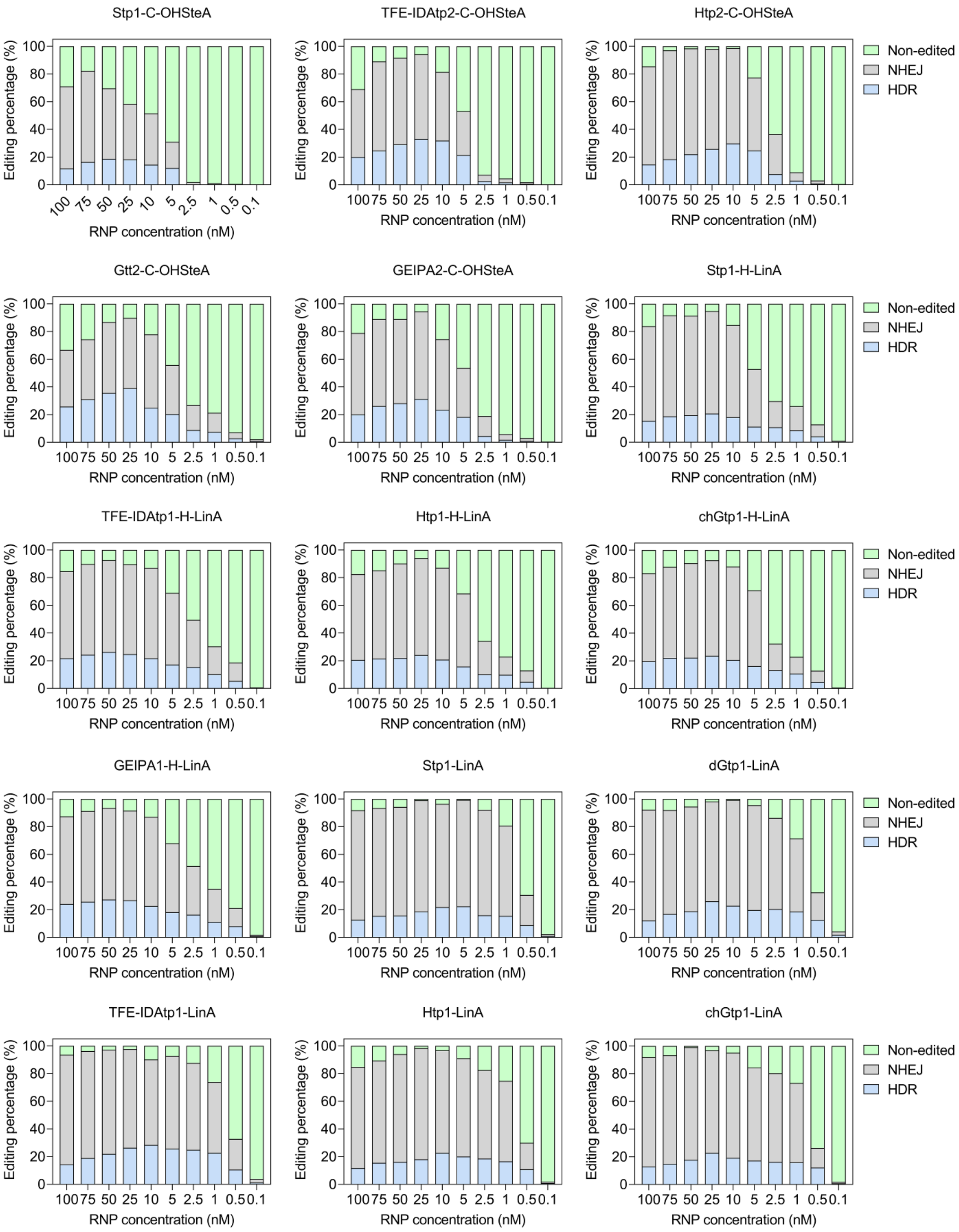

Figure S74. Editing percentage of NHEJ and HDR in HeLa GFPd2 cells treated with different Cas9 RNP/ssDNA nanocarriers at various concentrations (RNP/ssDNA = 1/4).

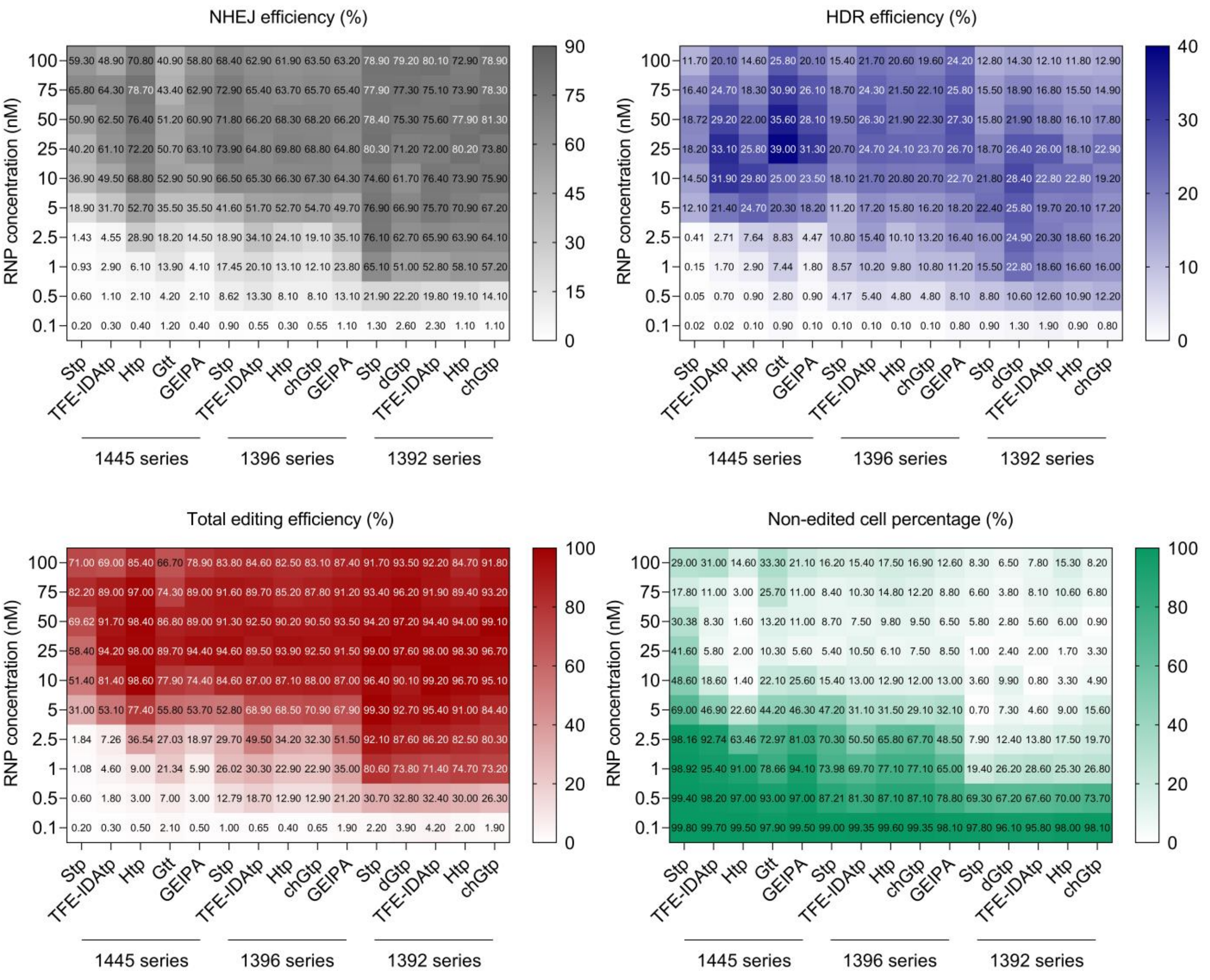

Figure S75. Heat maps of NHEJ, HDR, total edited and non-edited percentages in HeLa GFPd2 cells 48 h after treatment with different Cas9 RNP/ssDNA nanocarriers at various concentrations (RNP/ssDNA = 1/4). A subset of the data is shown in Figure 5C of the main manuscript.

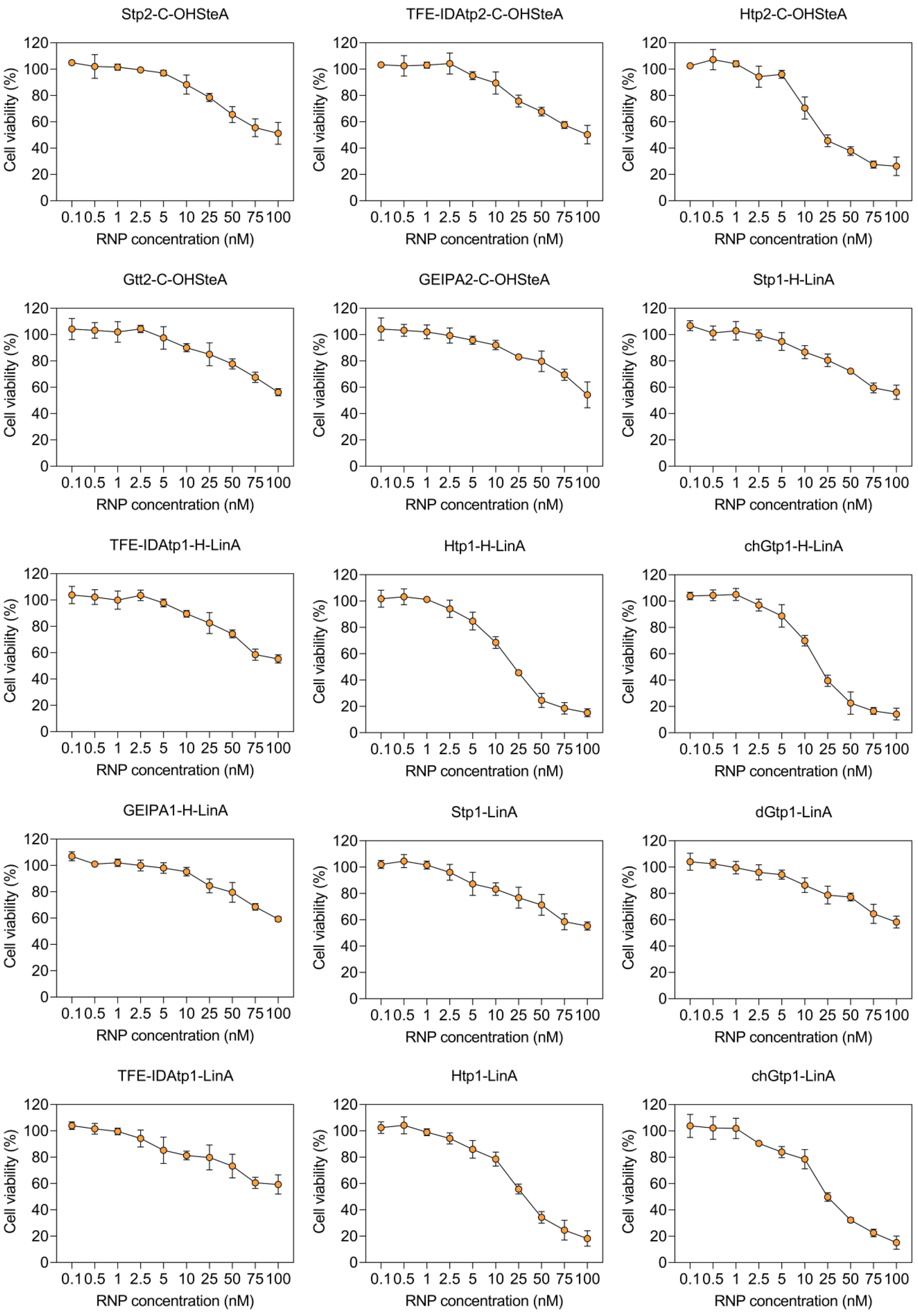

Figure S76. Cell viability of HeLa GFPd2 cells treated with different Cas9 RNP/ssDNA nanocarriers at various concentrations (RNP/ssDNA = 1/4).

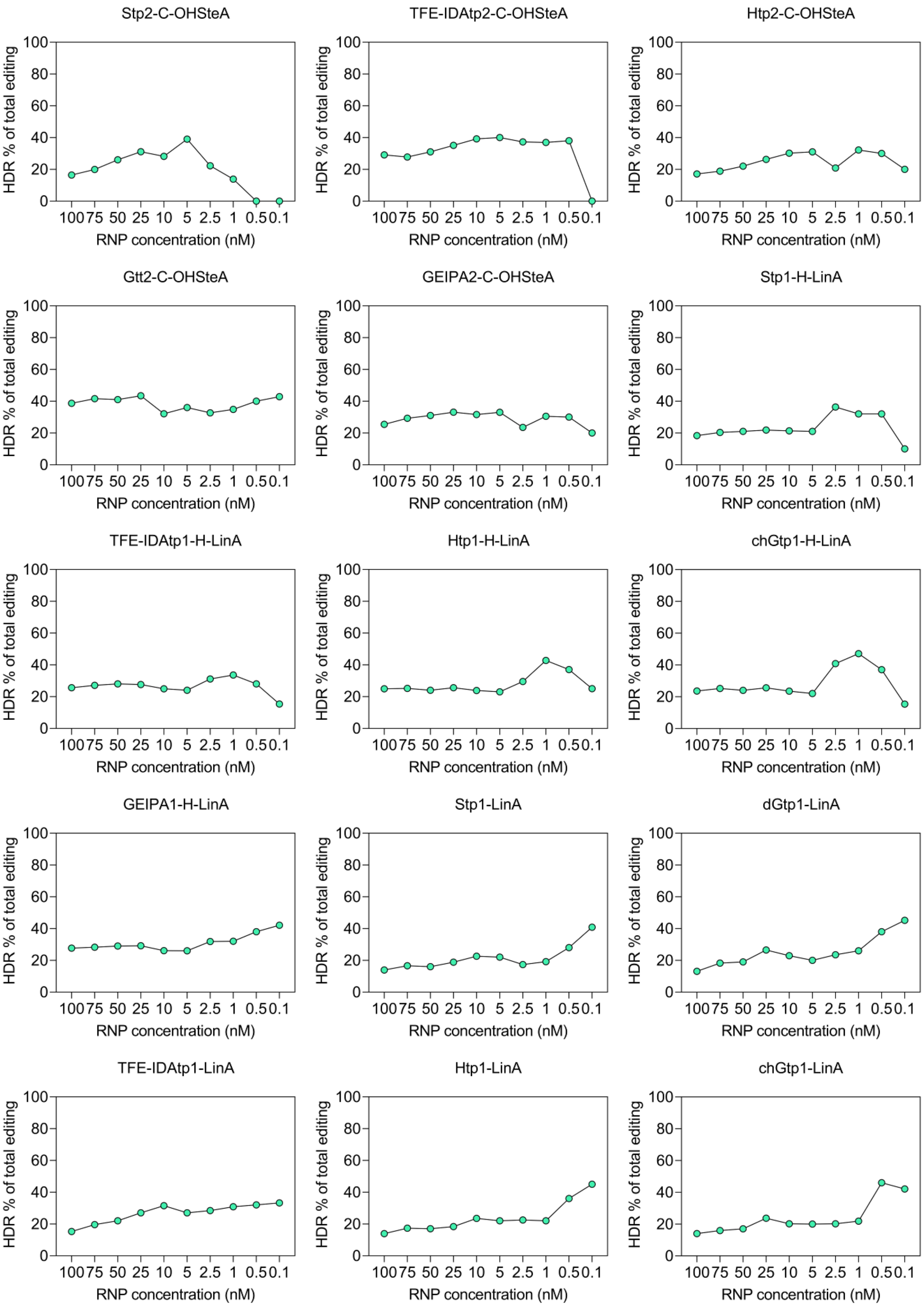

Figure S77. HDR percentage of total editing events in HeLa GFPd2 cells treated with different Cas9 RNP/ssDNA nanocarriers at various concentrations (RNP/ssDNA = 1/4).

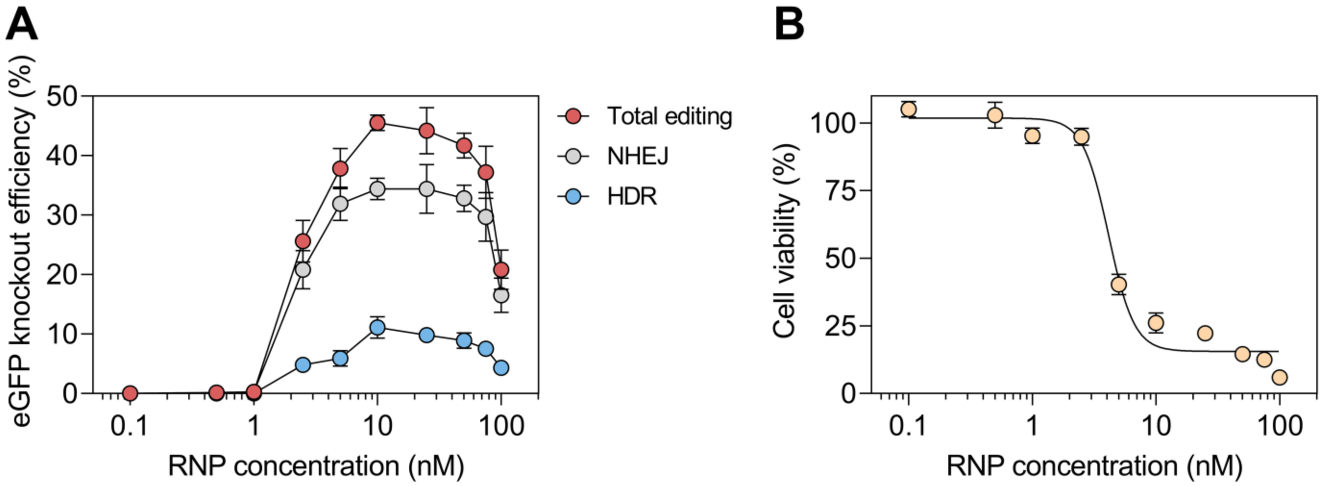

Figure S78. Editing percentage of NHEJ, HDR, and total editing (A) and cell viability (B) of HeLa GFPd2 cells treated with Lipofectamine CRISPRMAX at different concentrations of Cas9/sgGFP2 RNP/ssDNA (RNP/ssDNA = 1/4). Metabolic activity of the cells was determined using a MTT assay. Data are presented as % cell viability with respect to the control cells ± SD (n = 3).

References

(1) Cookson, J.; Vickers, M. S.; Paul, R. L.; Cowley, A. R.; Beer, P. D. Amide functionalised dithiocarbamate ruthenium(II) bis-bipyridyl receptors: A new class of redox-responsive anion sensor. *Inorganica Chimica Acta* **2008**, 361 (6), 1689-1698.

(2) Berchet, G. Methyliminodiacetic acid. *Organic Synth* **1938**, *18*, 56.

(3) a) Salcher, E. E.; Kos, P.; Fröhlich, T.; Badgujar, N.; Scheible, M.; Wagner, E. Sequence-defined four-arm oligo(ethanamino)amides for pDNA and siRNA delivery: Impact of building blocks on efficacy. *Journal of Controlled Release* **2012**, *164* (3), 380-386; b) Schaffert, D.; Badgujar, N.; Wagner, E. Novel Fmoc-Polyamino Acids for Solid-Phase Synthesis of Defined Polyamidoamines. *Organic Letters* **2011**, *13* (7), 1586-1589.

(4) Schaffert, D.; Troiber, C.; Salcher, E. E.; Fröhlich, T.; Martin, I.; Badgujar, N.; Dohmen, C.; Edinger, D.; Kläger, R.; Maiwald, G.; et al. Solid-Phase Synthesis of Sequence-Defined T-, i-, and U-Shape Polymers for pDNA and siRNA Delivery. *Angewandte Chemie International Edition* **2011**, *50* (38), 8986-8989.

(5) a) Kim, H. J.; Ogura, S.; Otabe, T.; Kamegawa, R.; Sato, M.; Kataoka, K.; Miyata, K. Fine-Tuning of Hydrophobicity in Amphiphilic Polyaspartamide Derivatives for Rapid and Transient Expression of Messenger RNA Directed Toward Genome Engineering in Brain. *ACS Central Science* **2019**, *5* (11), 1866-1875; b) Yum, J.; Kim, B. S.; Ogura, S.; Kamegawa, R.; Naito, M.; Yamasaki, Y.; Kim, H. J.; Miyata, K. Fine-tuning of polyaspartamide derivatives with alicyclic moieties for systemic mRNA delivery. *Journal of Controlled Release* **2022**, *342*, 148-156.

(6) Lin, Y.; Wilk, U.; Pöhmerer, J.; Hörterer, E.; Höhn, M.; Luo, X.; Mai, H.; Wagner, E.; Lächelt, U. Folate Receptor-Mediated Delivery of Cas9 RNP for Enhanced Immune Checkpoint Disruption in Cancer Cells. *Small* **2023**, *19* (2), 2205318.

(7) Kuhn, J.; Lin, Y.; Krhac Levacic, A.; Al Danaf, N.; Peng, L.; Höhn, M.; Lamb, D. C.; Wagner, E.; Lächelt, U. Delivery of Cas9/sgRNA Ribonucleoprotein Complexes via Hydroxystearyl Oligoamino Amides. *Bioconjugate Chemistry* **2020**, *31* (3), 729-742.
